# Robustness of radiomics among photon-counting detector CT and dual-energy CT systems: a texture phantom study

**DOI:** 10.1007/s00330-024-10976-1

**Published:** 2024-07-24

**Authors:** Lan Zhu, Haipeng Dong, Jing Sun, Lingyun Wang, Yue Xing, Yangfan Hu, Junjie Lu, Jiarui Yang, Jingshen Chu, Chao Yan, Fei Yuan, Jingyu Zhong

**Affiliations:** 1 Department of Radiology, Ruijin Hospital, Shanghai Jiao Tong University School of Medicine, Shanghai, 200025 China; 2 Department of General Surgery, Shanghai Minimally Invasive Surgery Center, Ruijin Hospital, Shanghai Jiao Tong University School of Medicine, Shanghai, 200025 China; 3 Department of Imaging, Tongren Hospital, Shanghai Jiao Tong University School of Medicine, Shanghai, 200336 China; 4 Department of Epidemiology and Population Health, Stanford University School of Medicine, Stanford, CA 94305 USA; 5 Department of Biomedical Engineering, Boston University, Boston, MA 02215 USA; 6 Department of Science and Technology Development, Ruijin Hospital, Shanghai Jiao Tong University School of Medicine, Shanghai, 200025 China; 7 Department of Surgery, Ruijin Hospital, Shanghai Jiao Tong University School of Medicine, Shanghai, 200025 China; 8 Department of Pathology, Ruijin Hospital, Shanghai Jiao Tong University School of Medicine, Shanghai, 200025 China

**Keywords:** Radiomics, Multidetector computed tomography, Reproducibility of results, Phantoms (imaging)

## Abstract

**Objectives:**

To evaluate the robustness of radiomics features among photon-counting detector CT (PCD-CT) and dual-energy CT (DECT) systems.

**Methods:**

A texture phantom consisting of twenty-eight materials was scanned with one PCD-CT and four DECT systems (dual-source, rapid kV-switching, dual-layer, and sequential scanning) at three dose levels twice. Thirty sets of virtual monochromatic images at 70 keV were reconstructed. Regions of interest were delineated for each material with a rigid registration. Ninety-three radiomics were extracted per PyRadiomics. The test-retest repeatability between repeated scans was assessed by Bland-Altman analysis. The intra-system reproducibility between dose levels, and inter-system reproducibility within the same dose level, were evaluated by intraclass correlation coefficient (ICC) and concordance correlation coefficient (CCC). Inter-system variability among five scanners was assessed by coefficient of variation (CV) and quartile coefficient of dispersion (QCD).

**Results:**

The test–retest repeatability analysis presented that 97.1% of features were repeatable between scan–rescans. The mean ± standard deviation ICC and CCC were 0.945 ± 0.079 and 0.945 ± 0.079 for intra-system reproducibility, respectively, and 86.0% and 85.7% of features were with ICC > 0.90 and CCC > 0.90, respectively, between different dose levels. The mean ± standard deviation ICC and CCC were 0.157 ± 0.174 and 0.157 ± 0.174 for inter-system reproducibility, respectively, and none of the features were with ICC > 0.90 or CCC > 0.90 within the same dose level. The inter-system variability suggested that 6.5% and 12.8% of features were with CV < 10% and QCD < 10%, respectively, among five CT systems.

**Conclusion:**

The radiomics features were non-reproducible with significant variability in values among different CT techniques.

**Clinical relevance statement:**

Radiomics features are non-reproducible with significant variability in values among photon-counting detector CT and dual-energy CT systems, necessitating careful attention to improve the cross-system generalizability of radiomic features before implementation of radiomics analysis in clinical routine.

**Key Points:**

- *CT radiomics stability should be guaranteed before the implementation in the clinical routine.*
- *Radiomics robustness was on a low level among photon-counting detectors and dual-energy CT techniques.*
- *Limited inter-system robustness of radiomic features may impact the generalizability of models.*

## Introduction

Radiomics derives objective and quantifiable imaging biomarkers from medical images to provide insights beyond subjective and qualitative image analysis [1, 2]. This pixel-level image analysis approach allows additional in-depth features that are invisible to the human naked eye, creates new possibilities, and promises for fostering the big data trends in healthcare [3, 4]. Radiomics has shown its possible capabilities in tumor classification [5], response prediction [6], and risk stratification [7] in oncologic imaging. Additionally, the potential of radiomics has also been presented in non-oncologic diseases, such as coronary plaque [8], pancreatitis [9], pneumonia [10], Crohn’s disease [11], kidney stone [12], etc. There is a huge number of academic papers on radiomics research, and it is still increasing [13, 14]. However, the radiomics analysis has not been widely implanted into clinical routine [15–18], since it is currently not supported by adequate scientific evidence.

One of the most significant challenges of radiomics analysis is the lack of robustness [19–24]. The influence of acquisition and reconstruction parameters have been demonstrated to have impacts on the robustness of CT radiomics features in conventional single-energy CT (SECT) systems, including scan system, radiation dose level, voxel size, reconstruction algorithm, reconstruction kernel, etc. [25–27]. The dual-energy CT (DECT) systems have introduced more potential influencing factors on radiomics features, as there is a heterogeneous technique to realize the DECT scans, such as dual-source dual-energy CT (dsDECT), rapid kV-switching dual-energy CT (rsDECT), dual-layer dual-energy CT (dlDECT), sequential scanning dual-energy CT (ssDECT), and split filter dual-energy CT systems [28]. The radiomics features were non-reproducible neither between the conventional CT and DECT scans, nor across different DECT techniques [29–33], even though the parameters were carefully adjusted. The photon-counting detector CT (PCD-CT) system are believed to allow high feature stability and better characterization of disease [34–39] since it directly converses photons into electric pulses without the intermediate step of visible light [40]. Nevertheless, it may lead to extra differences in radiomics features due to higher image resolution compared to traditional energy-integrating CT systems [34–36]. As the PCD-CT systems are not widely available nowadays, it is important to determine whether the images from generated using different CT techniques are consistent enough for radiomics analysis.

Therefore, this study aimed to evaluate robustness of radiomics features on texture phantom scans using one PCD-CT system and four DECT systems.

## Materials and methods

**Fig. 1:**
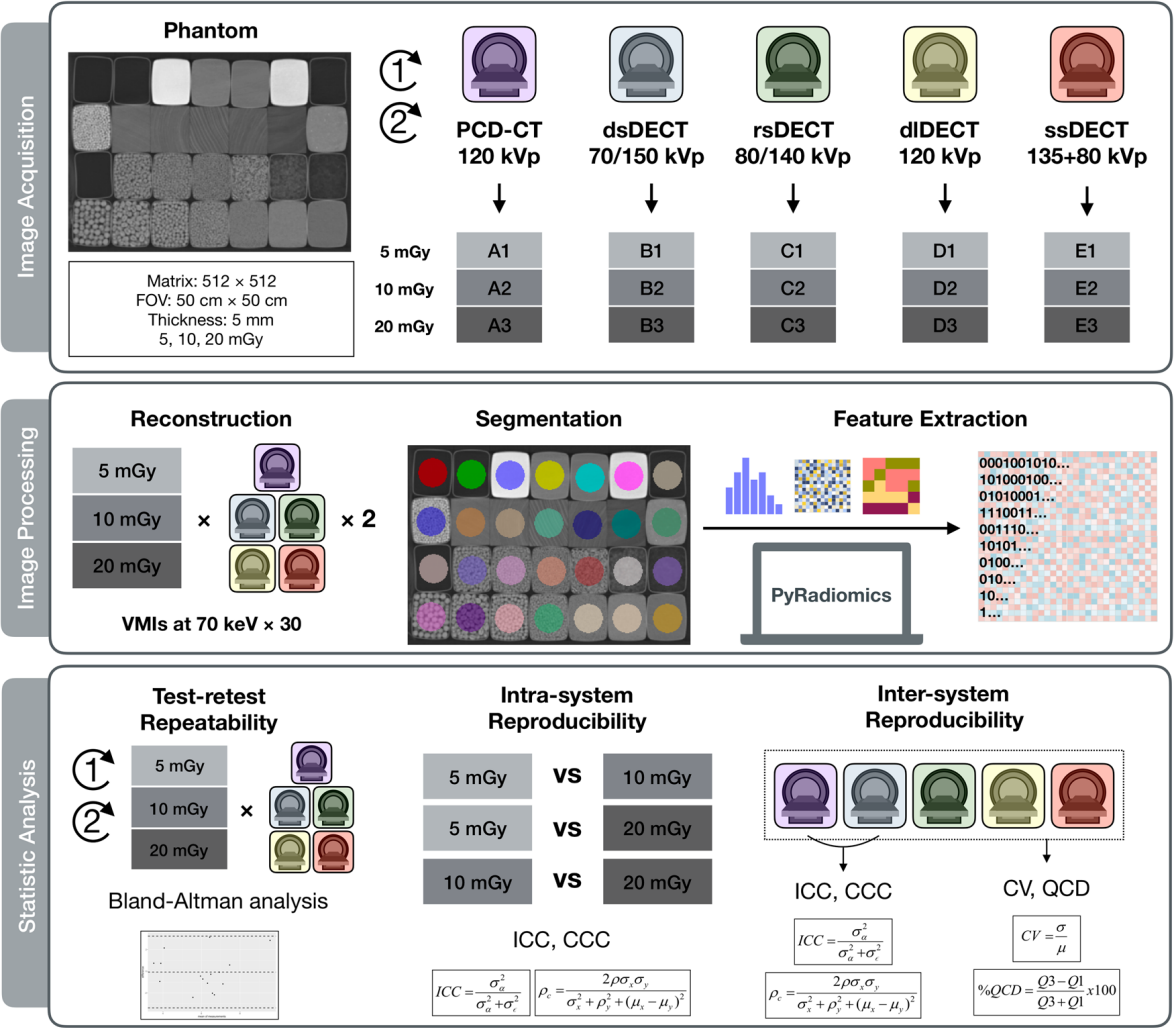
Study workflow. This study consisted of three steps: image acquisition, image processing, and statistical analysis. A homemade texture phantom was scanned on one PCT-CT system and four types of DECT systems, at three dose levels of 5, 10, and 20 mGy. The raw data was generated into VMIs at 70 keV. Pyradiomics was employed to extract 18 first-order and 75 texture radiomics features from ROIs segmented with a rigid registration. Test-retest repeatability between repeated scans was assessed by Bland-Altman analysis. The intra-system reproducibility between dose levels, and inter-system reproducibility within the same dose level, were evaluated by ICC and CCC. Inter-system variability among five scanners was estimated by CV and QCD

The workflow of the study is presented in Fig. 1. The institution’s ethics approval and written informed consent are not required since this was a phantom study.

### Phantom

**Fig. 2:**
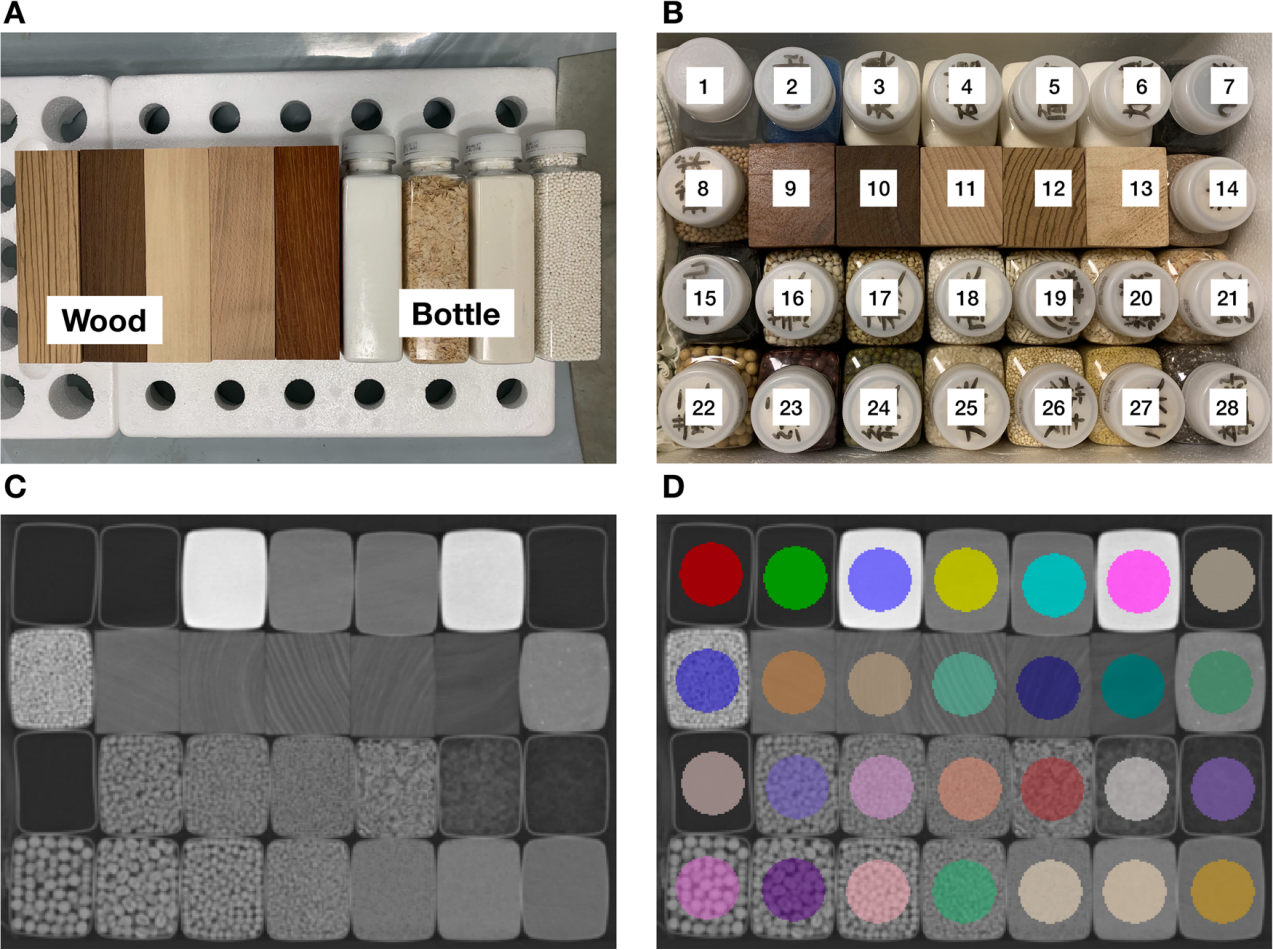
Phantom construction and image segmentation. **A** The inserts of the homemade phantom were made of wood blocks and bottles filled with different materials. **B** The inserts were placed in a foam plastic box and kept stable across all scans. The inserts used in the current study were: (1) air, (2) mesoporous sponge, (3) iodize-free salt, (4) granulated sugar, (5) flour, (6) iodized salt, (7) coarse-pore sponge, (8) nutritive soil for succulent plants, (9) rosewood, (10) chicken wing wood, (11) beechwood, (12) zebra wood, (13) basswood, (14) sand, (15) microporous sponge, (16) coix seed, (17) buckwheat, (18) sago, (19) cat litter, (20) oat, (21) sawdust, (22) soybean, (23) red bean, (24) mung bean, (25) rice, (26) quinoa, (27) millet, and (28) chia seed. **C** CT image of a representative axial slice of each material in the phantom. **D** A total of twenty-eight regions of interest were manually contoured on the reference scan and then copied to all other scans with a rigid registration

We established a texture phantom consisting of twenty-eight different materials as shown in Fig. 2. There were five wood blocks and twenty-three bottles filled with different materials [25, 41]. The wood block was a cuboid with a size of 150 mm × 55 mm × 45 mm. The cuboid part of the bottle was with a size of 130 mm × 55 mm ×45 mm. The cuboid part bottle was filled with materials as tightly as possible. These materials were selected to give us varying textures. The materials were positioned to avoid beam-hardening artifacts and were kept unchanged throughout all the scans in the study. The details of the set-ups of the texture phantom are presented in Supplementary Note S1.

### Image acquisition and reconstruction

The phantom was scanned on five CT scanners including one PCD-CT system (NAEOTOM Alpha, Siemens Healthineers) and four dual-energy CT systems (dsDECT, SOMATOM Force, Siemens Healthineers; rsDECT, Revolution CT Apex, GE Healthcare; dlDECT, Hawk spectral CT, Philips Healthcare; and ssDECT, Aquilion ONE, Canon Medical Systems) from two centers, respectively. The comparable acquisition and reconstruction parameters for scans are presented in Table 1. Each scan was repeated several minutes apart to allow scan-rescan repeatability assessment. The field of view (500 mm × 500 mm), reconstruction matrix (512 × 512), and slice thickness (5 mm) were kept unchanged to allow stable voxel size. The milliamperage, rotation time, and pitch value were adjusted to meet the volume CT dose index of 5, 10, and 20 mGy. The tube voltage, iteration reconstruction method, and reconstruction kernel were selected to present a typical abdomen-pelvic examination. All the images were reconstructed into virtual monoenergetic images (VMIs) at the energy level of 70 keV per vendor-specific workstations relying on comparable linear energy blending approaches. The kilo-electron volt level of 70 keV was chosen because this energy level was used as a clinical standard of reference at our institution and has been suggested to be comparable to conventional images [42–44].

**Table 1:** CT acquisition and reconstruction parameters

No	Vendor	Scanner	Type	Tube voltage (kVp)	Milliamperage (mA or mAs)	Rotation time (sec)	Pitch	Bean collimation (mm)	Volume CT dose index (mGy)	Iteration method	Reconstruction kernel
A1	SIEMENS	NAEOTOM Alpha	PCD-CT	120	63	0.5	1	144 × 0.4	4.98	QIR 2/4	Br40
A2	SIEMENS	NAEOTOM Alpha	PCD-CT	120	127	0.5	1	144 × 0.4	10.00	QIR 2/4	Br40
A3	SIEMENS	NAEOTOM Alpha	PCD-CT	120	253	0.5	1	144 × 0.4	20.00	QIR 2/4	Br40
B1	SIEMENS	SOMATOM Force	dsDECT	70/150	213/53	1.0	1	64 × 0.6	4.98	ADMIRE 2/5	Br40
B2	SIEMENS	SOMATOM Force	dsDECT	70/150	427/107	1.0	1	64 × 0.6	10.01	ADMIRE 2/5	Br40
B3	SIEMENS	SOMATOM Force	dsDECT	70/150	853/213	1.0	1	64 × 0.6	20.01	ADMIRE 2/5	Br40
C1	GE	Revolution Apex	rsDECT	80/140	145^a^	0.6	0.984	64 × 0.625	4.95	Asir-V 40%	Standard
C2	GE	Revolution Apex	rsDECT	80/140	335^a^	0.6	0.984	64 × 0.625	10.05	Asir-V 40%	Standard
C3	GE	Revolution Apex	rsDECT	80/140	370^a^	1.0	0.984	64 × 0.625	19.75	Asir-V 40%	Standard
D1	PHILIPS	Hawk spectral CT	dlDECT	120	65	0.5	1	128 × 0.625	5.00	Spectral 3/6	Standard (B)
D2	PHILIPS	Hawk spectral CT	dlDECT	120	129	0.5	1	128 × 0.625	10.00	Spectral 3/6	Standard (B)
D3	PHILIPS	Hawk spectral CT	dlDECT	120	259	0.5	1	128 × 0.625	20.00	Spectral 3/6	Standard (B)
E1	CANON	Aquilion ONE	ssDECT	135 + 80	20 + 110^a^	0.75	0.976	160 × 0.5	5.30	AIDR 3D Standard	FC17
E2	CANON	Aquilion ONE	ssDECT	135 + 80	30 + 170^a^	1.0	0.976	160 × 0.5	10.70	AIDR 3D Standard	FC17
E3	CANON	Aquilion ONE	ssDECT	135 + 80	90 + 510^a^	0.6	0.976	160 × 0.5	19.50	AIDR 3D Standard	FC17

Robustness of radiomics among photon-counting detector CT and dual-energy CT systems: a texture phantom study

*PCD-CT* photon-counting detector CT, *DECT* dual-energy CT, *dsDECT* dual-source dual-energy CT, *rsDECT* rapid kV-switching dual-energy CT, *dlDECT* dual-layer dual-energy CT, *ssDECT* sequential scanning DECT, *QIR* quantum iterative reconstruction, *ADMIRE* advanced modeled iterative reconstruction, *Asir-V* adaptive statistical iterative reconstruction-V, *AIDR 3D* adaptive iterative dose reduction 3D

^a^ Represents mA not mAs for GE medical systems and Canon medical systems

### Segmentation and feature extraction

The images were exported in Digital Imaging and Communications in Medicine (DICOM) format, and then converted to Neuroimaging Informatics Technology Initiative (NIFTI) format using MRIcroGL version 1.2.20220720b (https://www.nitrc.org/frs/?group_id=889). The images were loaded into ITK-SNAP version 4.0.2 (http://www.itksnap.org/pmwiki/pmwiki.php) for segmentation by a radiologist with 5 years of experience in radiology and radiomics phantom research [30–33]. Twenty-eight regions of interest (ROIs) of 35 pixels (approximately 34 mm) in diameter were put at the center of each wood block or bottle with different materials following a rigid registration to avoid unexpected variations [25]. The ROIs were placed on one reference scan and then copied to other scans. Each ROI was copied to the continuous middle five layers of the image of each wood block or bottle with different materials for radiomics feature extraction. We did not perform any pre-processing steps before the feature extraction. Python version 3.12.1 (https://www.python.org) with PyRadiomics package version 3.0.1 (https://pyradiomics.readthedocs.io/en/latest/) was used to extract 18 first-order features and 75 texture features, namely 24 gray-level co-occurrence matrix (GLCM), 14 gray-level run length matrix (GLRLM), 16 gray-level zone length matrix (GLZLM), 16 gray-level dependence matrix (GLDM), and 5 neighborhood gray-tone difference matrix (NGTDM) features [45]. The 26 shape features were not included since the ROIs were fixed in this study. The settings of feature extraction and calculated features are presented in Supplementary Note S2.

### Radiomics robustness analysis

The test-retest repeatability was assessed using the middle five layers of images from two repeating scans with unchanged acquisition and reconstruction parameters from the same system. The intra-system reproducibility between different dose levels was evaluated between images from 5 vs. 10 mGy, 5 vs. 20 mGy, and 10 vs. 20 mGy scans, respectively, within the same system. The inter-system reproducibility was calculated using images acquired at three dose levels of 5, 10, and 20 mGy scans, respectively, between each two out of the five CT systems. The inter-system variability at three dose levels of 5, 10, and 20 mGy scans was estimated across five systems for each of the twenty-eight materials. The robustness of radiomics was also analyzed according to five feature types. The signal-to-noise ratio of each scan was calculated.

### Statistical analysis

The statistical analysis was performed using R language version 4.1.3 (https://www.r-project.org/) within RStudio version 1.4.1106 (https://posit.co/). The mean relative change of the radiomics features across the different datasets was calculated. The test-retest repeatability was assessed using Bland-Altman analysis with a cutoff of 90% [46, 47]. The intra-system reproducibility between different dose levels, and inter-system reproducibility within the same dose level, were evaluated by intraclass correlation coefficient (ICC) of two-way mixed effects, single rater, absolute agreement type [48] and concordance correlation coefficient (CCC) [49]. The inter-system variability among the five systems was assessed by coefficient of variation (CV) [50] and quartile coefficient of dispersion (QCD) [51]. The ICC and CCC values were interpreted as follows: poor, < 0.50; moderate, 0.50–0.75; good, 0.75–0.90; or excellent, ≥ 0.90, while the CV and QCD values were interpreted as follows: acceptable, < 10%; moderate but still adequate, 11%–20%; and too high and inadequate, ≥ 20% [52].

## Results

### Test-retest repeatability of radiomics features

The percentage of repeatable features ranged from 82.8 to 100.0%, and the overall percentage ± standard deviation of repeatable features was 97.1 ± 6.2%, according to Bland-Altman analysis (Supplementary Table S1 and Supplementary Fig. S1). The signal-to-noise ratio of each scan (Supplementary Table S2) and the mean relative change of the radiomics feature in reference to PCD-CT (Supplementary Table S3) were calculated. The results were also summarized according to five feature types (Supplementary Tables S4 to S7).

### Intra-system reproducibility among three dose levels

**Fig. 3:**
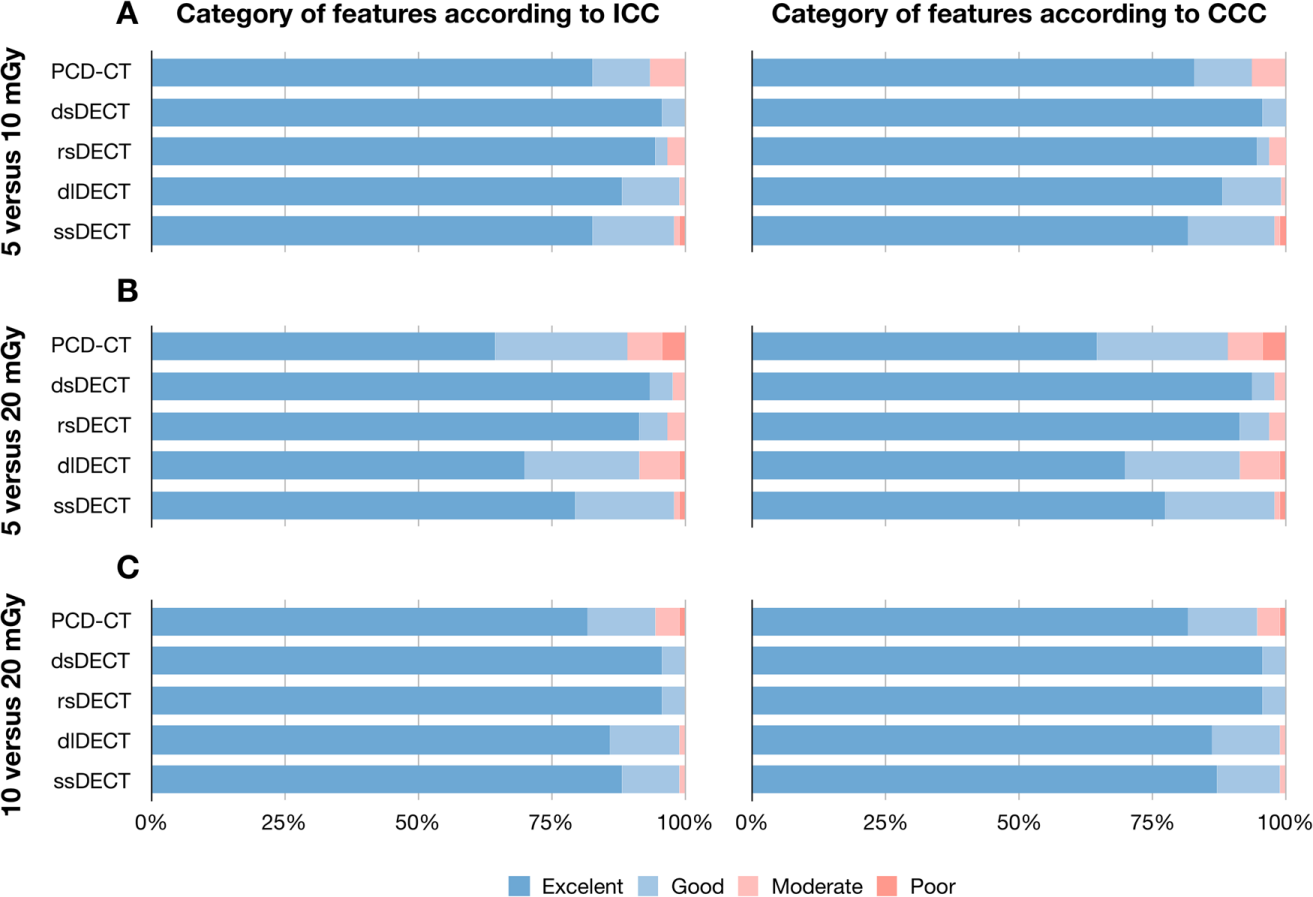
Percentage of robust radiomics features according to intra-system reproducibility. The percentage of robust features in terms of intra-system reproducibility among three dose levels of (**A**) 5 vs. 10 mGy, (**B**) 5 vs. 20 mGy, and (**C**) 10 vs. 20 mGy, according to ICC and CCC values. The ICC and CCC values were interpreted as follows: poor, < 0.50; moderate, 0.50–0.75; good, 0.75–0.90; or excellent, ≥ 0.90

The overall mean ± standard deviation ICC and CCC values for intra-system reproducibility were 0.945 ± 0.079 and 0.945 ± 0.079, respectively (Table 2), and the percentage of features with ICC > 0.90 and CCC > 0.90 were 86.0% and 85.7%, respectively (Fig. 3). The mean ± standard deviation ICC and CCC values of five CT systems ranged from 0.916 ± 0.112 to 0.978 ± 0.041, and from 0.915 ± 0.112 to 0.977 ± 0.041, respectively. The percentage of features with ICC > 0.90 and CCC > 0.90 ranged from 76.3% to 95.0%, and from 76.3% to 95.0%, respectively. The results for each feature were summarized (Supplementary Fig. S2).

**Table 2:** Intra-system reproducibility among three dose levels

Scanner	5 vs. 10 mGy		5 vs. 20 mGy		10 vs. 20 mGy	
	ICC	CCC	ICC	CCC	ICC	CCC
A	0.930 ± 0.079	0.929 ± 0.079	0.888 ± 0.143	0.888 ± 0.143	0.932 ± 0.100	0.932 ± 0.100
B	0.980 ± 0.040	0.980 ± 0.040	0.970 ± 0.047	0.970 ± 0.047	0.983 ± 0.035	0.983 ± 0.035
C	0.974 ± 0.051	0.974 ± 0.052	0.964 ± 0.058	0.964 ± 0.058	0.981 ± 0.038	0.981 ± 0.038
D	0.943 ± 0.058	0.942 ± 0.058	0.905 ± 0.115	0.904 ± 0.115	0.947 ± 0.059	0.946 ± 0.059
E	0.922 ± 0.067	0.922 ± 0.068	0.921 ± 0.072	0.921 ± 0.072	0.935 ± 0.047	0.935 ± 0.047

The data were presented as mean ± standard deviation

*ICC* intraclass correlation coefficient, *CCC* concordance correlation coefficient. *A* PCD-CT system (NAEOTOM Alpha, Siemens Healthineers), *B* dsDECT (SOMATOM Force, Siemens Healthineers), *C* rsDECT (Revolution CT Apex, GE Healthcare), *D* dlDECT (Hawk spectral CT, Philips Healthcare), *E* ssDECT (Aquilion ONE, Canon Medical Systems)

### Inter-system reproducibility within the same dose level

**Fig. 4:**
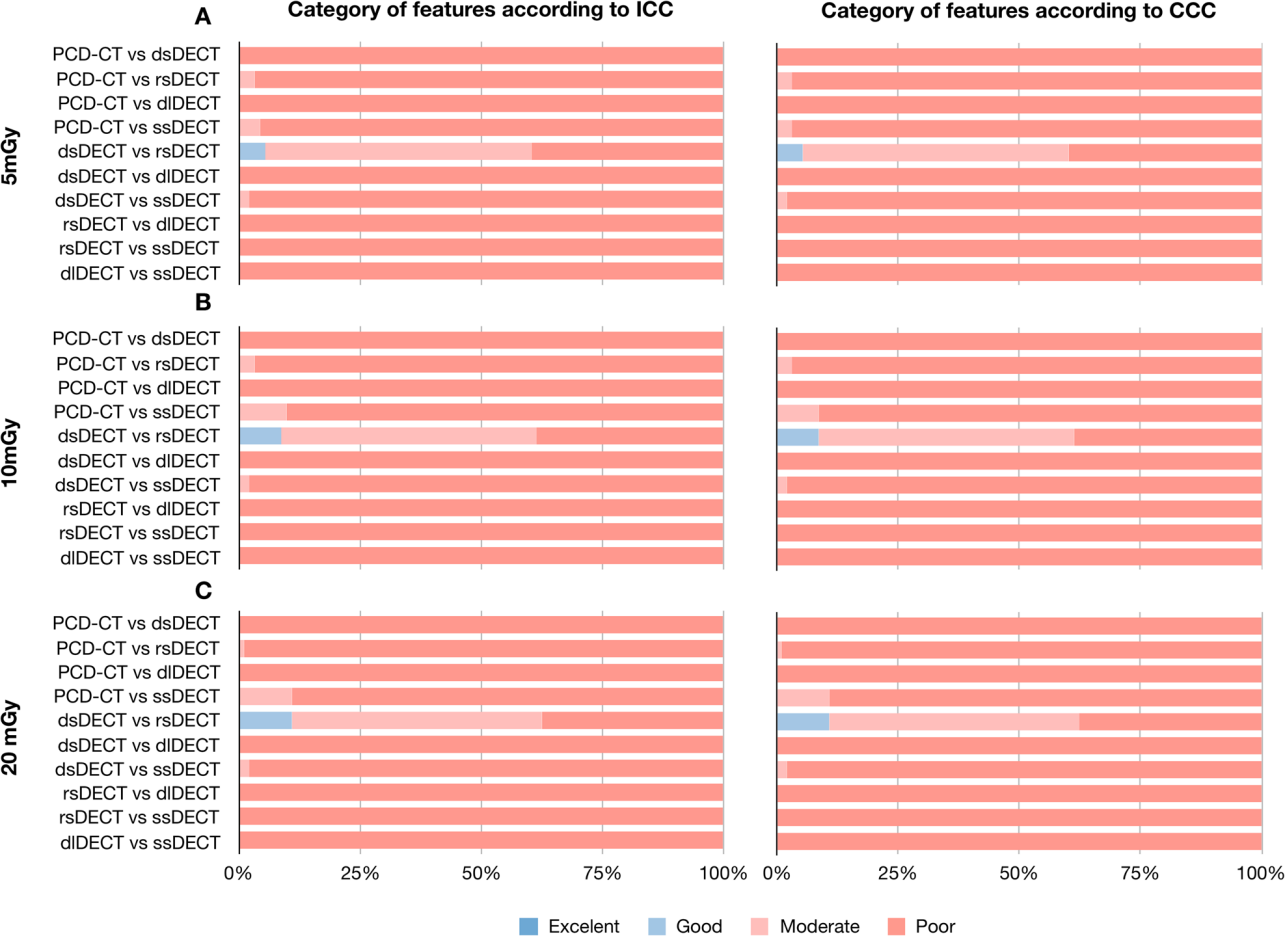
Percentage of robust radiomics features according to inter-system reproducibility. The percentage of robust features in terms of inter-system reproducibility among five scanners within the same dose level of (**A**) 5 mGy, (**B**) 10 mGy, and (**C**) 20 mGy, according to ICC and CCC values. The ICC and CCC values were interpreted as follows: poor, < 0.50; moderate, 0.50–0.75; good, 0.75–0.90; or excellent, ≥ 0.90

The overall mean ± standard deviation ICC and CCC values for inter-system reproducibility were 0.157 ± 0.174 and 0.157 ± 0.174, respectively (Table 3). None of the features were with ICC > 0.90 or CCC > 0.90, while 92.6% and 92.7% of features were with ICC < 0.50 and CCC < 0.50 (Fig. 4). There were only between dsDECT and rsDECT systems that showed 8.2% and 8.2% of features with ICC of 0.75–0.90 and CCC of 0.75–0.90, respectively. The results for each feature were summarized (Supplementary Fig. S3).

**Table 3:** Inter-system reproducibility within the same dose level

Scanner	5 mGy		10 mGy		20 mGy	
	ICC	CCC	ICC	CCC	ICC	CCC
A vs. B	0.083 ± 0.092	0.083 ± 0.091	0.096 ± 0.098	0.095 ± 0.098	0.103 ± 0.102	0.102 ± 0.101
A vs. C	0.108 ± 0.123	0.107 ± 0.123	0.114 ± 0.127	0.114 ± 0.127	0.122 ± 0.129	0.122 ± 0.129
A vs. D	0.138 ± 0.106	0.137 ± 0.105	0.145 ± 0.099	0.145 ± 0.098	0.148 ± 0.107	0.148 ± 0.106
A vs. E	0.193 ± 0.156	0.192 ± 0.155	0.210 ± 0.174	0.209 ± 0.174	0.239 ± 0.193	0.238 ± 0.193
B vs. C	0.505 ± 0.216	0.505 ± 0.215	0.516 ± 0.212	0.515 ± 0.212	0.523 ± 0.213	0.522 ± 0.213
B vs. D	0.080 ± 0.069	0.080 ± 0.069	0.085 ± 0.070	0.085 ± 0.070	0.095 ± 0.075	0.095 ± 0.075
B vs. E	0.094 ± 0.102	0.094 ± 0.101	0.107 ± 0.108	0.107 ± 0.108	0.102 ± 0.106	0.102 ± 0.106
C vs. D	0.080 ± 0.063	0.080 ± 0.063	0.087 ± 0.066	0.087 ± 0.066	0.097 ± 0.071	0.097 ± 0.071
C vs. E	0.088 ± 0.090	0.088 ± 0.090	0.097 ± 0.092	0.097 ± 0.091	0.097 ± 0.091	0.097 ± 0.090
D vs. E	0.120 ± 0.091	0.120 ± 0.090	0.116 ± 0.086	0.116 ± 0.085	0.124 ± 0.091	0.123 ± 0.091

The data were presented as mean ± standard deviation

*ICC* intraclass correlation coefficient, *CCC* concordance correlation coefficient. *A* PCD-CT system (NAEOTOM Alpha, Siemens Healthineers), *B* dsDECT (SOMATOM Force, Siemens Healthineers), *C* rsDECT (Revolution CT Apex, GE Healthcare), *D* dlDECT (Hawk spectral CT, Philips Healthcare), *E* ssDECT (Aquilion ONE, Canon Medical Systems)

### Inter-system variability among five scanners

**Fig. 5:**
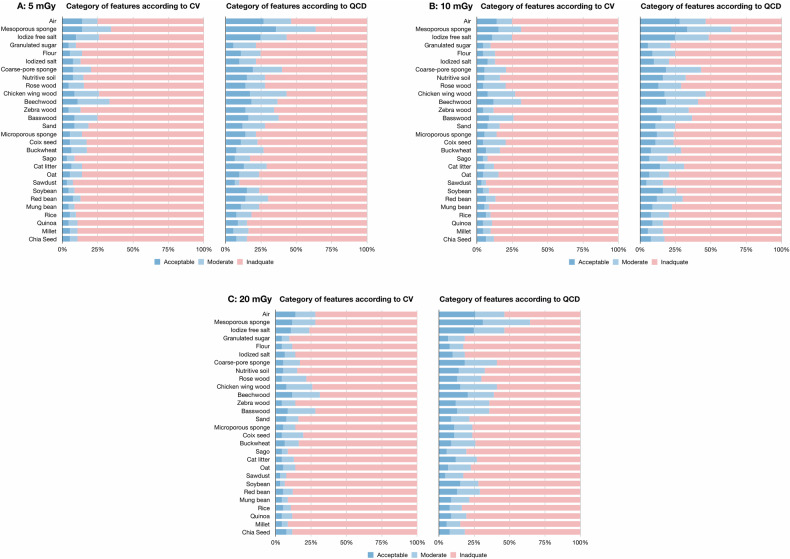
Percentage of robust radiomics features according to inter-system variability. The percentage of robust features in terms of inter-system variability among five scanners within the same dose level of **A** 5 mGy, **B** 10 mGy, and **C** 20 mGy, according to CV and QCD values. The CV and QCD values were interpreted as follows: acceptable, < 10%; moderate but still adequate, 11%–20%; and too high and inadequate, ≥ 20%

The overall mean ± standard deviation CV and QCD values for inter-system reproducibility were 88.8 ± 478.3% and 91.8 ± 2797.5%, respectively (Table 4 and Supplementary Fig. S4). The percentage of features with CV < 10% and QCD < 10% were 6.5% and 12.8%, respectively (Fig. 5). The inter-system reproducibility was heterogeneous among different materials, with mean ± standard deviation CV values ranged from 44.0 ± 42.1% to 437.6% ± 344.2%, and mean ± standard deviation QCD values from 25.6% ± 21.5% to 641.6% ± 182.2%, respectively. The percentage of features with CV < 10% and QCD < 10% ranged from 3.2% to 15.1%, and from 4.3% to 35.5%, respectively.

**Table 4:** Inter-system variability among five scanners according to materials

ROI	Material	5 mGy		10 mGy		20 mGy	
		CV	QCD	CV	QCD	CV	QCD
1	Air	84.8% ± 321.4%	38.6% ± 88.4%	89.8% ± 360.3%	42.0% ± 118.5%	96.0% ± 418.3%	641.6% ± 182.2%
2	Mesoporous sponge	65.3% ± 123.1%	28.6% ± 60.2%	69.8% ± 132.1%	28.4% ± 64.3%	77.4% ± 155.4%	28.1% ± 62.0%
3	Iodize free salt	65.3% ± 100.0%	25.6% ± 21.5%	67.4% ± 103.2%	25.2% ± 20.7%	72.0% ± 107.7%	25.5% ± 20.8%
4	Granulated sugar	92.2% ± 168.7%	48.0% ± 41.0%	98.3% ± 229.4%	48.4% ± 38.4%	112.4% ± 336.6%	49.8% ± 37.6%
5	Flour	68.1% ± 74.4%	47.5% ± 44.3%	68.1% ± 72.4%	49.0% ± 50.9%	66.3% ± 63.0%	52.8% ± 62.5%
6	Iodized salt	60.6% ± 41.6%	46.6% ± 41.2%	61.3% ± 42.7%	45.5% ± 33.4%	64.0% ± 44.7%	50.9% ± 62.7%
7	Coarse-pore sponge	143.2% ± 775.9%	39.5% ± 102.1%	143.8% ± 761.1%	48.5% ± 199.7%	106.1% ± 373.0%	32.0% ± 43.0%
8	Nutritive soil	93.9% ± 302.2%	89.7% ± 310.9%	113.7% ± 491.1%	491.5% ± 3988.7%	62.8% ± 56.3%	190.4% ± 1168.2%
9	Rosewood	71.7% ± 73.0%	76.7% ± 311.6%	70.8% ± 73.0%	61.3% ± 175.9%	70.6% ± 73.0%	72.3% ± 290.1%
10	Chicken wing wood	69.2% ± 144.0%	185.8% ± 1334.0%	67.7% ± 135.3%	77.9% ± 310.3%	70.5% ± 144.0%	68.0% ± 213.2%
11	Beechwood	44.0% ± 42.1%	30.9% ± 23.3%	44.5% ± 43.2%	29.9% ± 22.6%	45.1% ± 44.4%	29.8% ± 24.7%
12	Zebra wood	69.2% ± 61.1%	30.4% ± 24.5%	68.9% ± 60.3%	31.3% ± 24.7%	66.9% ± 57.4%	30.4% ± 22.4%
13	Basswood	44.9% ± 38.4%	33.7% ± 29.2%	45.3% ± 39.6%	34.2% ± 30.7%	45.2% ± 39.1%	34.4% ± 29.8%
14	Sand	51.7% ± 51.4%	44.2% ± 61.8%	53.0% ± 52.3%	45.0% ± 62.8%	55.9% ± 64.5%	48.2% ± 82.7%
15	Microporous sponge	78.2% ± 85.7%	55.5% ± 68.0%	77.9% ± 83.4%	151.2% ± 992.7%	78.9% ± 82.9%	62.7% ± 126.4%
16	Coix seed	80.1% ± 129.9%	54.9% ± 46.6%	81.0% ± 140.0%	55.0% ± 43.0%	78.2% ± 114.0%	55.0% ± 42.5%
17	Buckwheat	142.5% ± 558.4%	109.8% ± 595.2%	164.3% ± 756.4%	95.0% ± 449.7%	239.6% ± 1481%	67.3% ± 188.6%
18	Sago	103.1% ± 200.0%	46.3% ± 30.5%	103.8% ± 203.9%	46.1% ± 30.3%	102.0% ± 195.1%	46.3% ± 30.8%
19	Cat litter	53.5% ± 39.8%	37.4% ± 28.5%	55.0% ± 41.7%	37.4% ± 28.7%	60.2% ± 50.3%	39.5% ± 29.6%
20	Oat	437.6% ± 344.2%	43.5% ± 28.8%	155.6% ± 723.7%	44.7% ± 29.0%	124.5% ± 419.6%	46.0% ± 29.5%
21	Sawdust	106.3% ± 188.7%	60.4% ± 85.7%	105.7% ± 183.2%	59.3% ± 78.6%	104.7% ± 176.5%	58.2% ± 68.6%
22	Soybean	66.8% ± 40.6%	47.5% ± 31.8%	66.7% ± 38.2%	47.6% ± 32.2%	68.6% ± 38.6%	48.3% ± 32.4%
23	Red bean	63.5% ± 54.3%	47.1% ± 35.3%	64.2% ± 54.8%	48.6% ± 35.3%	64.7% ± 54.5%	49.3% ± 35.4%
24	Mung bean	99.8% ± 143%	57.9% ± 95.7%	98.3% ± 139.2%	56.6% ± 91.7%	97.7% ± 137.1%	57.8% ± 88.4%
25	Rice	129.3% ± 512.2%	54.2% ± 32.3%	115.1% ± 385.1%	53.3% ± 31.6%	121.5% ± 449.5%	53.6% ± 31.6%
26	Quinoa	92.6% ± 131.6%	51.6% ± 32.9%	95.1% ± 137.1%	51.3% ± 34.0%	94.7% ± 141.3%	50.8% ± 34.3%
27	Millet	79.8% ± 62.1%	57.4% ± 35.6%	80.3% ± 62.5%	57.3% ± 36.0%	79.8% ± 61.7%	56.8% ± 35.2%
28	Chia seed	82.6% ± 69.9%	58.9% ± 124.2%	84.7% ± 83.7%	194.8% ± 1428.7%	84.1% ± 84.3%	64.1% ± 165.0%

The data were presented as mean ± standard deviation

*CV* coefficient of variation, *QCD* quartile coefficient of dispersion

## Discussion

Our study showed that the repeatability of radiomics features was heterogeneous among CT techniques, in which PCD-CT, dsDECT, and rsDECT have relatively higher repeatability. On the other hand, the difference in radiation dose levels has less impact on the radiomics features. Notably, the radiomics features derived from images using different CT techniques were not reproducible to each other with significant variability in radiomics feature values, despite using carefully adjusted protocols.

The influence of the DECT technique has a great impact on the robustness of radiomics features. The phantom study showed that different DECT techniques led to the variability of radiomics features even though comparable parameters were used [30]. The deep learning image reconstruction algorithms cannot harmonize the variability of radiomics due to different DECT techniques [31]. However, the deep learning image reconstruction algorithms showed potential for minimizing radiomics variability that related to radiation dose level difference [33]. One opportunity for improving radiomics robustness across DECT systems is synchronizing energy levels of VMI to reach similar CT number values [32], while the potential influence on the diagnostic performance of the approach has not been estimated yet. Further studies in patients supported the phantom results that the radiomic features across different DECT systems were low, but the robust radiomics features were not reflected in the phantom experiment using the same parameters [29]. These studies compared dsDECT, rsDECT, and dlDECT systems, while our study further strengthened the results by including the ssDECT system. We used acquisition and reconstruction parameters as comparable as possible between the systems because we aimed to focus on the difference due that are specific to DECT systems. Therefore, the main sources of the low inter-system reproducibility were considered to be multi-energy acquisition or material decomposition techniques [29]. It is evident that they have a significant impact on the quantification of iodine [53–55]. Further, our study applied a phantom with materials of heterogeneous texture to validate the results. We summarized that the radiomics features were hard to be reproducible across the DECT systems whether the phantoms were homogeneous or heterogeneous, or were imitating physiological organ parenchyma [29, 41].

The image noise can be an important source of variability of radiomics features among CT systems. The studies showed that PCD-CT systems can provide high radiomics feature repeatability [37, 39], and high reproducibility between different radiation dose levels [34, 37], but heterogeneous reproducibility between VMIs at different keV levels [38]. Our study supported the high repeatability and high reproducibility of PCD-CT between different radiation dose levels. These results were in accordance with those in organic phantoms which the repeatability after repositioning and reproducibility between different tube currents were high [37]. It is reasonable since the PCD-CT has allowed for improved visualization and quantification even with ultra-low-dose imaging and obese patients [56], as it can remove the electronic background noise [57]. It is theoretically beneficial for radiomics analysis since this pixel-level image analysis approach is fragile to slight differences in images [25, 30]. In contrast, the repeatability in DECT systems can be suboptimal depending on the material decomposition technique [58, 59]. However, the dose level in our study was not that low to challenge some of the DECT systems in our study by electronic background noise. Therefore, the comparable high repeatability can be partially attributed to the relatively high radiation dose used in the study, in addition to the technique itself. Moreover, the same dose level does not guarantee the same signal-to-noise ratio as different DECT systems rely on very different technologies. However, it may be not possible to obtain the same signal-to-noise ratio among different CT systems with comparable acquisition parameters. Our study found that the first-order radiomics features have less variation than texture, which is in accordance with the previous phantom studies [30–33]. It is related to the fact that the texture features might be changed by image quality differences among CT systems. In addition, the pre-processing parameters also have a great impact on the radiomic analysis. Although the influence of these parameters is out of the scope of this work, we believe the selection of the bin size is especially important in our study. The selection of bin size can significantly influence on the image noise and thereby impact on the radiomics features in DECT systems [30]. However, the images from PCD-CT systems are less likely to be influenced by the bin size, since the PCD-CT systems are capable of removing the electronic background noise [57]. This should be investigated in future study to improve the reproducibility between different CT systems [60, 61].

It is believed that the radiomics features may be benefited from higher spatial resolution, higher contrast-to-noise ratio, and improved detection of lower-energy photons of PCD-CT system for better pathology characterization [34–36]. A phantom study of pulmonary nodules indicated that the estimation of morphological features may be improved in PCD-CT than in conventional CT systems [36] since the higher resolution in PCD-CT system allows better delineation of the nodule. Another organic phantom study showed a great difference of more than fifty percent were identified in 13 out of 14 selected radiomics features between PCD-CT and dsDECT systems [34]. On the other hand, a patient study compared radiomics features of non-scarred left ventricular myocardium suggested that first-order features were nearly comparable between PCD-CT and dsDECT systems, but texture features would be strongly changed [35]. Our study compared PCD-CT with four DECT systems and extended results that the radiomics features were not expected to be comparable between PCD-CT and DECT systems. We considered that the repeatability and reproducibility of radiomics features may not be substantially changed in some of the acquisition parameters such as radiation dose levels [25, 27, 34, 37]. In contrast, the acquisition and reconstruction parameters such as material decomposition technique, spatial resolution, and reconstruction kernels, have substantial impact on the radiomics feature values and result in a great decrease in the robustness of radiomics features [26, 27, 34, 35, 38, 39]. It is necessary to investigate the influence of reconstruction parameters on radiomics features within the PCT-CT system. However, it remains unknown whether the phantom experiments can reflect the reproducibility of radiomics features in clinical patient scans with various protocols. The agreement between phantom and patient experiments in the context of radiomics may be limited due to the difference in texture and the use of contrast media [38].

The following limitations of our study should be addressed. First, this was a phantom study without validation of human data. The texture of phantoms may differ from the physiological human parenchyma or pathological tissues [29, 41, 62]. Further, it is still unclear whether the low reproducibility will damage the presentation of the biological phenomenon in a clinical study. It may be not important if the change in radiomics features is significant enough between different biological phenomena. Therefore, the results may not be directly transferrable to patients. An improved organic phantom model with a specific disease would be preferable. However, our study gives an important insight into the variation of radiomics derived from images using heterogeneous CT techniques, as patient data of multiple scans on different CT systems are not always available [29]. Second, we did not include DECT systems using the split filter technique, and only one PCD-CT system was included in our study. These issues should be addressed in further studies to show whether the PCD-CT systems allow more stable radiomics features than traditional energy-integrating CT systems [34–36] and whether the radiomics analysis among PCD-CT systems is more generalizable. Third, we did not include traditional SECT systems in our study. This may reduce the translational value of our work. The DECT-like 70 keV VMI is recommended by the vendor for clinical use in abdominal scans instead of SECT-like low-energy-threshold polychromatic images [30–33]. Therefore, the 70 keV VMI from the PCD-CT was selected as the reference in our study. Accordingly, we chose the 70 keV VMI from DECT systems for comparison. In our future study, we will compare the SECT-like low-energy-threshold polychromatic images with the traditional SECT images in the terms of radiomics features. Fourth, the lowest radiation dose level in this study is relatively high. We did not test the stability of radiomics features at extra low radiation dose levels to present the advantage of PCD-CT systems providing stable quantification without disruption from electronic background noise [56]. It is expected that the PCD-CT systems allow reliable quantification within a wider range of radiation dose levels. Fifth, only VMI at 70 keV has been compared in this study. Both PCD-CT and DECT systems allow post-processing of material decomposition and linear blending to generate VMIs at different keV levels, iodine mappings, and virtual unenhanced images [38, 39]. The robustness of radiomics features derived from these images is also of interest because it is notable that different CT techniques has influence on the quantification of iodine [53–56]. Sixth, we applied bi-dimensional ROIs instead of three-dimensional ROIs. The selection has a potential impact on reproducibility. However, the influence of bi-dimensional or three-dimensional ROIs may be relatively small [63] and does not change the conclusion of the current study. Finally, this study did not evaluate the relationship between radiomics robustness and characterization ability. It should be considered in later clinical studies, as the radiomics analysis based on PCD-CT systems may change the clinical interpretation or classification in pathologies with rich textures or small volumes [34, 37].

To conclude, this study outlined the variability of radiomics features derived from VMIs generated using one PCD-CT system and four traditional energy-integrating DECT systems, despite using comparable protocols. Different radiation dose levels did not substantially change radiomics features, while the repeatability of radiomics features was heterogeneous across CT techniques. Radiomics analysis based on one CT technique should not be directly transferred to others without validation. Future investigations are encouraged to mitigate radiomics variability due to CT techniques.

## Supplementary information

ELECTRONIC SUPPLEMENTARY MATERIAL

## Abbreviations

CCC: Concordance correlation coefficient
CV: Coefficient of variation
DECT: Dual-energy computed tomography
dlDECT: Dual-layer dual-energy CT
dsDECT: Dual-source dual-energy CT
ICC: Intraclass correlation coefficient
PCD-CT: Photon-counting detector CT
QCD: Quartile coefficient of dispersion
ROI: Region of interest
rsDECT: Rapid kV-switching dual-energy CT
SECT: Single-energy CT
ssDECT: Sequential scanning DECT
VMI: Virtual monochromatic image
