# Supplementary material for: Robustness of radiomics among photon-counting detector CT and dual-energy CT systems: a texture phantom study
Source: Eur Radiol. 2024 Jul 24;35(2):871–84. doi: 10.1007/s00330-024-10976-1 (PMC11782343; doi:10.1007/s00330-024-10976-1)
Supplement: Supplementary file 1 — ELECTRONIC SUPPLEMENTARY MATERIAL [file 330_2024_10976_MOESM1_ESM.pdf]

Robustness of radiomics among photon-counting detector CT and dual-energy CT systems: a texture phantom study

ELECTRONIC SUPPLEMENTARY MATERIAL

List of Supplementary Materials

Supplementary Note S1 Details of the set-ups of texture phantom
Supplementary Note S2 Radiomics feature extraction method

Supplementary Table S1 Repeatability of radiomics features
Supplementary Table S2 Signal-to-noise ratio across the different datasets
Supplementary Table S3 Mean relative change of the radiomics features across the different datasets
Supplementary Table S4 The repeatability of radiomics features
Supplementary Table S5 The intra-system reproducibility among three dose levels
Supplementary Table S6 The inter-system reproducibility within the same dose level
Supplementary Table S7 The inter-system variability among five scanners according to materials

Supplementary Figure S1 Heatmap of repeatability of radiomics features
Supplementary Figure S2 Heatmap of intra-system reproducibility among three dose levels
Supplementary Figure S3 Heatmap of inter-system reproducibility within the same dose level
Supplementary Figure S4 Heatmap of inter-system variability among five scanners according to materials

Supplementary Note 1 Details of the set-ups of texture phantom

We established a texture phantom consisting of twenty-eight different materials. There were five wood blocks and twenty-three bottles filled with different materials.

(1) Wood blocks
The wood block was cuboid with a size of 150 mm × 55 mm × 45 mm. We bought and asked the seller to cut the wood into the size of 150 mm × 55 mm × 45 mm. The types of wood were selected to present different textures. We ask the seller to recommend the types of woods with heterogenous densities and textures. He kindly recommended the following five types of wood: rose wood, chicken wing wood, beechwood, zebra wood, and basswood.

(2) Bottles filled with different materials
We recycled the bottles of juice bought in the convenience store. Our colleagues drank the juice, and cleaned the bottles using fresh water. The bottles were then naturally dried for this study. They were also asked to provide various materials with heterogenous densities and textures.

The materials that they brought from their home or we bought from the supermarket or online includes following: mesoporous sponge, iodize free salt, granulated sugar, flour, iodized salt, coarse-pore sponge, nutritive soil for succulent plants, sand, microporous sponge, coix seed, buckwheat, sago, cat litter, oat, sawdust, soybean, red bean, mung bean, rice, quinoa, millet, and chia seed. We additionally included a bottle filled with air.

The cuboid part of the bottle was with a size of 130 mm × 55 mm × 45 mm. The cuboid part bottle was filled with materials as tightly as possible, and tissue was put into the cylindrical part of the bottle if needed to avoid unexpected movement. These materials were selected to give us varying texture. The materials were positioned to avoid beam-hardening artifacts and were kept unchanged throughout all the scans in the study.

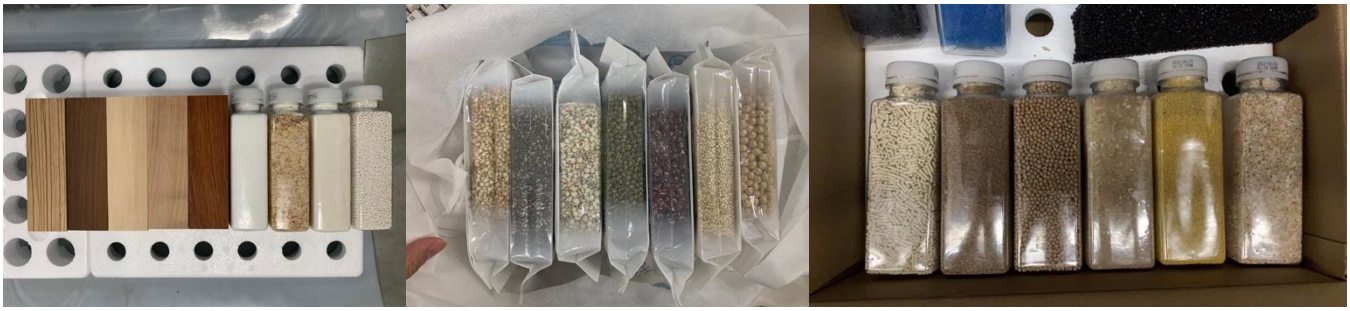

Supplementary Note 2 Radiomics feature extraction method

(1) yaml document for radiomics feature extraction

We did not perform any post-processing steps (such as resampling, normalization, combat, etc.) before the feature extraction.

```
featureClass:
  firstorder: null
  glcm: null
  gldm: null
  glrlm: null
  glszm: null
  ngtdm: null
imageType:
  Original: {}
setting:
  additionalInfo: false
  binCount: 16
  force2D: true
  geometryTolerance: 1e-6
  label: 1
  resegmentMode: sigma
```

(2) Name of calculated features

The details of calculation of each feature are available via the website of PyRadiomics (https://pyradiomics.readthedocs.io/en/latest/).

No	Feature family	Name of features
1	firstorder	10Percentile
2	firstorder	90Percentile
3	firstorder	Energy
4	firstorder	Entropy
5	firstorder	InterquartileRange
6	firstorder	Kurtosis
7	firstorder	Maximum
8	firstorder	MeanAbsoluteDeviation
9	firstorder	Mean
10	firstorder	Median
11	firstorder	Minimum
12	firstorder	Range
13	firstorder	RobustMeanAbsoluteDeviation
14	firstorder	RootMeanSquared
15	firstorder	Skewness
16	firstorder	TotalEnergy
17	firstorder	Uniformity
18	firstorder	Variance
19	glcm	Autocorrelation

20	glcm	ClusterProminence
21	glcm	ClusterShade
22	glcm	ClusterTendency
23	glcm	Contrast
24	glcm	Correlation
25	glcm	DifferenceAverage
26	glcm	DifferenceEntropy
27	glcm	DifferenceVariance
28	glcm	Id
29	glcm	Idm
30	glcm	Idmn
31	glcm	Idn
32	glcm	Imc1
33	glcm	Imc2
34	glcm	InverseVariance
35	glcm	JointAverage
36	glcm	JointEnergy
37	glcm	JointEntropy
38	glcm	MCC
39	glcm	MaximumProbability
40	glcm	SumAverage
41	glcm	SumEntropy
42	glcm	SumSquares
43	gldm	DependenceEntropy
44	gldm	DependenceNonUniformity
45	gldm	DependenceNonUniformityNormalized
46	gldm	DependenceVariance
47	gldm	GrayLevelNonUniformity
48	gldm	GrayLevelVariance
49	gldm	HighGrayLevelEmphasis
50	gldm	LargeDependenceEmphasis
51	gldm	LargeDependenceHighGrayLevelEmphasis
52	gldm	LargeDependenceLowGrayLevelEmphasis
53	gldm	LowGrayLevelEmphasis
54	gldm	SmallDependenceEmphasis
55	gldm	SmallDependenceHighGrayLevelEmphasis
56	gldm	SmallDependenceLowGrayLevelEmphasis
57	glrlm	GrayLevelNonUniformity
58	glrlm	GrayLevelNonUniformityNormalized
59	glrlm	GrayLevelVariance
60	glrlm	HighGrayLevelRunEmphasis
61	glrlm	LongRunEmphasis
62	glrlm	LongRunHighGrayLevelEmphasis
63	glrlm	LongRunLowGrayLevelEmphasis

Eur Radiol (2024) Zhu L, Dong H, Sun J, et al.

64	glrlm	LowGrayLevelRunEmphasis
65	glrlm	RunEntropy
66	glrlm	RunLengthNonUniformity
67	glrlm	RunLengthNonUniformityNormalized
68	glrlm	RunPercentage
69	glrlm	RunVariance
70	glrlm	ShortRunEmphasis
71	glrlm	ShortRunHighGrayLevelEmphasis
72	glrlm	ShortRunLowGrayLevelEmphasis
73	glszm	GrayLevelNonUniformity
74	glszm	GrayLevelNonUniformityNormalized
75	glszm	GrayLevelVariance
76	glszm	HighGrayLevelZoneEmphasis
77	glszm	LargeAreaEmphasis
78	glszm	LargeAreaHighGrayLevelEmphasis
79	glszm	LargeAreaLowGrayLevelEmphasis
80	glszm	LowGrayLevelZoneEmphasis
81	glszm	SizeZoneNonUniformity
82	glszm	SizeZoneNonUniformityNormalized
83	glszm	SmallAreaEmphasis
84	glszm	SmallAreaHighGrayLevelEmphasis
85	glszm	SmallAreaLowGrayLevelEmphasis
86	glszm	ZoneEntropy
87	glszm	ZonePercentage
88	glszm	ZoneVariance
89	ngtdm	Busyness
90	ngtdm	Coarseness
91	ngtdm	Complexity
92	ngtdm	Contrast
93	ngtdm	Strength

Supplementary Table 1 Repeatability of radiomics features

	B-A > 95%	B-A > 90%	B-A > 85%
5 mGy			
PCD-CT	68.8%	100.0%	100.0%
dsDECT	44.1%	100.0%	100.0%
rsDECT	12.9%	100.0%	100.0%
dlDECT	3.2%	82.8%	100.0%
ssDECT	39.8%	100.0%	100.0%
10 mGy			
PCD-CT	57.0%	100.0%	100.0%
dsDECT	43.0%	100.0%	100.0%
rsDECT	21.5%	100.0%	100.0%
dlDECT	7.5%	84.9%	100.0%
ssDECT	25.8%	100.0%	100.0%
20 mGy			
PCD-CT	60.2%	100.0%	100.0%
dsDECT	34.4%	100.0%	100.0%
rsDECT	40.9%	100.0%	100.0%
dlDECT	9.7%	88.2%	100.0%
ssDECT	26.9%	100.0%	100.0%
Overall	33.0 ± 20.1%	97.1 ± 6.2%	100.0 ± 0.0%

Supplementary Table S2 Signal-to-noise ratio across the different datasets

ROI	5 mGy					10 mGy					20 mGy				
	PCD-CT	dsDECT	rsDECT	dlDECT	ssDECT	PCD-CT	dsDECT	rsDECT	dlDECT	ssDECT	PCD-CT	dsDECT	rsDECT	dlDECT	ssDECT
1	0.0024	0.0031	0.0032	1.0402	0.0018	0.0024	0.0031	0.0031	1.0835	0.0018	0.0024	0.0030	0.0031	1.0923	0.0016
2	0.0017	0.0037	0.0045	0.9692	0.8539	0.0016	0.0035	0.0043	1.0184	1.1627	0.0016	0.0034	0.0043	1.0234	0.9341
3	0.0024	0.0057	0.0072	1.1227	0.0014	0.0024	0.0054	0.0071	1.1113	0.0014	0.0023	0.0052	0.0069	1.1706	0.0011
4	0.0075	0.0062	0.0178	0.9034	0.0017	0.0074	0.0061	0.0173	0.9300	0.0017	0.0074	0.0061	0.0172	0.9531	0.0013
5	1.0338	0.0137	0.0135	0.9588	0.0071	1.1576	0.0129	0.0134	0.9589	0.0070	1.2377	0.0124	0.0133	0.9824	0.0047
6	0.1406	0.0065	0.0156	1.2392	0.1294	0.1476	0.0064	0.0154	1.2857	0.1536	0.1503	0.0063	0.0154	1.3389	0.1044
7	0.0875	0.0120	0.0132	1.0764	0.0986	0.0847	0.0112	0.0126	1.1085	0.1045	0.0857	0.0107	0.0122	1.1241	0.0683
8	10.3329	0.0011	0.0014	0.0051	0.5634	9.7177	0.0011	0.0014	0.0047	0.5839	9.2621	0.0010	0.0014	0.0047	0.5037
9	0.0053	0.0024	0.0206	0.0036	8.6758	0.0053	0.0024	0.0199	0.0035	13.8023	0.0052	0.0024	0.0196	0.0033	17.4690
10	0.5056	0.0182	0.0371	4.1772	0.0040	0.5139	0.0173	0.0365	4.2183	0.0039	0.5148	0.0168	0.0361	5.3562	0.0029
11	0.0137	0.0279	0.0492	0.0052	0.0110	0.0135	0.0271	0.0481	0.0054	0.0113	0.0134	0.0264	0.0473	0.0049	0.0074
12	0.0320	0.0341	0.0014	0.0240	0.0299	0.0309	0.0329	0.0013	0.0222	0.0300	0.0304	0.0319	0.0013	0.0202	0.0190
13	0.1190	0.0218	0.0754	0.5769	0.0652	0.1192	0.0217	0.0751	0.6352	0.0648	0.1210	0.0212	0.0743	0.6542	0.0527
14	0.0074	0.0740	0.1323	0.4575	0.0057	0.0073	0.0717	0.1334	0.4626	0.0056	0.0073	0.0709	0.1330	0.4597	0.0041
15	0.0124	0.0008	0.0005	11.4819	0.0120	0.0123	0.0008	0.0005	19.1870	0.0160	0.0126	0.0008	0.0005	25.1650	0.0067
16	0.5239	0.0065	0.0065	0.0143	0.0095	0.5062	0.0065	0.0063	0.0126	0.0097	0.4783	0.0065	0.0062	0.0128	0.0069
17	0.2096	0.0026	0.0013	0.8733	0.4470	0.2065	0.0026	0.0013	0.9193	0.5152	0.2013	0.0026	0.0013	0.9100	0.5302
18	0.1055	0.0012	0.0137	0.0459	0.5418	0.1041	0.0012	0.0133	0.0366	0.6442	0.1020	0.0012	0.0124	0.0354	0.5241
19	0.2846	0.0124	0.0403	0.1218	0.2167	0.2907	0.0123	0.0386	0.0946	0.2254	0.2832	0.0121	0.0371	0.0953	0.1700
20	0.4237	0.0236	0.0032	0.0180	0.5769	0.4352	0.0234	0.0031	0.0176	0.6713	0.4406	0.0227	0.0030	0.0170	0.5511
21	0.0100	0.0009	0.0081	0.2068	0.5084	0.0079	0.0009	0.0079	0.2083	0.5647	0.0088	0.0009	0.0078	0.2038	0.5481
22	2.9861	0.0913	0.0866	0.0501	8.5477	3.2200	0.0894	0.0849	0.0426	14.5829	3.3636	0.0868	0.0823	0.0439	14.6275
23	5.4877	0.1278	0.1008	1.0494	1.4035	5.8023	0.1223	0.0993	1.2343	1.7539	5.7959	0.1227	0.0981	1.2494	1.4434
24	1.4569	0.0992	0.0006	0.0320	4.4041	1.7114	0.0970	0.0005	0.0393	4.6474	1.8746	0.0942	0.0005	0.0323	4.4584
25	0.1714	0.0003	0.0078	0.6156	5.5724	0.1920	0.0003	0.0078	0.6808	8.0242	0.2036	0.0003	0.0080	0.6173	8.3479
26	0.5670	0.0053	0.0122	0.3741	1.2132	0.7172	0.0053	0.0122	0.3216	1.3482	0.7924	0.0053	0.0124	0.3181	1.3871
27	1.4403	0.0072	0.0021	0.6947	0.1510	1.7518	0.0071	0.0021	0.6799	0.1640	1.7406	0.0070	0.0021	0.6497	0.1475
28	4.8743	0.0005	0.1622	0.4934	0.3660	7.8860	0.0005	0.1541	0.5261	0.4066	7.5586	0.0005	0.1436	0.5153	0.4153

Supplementary Table S3 Mean relative change of the radiomics features across the different datasets

ROI	5 mGy					10 mGy					20 mGy				
	PCD-CT	dsDECT	rsDECT	dlDECT	ssDECT	PCD-CT	dsDECT	rsDECT	dlDECT	ssDECT	PCD-CT	dsDECT	rsDECT	dlDECT	ssDECT
1	n. a.	-9.7%	-0.2%	-26.0%	-38.3%	n. a.	-4.7%	-0.2%	-26.5%	-37.8%	n. a.	-3.2%	-0.1%	-24.0%	-35.4%
2	n. a.	-14.4%	0.2%	-0.1%	0.0%	n. a.	-6.8%	0.1%	-0.1%	0.0%	n. a.	-4.3%	0.2%	-0.1%	0.0%
3	n. a.	-22.2%	-0.3%	-0.9%	-0.3%	n. a.	-7.7%	-0.3%	-0.9%	-0.3%	n. a.	-8.4%	-0.2%	-1.2%	-0.3%
4	n. a.	-43.4%	-0.5%	-4.4%	-46.5%	n. a.	-21.0%	-0.3%	-4.9%	-41.3%	n. a.	-10.7%	-0.3%	-5.5%	-51.0%
5	n. a.	-16.0%	-1.8%	-50.7%	-1.8%	n. a.	-8.3%	-1.2%	-53.0%	-1.6%	n. a.	-4.7%	-1.1%	-40.4%	-1.8%
6	n. a.	-38.3%	-0.1%	-6.9%	-9.3%	n. a.	-22.0%	-0.1%	-6.5%	-8.1%	n. a.	-17.5%	-0.1%	-7.0%	-7.7%
7	n. a.	-79.0%	-0.1%	-0.6%	-1.3%	n. a.	-33.9%	-0.1%	-0.6%	-1.2%	n. a.	-18.5%	-0.1%	-0.7%	-1.2%
8	n. a.	-3.6%	-0.2%	-24.2%	-26.0%	n. a.	-3.5%	-0.1%	-25.0%	-26.9%	n. a.	-3.9%	-0.1%	-24.6%	-27.7%
9	n. a.	-0.1%	-0.1%	-9.9%	-31.6%	n. a.	-0.1%	-0.2%	-11.2%	-31.8%	n. a.	-0.1%	-0.1%	-10.8%	-37.4%
10	n. a.	0.1%	-0.5%	-1.6%	-2.4%	n. a.	0.1%	-0.4%	-1.5%	-2.2%	n. a.	0.1%	-0.4%	-1.2%	-1.9%
11	n. a.	0.0%	-0.1%	-0.9%	-6.0%	n. a.	0.0%	-0.1%	-0.9%	-5.6%	n. a.	0.0%	0.0%	-1.2%	-5.6%
12	n. a.	1.4%	0.2%	-64.4%	-1.4%	n. a.	-6.2%	1.0%	-83.0%	2.2%	n. a.	0.3%	-0.1%	-71.4%	-1.0%
13	n. a.	0.1%	-0.1%	-4.3%	-2.8%	n. a.	0.1%	-0.1%	-4.6%	-2.5%	n. a.	0.1%	-0.1%	-3.8%	-2.7%
14	n. a.	0.1%	-0.2%	-16.1%	-6.7%	n. a.	0.1%	-0.1%	-15.3%	-6.7%	n. a.	0.2%	-0.1%	-12.7%	-5.7%
15	n. a.	0.1%	-105.1%	-56.0%	-88.6%	n. a.	0.1%	-72.7%	-50.0%	-83.9%	n. a.	0.1%	-61.7%	-51.9%	-89.0%
16	n. a.	-0.3%	-1.1%	-218.2%	-216.4%	n. a.	-0.3%	-0.8%	-171.6%	-178.7%	n. a.	-0.3%	-0.8%	-114.6%	-167.4%
17	n. a.	-0.2%	0.1%	-39.2%	-63.9%	n. a.	-0.2%	0.1%	-30.0%	-58.2%	n. a.	-0.2%	0.1%	-35.8%	-64.6%
18	n. a.	0.2%	-0.2%	-49.1%	-161.8%	n. a.	0.2%	-0.3%	-55.3%	-140.7%	n. a.	0.2%	-0.2%	-39.4%	-140.9%
19	n. a.	0.1%	0.0%	-25.9%	-7.2%	n. a.	0.1%	0.0%	-34.4%	-7.2%	n. a.	0.1%	0.0%	-19.6%	-5.3%
20	n. a.	0.0%	0.1%	-35.3%	-6.2%	n. a.	0.0%	0.1%	-39.0%	-5.9%	n. a.	0.0%	0.1%	-44.4%	-5.9%
21	n. a.	-6.5%	-5.7%	-30.1%	-29.9%	n. a.	-6.4%	-5.6%	-30.8%	-31.0%	n. a.	-6.4%	-5.4%	-28.6%	-27.6%
22	n. a.	-1.5%	-1.0%	-1.3%	-1.5%	n. a.	-1.0%	-0.6%	-1.7%	-1.9%	n. a.	-0.9%	-0.6%	-1.4%	-1.7%
23	n. a.	0.1%	0.1%	-35.1%	-21.4%	n. a.	0.1%	0.1%	-46.8%	-29.2%	n. a.	0.1%	0.1%	-36.3%	-25.2%
24	n. a.	-2.9%	0.2%	-217.1%	-56.4%	n. a.	-3.3%	0.1%	-266.7%	-58.5%	n. a.	-3.0%	0.2%	-372.9%	-74.4%
25	n. a.	-1.5%	-5.2%	-73.7%	-72.0%	n. a.	-0.5%	-4.7%	-129.6%	-117.6%	n. a.	-0.6%	-3.9%	-100.6%	-102.6%
26	n. a.	-0.4%	-3.3%	-281.3%	-131.1%	n. a.	-0.2%	-1.3%	-395.0%	-189.4%	n. a.	-0.2%	-0.9%	-469.7%	-262.9%
27	n. a.	0.0%	-0.2%	-754.9%	-307.7%	n. a.	0.0%	-0.4%	-964.2%	-386.6%	n. a.	-0.1%	-0.5%	-1151.4%	-482.3%
28	n. a.	-0.2%	-0.2%	-81.5%	-444.0%	n. a.	-0.2%	-0.4%	-75.4%	-474.8%	n. a.	-0.2%	-0.3%	-93.0%	-570.3%

Supplementary Table S4 The repeatability of radiomics features

Feature type	A1	A2	A3	B1	B2	B3	C1	C2	C3	D1	D2	D3	E1	E2	E3
ICC															
First order	0.990	0.996	0.998	0.998	0.999	0.999	0.998	0.998	0.998	0.715	0.724	0.733	0.852	0.862	0.857
GLCM	0.939	0.969	0.985	0.994	0.994	0.995	0.993	0.993	0.992	0.652	0.679	0.712	0.769	0.801	0.807
GLDM	0.889	0.932	0.966	0.987	0.989	0.990	0.988	0.987	0.986	0.639	0.670	0.705	0.603	0.655	0.648
GLRLM	0.781	0.872	0.975	0.984	0.984	0.985	0.985	0.979	0.980	0.616	0.657	0.701	0.531	0.595	0.572
GLSZM	0.855	0.911	0.941	0.931	0.937	0.934	0.920	0.918	0.934	0.556	0.576	0.643	0.504	0.564	0.546
NDTDM	0.903	0.955	0.974	0.982	0.987	0.985	0.987	0.981	0.979	0.573	0.563	0.601	0.726	0.787	0.766
CCC															
First order	0.990	0.996	0.998	0.998	0.999	0.999	0.998	0.998	0.998	0.714	0.723	0.732	0.851	0.862	0.857
GLCM	0.938	0.968	0.985	0.994	0.994	0.995	0.993	0.993	0.992	0.651	0.678	0.711	0.768	0.800	0.806
GLDM	0.888	0.931	0.966	0.987	0.989	0.989	0.988	0.987	0.986	0.637	0.669	0.704	0.602	0.654	0.647
GLRLM	0.780	0.871	0.975	0.984	0.984	0.985	0.985	0.979	0.980	0.614	0.656	0.700	0.529	0.594	0.571
GLSZM	0.854	0.910	0.941	0.931	0.936	0.934	0.919	0.918	0.933	0.555	0.575	0.642	0.503	0.564	0.545
NDTDM	0.902	0.955	0.974	0.982	0.987	0.985	0.987	0.981	0.979	0.571	0.561	0.599	0.725	0.786	0.765

Supplementary Table S5 The intra-system reproducibility among three dose levels

Feature type	A1/A2	A1/A3	A2/A3	B1/B2	B1/B3	B2/B3	C1/C2	C1/C3	C2/C3	D1/D2	D1/D3	D2/D3	E1/E2	E1/E3	E2/E3
ICC															
First order	0.996	0.993	0.996	0.998	0.995	0.999	0.998	0.996	0.998	0.992	0.989	0.995	0.992	0.983	0.986
GLCM	0.950	0.934	0.970	0.993	0.987	0.995	0.992	0.987	0.994	0.959	0.931	0.962	0.930	0.931	0.936
GLDM	0.912	0.892	0.935	0.986	0.974	0.990	0.983	0.970	0.989	0.932	0.887	0.933	0.893	0.898	0.924
GLRLM	0.876	0.764	0.846	0.983	0.965	0.983	0.974	0.959	0.984	0.934	0.859	0.921	0.908	0.911	0.931
GLSZM	0.895	0.839	0.889	0.932	0.918	0.941	0.911	0.891	0.931	0.883	0.829	0.906	0.876	0.870	0.901
NDTDM	0.925	0.838	0.920	0.984	0.974	0.986	0.979	0.966	0.983	0.935	0.912	0.945	0.908	0.905	0.901
CCC															
First order	0.996	0.993	0.996	0.998	0.995	0.999	0.998	0.996	0.998	0.992	0.989	0.995	0.992	0.983	0.986
GLCM	0.950	0.934	0.970	0.993	0.986	0.995	0.992	0.987	0.994	0.959	0.931	0.962	0.930	0.931	0.936
GLDM	0.912	0.891	0.935	0.986	0.974	0.990	0.983	0.970	0.989	0.932	0.886	0.933	0.893	0.898	0.924
GLRLM	0.876	0.763	0.846	0.983	0.965	0.983	0.974	0.959	0.983	0.934	0.858	0.921	0.908	0.911	0.931
GLSZM	0.895	0.839	0.889	0.932	0.918	0.940	0.911	0.891	0.931	0.882	0.829	0.906	0.876	0.870	0.901
NDTDM	0.925	0.838	0.920	0.984	0.974	0.986	0.979	0.966	0.983	0.934	0.912	0.945	0.908	0.905	0.901

Supplementary Table S6 The inter-system reproducibility within the same dose level

Feature type	ICC						CCC					
	First order	GLCM	GLDM	GLRLM	GLSZM	NDTDM	First order	GLCM	GLDM	GLRLM	GLSZM	NDTDM
A1/B1	0.126	0.086	0.092	0.058	0.065	0.025	0.126	0.086	0.092	0.058	0.065	0.025
A2/B2	0.125	0.099	0.117	0.067	0.082	0.052	0.124	0.099	0.117	0.067	0.082	0.051
A3/B3	0.126	0.109	0.127	0.072	0.091	0.054	0.126	0.108	0.126	0.072	0.091	0.054
A1/C1	0.169	0.098	0.117	0.066	0.104	0.053	0.169	0.098	0.116	0.066	0.103	0.053
A2/C2	0.168	0.103	0.14	0.064	0.111	0.071	0.167	0.103	0.14	0.063	0.111	0.071
A3/C3	0.167	0.112	0.152	0.072	0.126	0.075	0.167	0.112	0.151	0.071	0.125	0.075
A1/D1	0.194	0.136	0.133	0.134	0.091	0.118	0.193	0.135	0.132	0.134	0.091	0.118
A2/D2	0.195	0.148	0.143	0.146	0.093	0.123	0.194	0.147	0.143	0.145	0.093	0.123
A3/D3	0.196	0.165	0.137	0.139	0.1	0.109	0.195	0.164	0.136	0.139	0.1	0.108
A1/E1	0.224	0.28	0.158	0.102	0.154	0.172	0.224	0.279	0.157	0.102	0.154	0.172
A2/E2	0.231	0.305	0.161	0.11	0.185	0.211	0.23	0.304	0.161	0.11	0.185	0.211
A3/E3	0.211	0.355	0.215	0.128	0.216	0.265	0.211	0.355	0.215	0.128	0.216	0.264
B1/C1	0.369	0.619	0.612	0.495	0.413	0.479	0.368	0.618	0.611	0.495	0.412	0.478
B2/C2	0.37	0.63	0.627	0.507	0.432	0.479	0.37	0.629	0.626	0.506	0.431	0.478
B3/C3	0.371	0.638	0.638	0.522	0.432	0.487	0.371	0.637	0.637	0.521	0.432	0.486
B1/D1	0.121	0.076	0.063	0.066	0.077	0.059	0.12	0.075	0.063	0.065	0.077	0.059
B2/D2	0.12	0.087	0.075	0.074	0.074	0.059	0.119	0.086	0.075	0.074	0.074	0.059
B3/D3	0.121	0.101	0.09	0.09	0.075	0.063	0.12	0.1	0.09	0.09	0.075	0.063
B1/E1	0.164	0.089	0.072	0.056	0.089	0.069	0.163	0.088	0.072	0.055	0.089	0.069
B2/E2	0.164	0.112	0.093	0.067	0.09	0.103	0.163	0.112	0.092	0.067	0.089	0.102
B3/E3	0.17	0.103	0.097	0.061	0.078	0.076	0.17	0.102	0.096	0.061	0.078	0.076
C1/D1	0.117	0.08	0.066	0.068	0.076	0.042	0.116	0.079	0.066	0.068	0.076	0.041
C2/D2	0.114	0.091	0.08	0.075	0.083	0.042	0.113	0.091	0.08	0.074	0.082	0.042
C3/D3	0.116	0.104	0.095	0.091	0.092	0.044	0.115	0.103	0.095	0.09	0.091	0.044
C1/E1	0.144	0.077	0.089	0.053	0.081	0.077	0.144	0.077	0.089	0.053	0.08	0.077
C2/E2	0.145	0.091	0.105	0.062	0.08	0.107	0.144	0.09	0.104	0.061	0.08	0.107

C3/E3	0.148	0.088	0.106	0.059	0.087	0.091	0.148	0.087	0.106	0.059	0.087	0.091
D1/E1	0.151	0.124	0.102	0.122	0.087	0.147	0.15	0.123	0.101	0.121	0.086	0.147
D2/E2	0.158	0.115	0.104	0.119	0.077	0.127	0.157	0.115	0.103	0.118	0.076	0.126
D3/E3	0.156	0.139	0.098	0.12	0.088	0.131	0.156	0.139	0.097	0.119	0.088	0.131

Supplementary Table S7 The inter-system variability among five scanners according to materials

ROI	CV						QCD					
	First order	GLCM	GLDM	GLRLM	GLSZM	NDTDM	First order	GLCM	GLDM	GLRLM	GLSZM	NDTDM
5 mGy												
1	269%	42%	44%	38%	42%	27%	42%	56%	36%	27%	28%	21%
2	154%	41%	41%	38%	63%	25%	77%	15%	19%	14%	22%	15%
3	136%	46%	49%	42%	65%	30%	31%	20%	26%	19%	36%	22%
4	198%	63%	65%	72%	74%	53%	66%	41%	47%	41%	53%	31%
5	112%	48%	58%	62%	74%	39%	81%	35%	43%	38%	50%	23%
6	98%	37%	60%	54%	65%	44%	84%	33%	42%	33%	47%	34%
7	503%	52%	58%	52%	73%	39%	89%	22%	30%	24%	37%	28%
8	255%	47%	54%	53%	70%	55%	290%	39%	45%	42%	42%	42%
9	123%	54%	63%	61%	65%	52%	236%	37%	41%	37%	41%	35%
10	152%	65%	44%	38%	48%	29%	806%	44%	38%	29%	37%	23%
11	49%	38%	44%	37%	58%	33%	31%	31%	33%	22%	38%	30%
12	67%	60%	69%	76%	84%	56%	34%	31%	28%	21%	37%	29%
13	64%	37%	42%	36%	52%	27%	39%	28%	37%	30%	40%	21%
14	83%	33%	48%	44%	60%	38%	77%	27%	42%	36%	47%	35%
15	160%	52%	54%	53%	80%	54%	86%	60%	44%	38%	44%	47%
16	177%	49%	61%	63%	61%	49%	83%	42%	52%	51%	53%	42%
17	463%	63%	72%	67%	65%	57%	385%	39%	44%	47%	47%	48%
18	261%	59%	65%	69%	68%	76%	49%	42%	49%	42%	49%	54%
19	57%	43%	55%	55%	64%	50%	34%	33%	43%	37%	44%	40%
20	25%	52%	61%	70%	68%	65%	55%	34%	43%	43%	46%	43%
21	223%	78%	80%	81%	77%	66%	117%	46%	46%	47%	51%	40%
22	78%	52%	68%	70%	76%	51%	35%	46%	60%	53%	50%	39%
23	114%	41%	57%	56%	60%	43%	68%	33%	50%	48%	46%	30%
24	221%	58%	73%	82%	74%	75%	116%	36%	49%	50%	48%	33%
25	413%	52%	67%	69%	64%	56%	66%	45%	57%	57%	51%	45%

26	227%	53%	64%	66%	65%	52%	68%	42%	52%	53%	50%	42%
27	151%	55%	67%	67%	65%	67%	82%	45%	55%	57%	53%	54%
28	134%	56%	69%	84%	79%	71%	118%	35%	50%	52%	48%	39%
10 mGy												
1	289%	46%	44%	38%	45%	26%	43%	69%	36%	27%	28%	21%
2	156%	51%	45%	42%	66%	26%	80%	15%	17%	13%	20%	14%
3	138%	47%	51%	45%	65%	31%	30%	20%	25%	19%	35%	21%
4	241%	60%	65%	69%	69%	52%	64%	41%	48%	42%	54%	32%
5	111%	47%	58%	63%	73%	39%	86%	35%	44%	40%	50%	23%
6	100%	37%	62%	54%	67%	44%	77%	33%	41%	34%	48%	33%
7	497%	52%	61%	56%	77%	41%	142%	21%	29%	22%	36%	27%
8	358%	47%	54%	53%	71%	52%	2373%	38%	45%	40%	39%	39%
9	124%	52%	62%	59%	65%	49%	163%	35%	40%	36%	41%	33%
10	145%	64%	44%	37%	48%	30%	237%	55%	37%	28%	37%	23%
11	49%	38%	45%	38%	60%	32%	30%	31%	32%	22%	36%	30%
12	66%	59%	68%	78%	82%	56%	35%	32%	29%	23%	37%	30%
13	64%	37%	42%	36%	54%	27%	40%	30%	37%	30%	39%	23%
14	84%	34%	49%	47%	61%	39%	77%	27%	42%	38%	48%	36%
15	158%	52%	54%	54%	78%	55%	81%	436%	43%	38%	43%	47%
16	183%	50%	61%	63%	60%	48%	79%	44%	52%	53%	53%	43%
17	575%	62%	73%	68%	66%	59%	309%	39%	43%	47%	48%	47%
18	264%	59%	65%	69%	69%	78%	50%	42%	49%	40%	49%	54%
19	57%	44%	57%	58%	66%	51%	35%	33%	41%	37%	43%	42%
20	546%	52%	61%	69%	68%	67%	57%	35%	44%	45%	47%	45%
21	221%	78%	81%	79%	77%	68%	113%	46%	45%	46%	50%	40%
22	80%	53%	72%	69%	71%	51%	36%	46%	59%	53%	50%	39%
23	115%	42%	58%	58%	59%	43%	69%	36%	51%	50%	46%	31%
24	220%	57%	72%	80%	73%	74%	115%	35%	48%	49%	47%	31%
25	342%	51%	68%	68%	63%	55%	65%	44%	56%	57%	51%	43%

Eur Radiol (2024) Zhu L, Dong H, Sun J, et al.

26	237%	54%	67%	64%	66%	54%	70%	40%	52%	52%	49%	37%
27	153%	55%	67%	66%	67%	68%	83%	44%	55%	56%	52%	52%
28	146%	55%	69%	84%	78%	71%	820%	35%	52%	52%	49%	38%
20 mGy												
1	318%	46%	44%	37%	47%	27%	43%	10142%	35%	26%	29%	21%
2	167%	63%	49%	47%	70%	27%	78%	14%	18%	14%	21%	14%
3	140%	56%	54%	51%	68%	35%	31%	20%	25%	20%	35%	20%
4	310%	61%	67%	69%	70%	54%	64%	43%	50%	44%	55%	34%
5	107%	47%	58%	61%	69%	41%	94%	37%	48%	44%	52%	27%
6	101%	38%	66%	58%	71%	46%	99%	34%	44%	35%	49%	35%
7	289%	53%	65%	63%	80%	45%	55%	22%	29%	22%	36%	29%
8	97%	47%	55%	52%	69%	55%	816%	38%	45%	40%	40%	40%
9	123%	52%	62%	60%	65%	49%	220%	34%	40%	36%	41%	33%
10	162%	60%	46%	39%	49%	32%	194%	46%	38%	30%	38%	24%
11	52%	38%	45%	38%	59%	32%	35%	30%	30%	20%	34%	30%
12	63%	59%	68%	73%	80%	55%	33%	32%	30%	21%	35%	31%
13	65%	37%	44%	37%	51%	26%	40%	29%	38%	31%	40%	22%
14	95%	35%	50%	48%	62%	42%	89%	29%	43%	39%	48%	39%
15	157%	52%	55%	55%	83%	56%	90%	85%	44%	39%	43%	46%
16	165%	51%	61%	63%	61%	48%	79%	45%	53%	53%	52%	44%
17	961%	62%	74%	68%	66%	61%	166%	38%	43%	48%	47%	46%
18	257%	58%	65%	68%	69%	76%	51%	41%	49%	42%	48%	52%
19	58%	46%	61%	74%	71%	58%	35%	35%	45%	39%	44%	48%
20	384%	53%	62%	69%	67%	71%	58%	36%	45%	46%	47%	50%
21	217%	78%	81%	80%	76%	67%	107%	46%	46%	47%	50%	40%
22	81%	54%	72%	71%	75%	57%	37%	48%	60%	53%	50%	41%
23	115%	42%	58%	58%	61%	43%	70%	37%	52%	50%	47%	33%
24	219%	58%	71%	77%	73%	73%	113%	38%	49%	50%	48%	36%
25	376%	52%	67%	68%	62%	56%	66%	44%	56%	57%	51%	44%

Eur Radiol (2024) Zhu L, Dong H, Sun J, et al.

26	236%	53%	66%	63%	66%	54%	71%	40%	51%	52%	48%	36%
27	152%	55%	67%	66%	65%	67%	82%	44%	55%	56%	53%	50%
28	147%	55%	68%	85%	75%	71%	142%	36%	52%	51%	49%	39%

Supplementary Figure 1 Heatmap of repeatability of radiomics features

The heatmap of radiomics features in terms of test-retest repeatability at dose levels of 5 mGy, 10 mGy, and 20 mGy, according to Bland-Altman analysis. PCD-CT = photon counting detector CT, dsDECT = dual-source dual-energy CT, rsDECT = rapid kV-switching dual-energy CT, dlDECT = dual-layer dual-energy CT, ssDECT = sequential scanning DECT.

		PCD-CT			dsDECT			rsDECT			dlDECT			ssDECT		
		5 mGy	10 mGy	20 mGy	5 mGy	10 mGy	20 mGy	5 mGy	10 mGy	20 mGy	5 mGy	10 mGy	20 mGy	5 mGy	10 mGy	20 mGy
firstorder	10Percentile	95.7%	96.4%	95.0%	94.3%	92.1%	92.9%	94.3%	93.6%	93.6%	87.1%	86.4%	87.1%	94.3%	94.3%	95.7%
firstorder	90Percentile	93.6%	95.0%	95.0%	93.6%	94.3%	94.3%	94.3%	96.4%	96.4%	92.9%	92.9%	92.9%	92.9%	94.3%	94.3%
firstorder	Energy	91.4%	92.9%	92.1%	94.3%	95.0%	94.3%	92.1%	92.1%	94.3%	92.9%	92.9%	92.9%	92.1%	93.6%	93.6%
firstorder	Entropy	96.4%	95.7%	95.7%	95.7%	91.4%	93.6%	93.6%	92.1%	95.0%	92.1%	92.9%	90.7%	95.7%	95.0%	96.4%
firstorder	InterquartileRange	95.0%	94.3%	95.0%	93.6%	93.6%	90.7%	96.4%	96.4%	95.7%	92.9%	92.9%	92.9%	95.7%	95.0%	95.0%
firstorder	Kurtosis	97.9%	97.1%	97.1%	96.4%	97.1%	96.4%	92.1%	94.3%	93.6%	93.6%	94.3%	95.0%	92.1%	90.0%	90.7%
firstorder	Maximum	97.1%	97.9%	96.4%	93.6%	94.3%	92.1%	93.6%	93.6%	94.3%	89.3%	90.0%	88.6%	97.1%	97.1%	97.1%
firstorder	MeanAbsoluteDeviation	95.0%	94.3%	95.7%	95.0%	95.7%	92.9%	93.6%	93.6%	92.1%	92.9%	92.9%	92.9%	92.9%	93.6%	93.6%
firstorder	Mean	95.7%	95.7%	93.6%	93.6%	94.3%	94.3%	93.6%	93.6%	91.4%	92.9%	92.9%	92.9%	93.6%	94.3%	94.3%
firstorder	Median	94.3%	93.6%	94.3%	92.1%	95.0%	93.6%	94.3%	95.7%	93.6%	92.9%	92.9%	92.9%	92.9%	92.1%	92.9%
firstorder	Minimum	91.4%	94.3%	91.4%	92.9%	94.3%	93.6%	90.7%	91.4%	94.3%	92.1%	92.9%	92.9%	92.1%	93.6%	94.3%
firstorder	Range	95.7%	96.4%	96.4%	94.3%	93.6%	92.9%	92.1%	95.0%	96.4%	92.9%	92.9%	92.9%	96.4%	97.1%	96.4%
firstorder	RobustMeanAbsoluteDeviation	95.0%	95.0%	95.7%	94.3%	95.7%	92.1%	91.4%	94.3%	93.6%	92.9%	92.9%	92.9%	95.0%	91.4%	95.0%
firstorder	RootMeanSquared	94.3%	94.3%	93.6%	94.3%	95.0%	95.7%	96.4%	92.1%	94.3%	92.9%	92.9%	92.9%	92.9%	91.4%	92.9%
firstorder	Skewness	97.1%	97.1%	97.1%	95.0%	95.7%	96.4%	93.6%	94.3%	92.9%	93.6%	93.6%	93.6%	92.1%	92.1%	92.1%
firstorder	TotalEnergy	91.4%	92.9%	92.1%	94.3%	95.0%	94.3%	92.1%	92.1%	94.3%	92.9%	92.9%	92.9%	92.1%	93.6%	93.6%
firstorder	Uniformity	96.4%	97.1%	96.4%	95.7%	97.1%	95.0%	93.6%	92.9%	95.7%	92.1%	94.3%	92.9%	94.3%	92.1%	93.6%
firstorder	Variance	94.3%	94.3%	95.0%	95.0%	94.3%	92.1%	92.9%	93.6%	92.9%	92.9%	92.9%	92.9%	91.4%	92.1%	91.4%
glcm	Autocorrelation	92.9%	93.6%	93.6%	94.3%	92.1%	94.3%	93.6%	92.1%	93.6%	87.9%	89.3%	89.3%	92.9%	94.3%	94.3%
glcm	ClusterProminence	95.0%	94.3%	93.6%	95.0%	93.6%	90.7%	95.0%	93.6%	91.4%	95.7%	93.6%	93.6%	97.1%	96.4%	96.4%
glcm	ClusterShade	97.9%	95.0%	95.7%	92.1%	95.0%	93.6%	96.4%	92.1%	92.1%	92.9%	91.4%	90.0%	90.0%	91.4%	92.9%
glcm	ClusterTendency	94.3%	93.6%	91.4%	92.9%	91.4%	92.9%	93.6%	90.7%	92.9%	92.9%	90.0%	90.7%	97.1%	97.1%	97.1%
glcm	Contrast	95.0%	92.1%	92.9%	92.1%	92.9%	93.6%	91.4%	93.6%	94.3%	90.7%	92.9%	95.0%	94.3%	93.6%	94.3%
glcm	Correlation	95.0%	92.9%	94.3%	90.0%	97.1%	93.6%	92.1%	92.9%	93.6%	92.9%	92.1%	92.9%	95.0%	93.6%	93.6%
glcm	DifferenceAverage	95.7%	95.0%	94.3%	93.6%	92.1%	95.0%	92.9%	93.6%	95.0%	89.3%	88.6%	90.7%	93.6%	93.6%	93.6%
glcm	DifferenceEntropy	96.4%	94.3%	95.7%	93.6%	93.6%	92.9%	90.7%	93.6%	95.0%	92.9%	92.9%	90.7%	94.3%	93.6%	94.3%
glcm	DifferenceVariance	93.6%	92.9%	95.0%	93.6%	94.3%	94.3%	93.6%	96.4%	94.3%	91.4%	91.4%	93.6%	95.0%	93.6%	93.6%
glcm	Id	96.4%	95.0%	95.7%	93.6%	92.1%	90.7%	94.3%	93.6%	95.0%	93.6%	92.1%	91.4%	92.9%	94.3%	92.9%
glcm	Idm	95.7%	95.0%	95.0%	94.3%	92.1%	92.1%	94.3%	93.6%	95.0%	93.6%	92.1%	91.4%	92.1%	93.6%	93.6%
glcm	Idmn	95.0%	92.1%	92.9%	93.6%	92.1%	93.6%	93.6%	93.6%	93.6%	90.7%	92.9%	94.3%	95.7%	92.9%	93.6%
glcm	Idn	95.0%	95.0%	94.3%	92.9%	92.9%	92.9%	93.6%	94.3%	95.0%	89.3%	88.6%	90.7%	93.6%	93.6%	93.6%
glcm	Imc1	94.3%	94.3%	95.0%	95.0%	92.1%	92.1%	92.1%	95.0%	96.4%	94.3%	95.7%	96.4%	95.0%	91.4%	90.7%
glcm	Imc2	94.3%	92.9%	97.1%	92.1%	95.0%	92.1%	92.1%	95.7%	92.9%	93.6%	92.1%	92.1%	91.4%	92.1%	90.0%
glcm	InverseVariance	93.6%	92.9%	94.3%	95.7%	96.4%	92.9%	92.1%	94.3%	95.7%	90.7%	92.1%	92.1%	95.0%	94.3%	93.6%
glcm	JointAverage	95.0%	94.3%	94.3%	92.9%	93.6%	92.9%	92.9%	91.4%	92.9%	90.0%	91.4%	90.0%	92.9%	94.3%	94.3%
glcm	JointEnergy	97.9%	97.1%	97.1%	96.4%	97.1%	95.0%	93.6%	92.9%	95.7%	92.9%	94.3%	94.3%	95.0%	91.4%	92.9%
glcm	JointEntropy	96.4%	95.7%	95.7%	95.7%	91.4%	92.9%	94.3%	92.9%	95.0%	92.1%	92.9%	92.1%	95.0%	94.3%	93.6%
glcm	MCC	96.4%	94.3%	93.6%	92.1%	94.3%	93.6%	92.9%	93.6%	94.3%	90.7%	89.3%	90.7%	93.6%	92.1%	93.6%
glcm	MaximumProbability	97.9%	97.1%	96.4%	95.7%	96.4%	96.4%	92.9%	95.0%	94.3%	92.9%	95.0%	94.3%	92.9%	95.0%	93.6%
glcm	SumAverage	95.0%	94.3%	94.3%	92.9%	93.6%	92.9%	92.9%	91.4%	92.9%	90.0%	91.4%	90.0%	92.9%	94.3%	94.3%
glcm	SumEntropy	96.4%	97.1%	96.4%	95.0%	92.9%	94.3%	93.6%	91.4%	94.3%	91.4%	92.9%	92.1%	95.0%	94.3%	94.3%
glcm	SumSquares	93.6%	93.6%	92.9%	90.0%	92.1%	91.4%	93.6%	91.4%	92.9%	92.1%	89.3%	91.4%	97.1%	97.1%	97.1%
gldm	DependenceEntropy	97.1%	97.9%	96.4%	97.9%	97.1%	92.9%	94.3%	91.4%	94.3%	88.6%	92.1%	89.3%	95.0%	93.6%	94.3%
gldm	DependenceNonUniformity	95.0%	94.3%	95.0%	94.3%	93.6%	95.0%	91.4%	92.9%	97.9%	88.6%	88.6%	88.6%	97.1%	93.6%	93.6%
gldm	DependenceNonUniformityNormalized	95.0%	94.3%	95.0%	94.3%	93.6%	95.0%	91.4%	92.9%	97.9%	88.6%	88.6%	88.6%	97.1%	93.6%	93.6%
gldm	DependenceVariance	95.7%	95.0%	95.7%	94.3%	97.1%	95.0%	95.7%	94.3%	95.0%	93.6%	90.7%	91.4%	92.1%	94.3%	93.6%
gldm	GrayLevelNonUniformity	96.4%	97.1%	96.4%	95.7%	97.1%	95.0%	93.6%	92.9%	95.7%	92.1%	94.3%	92.9%	94.3%	92.1%	93.6%
gldm	GrayLevelVariance	94.3%	93.6%	93.6%	90.0%	91.4%	91.4%	93.6%	92.1%	92.9%	92.1%	90.0%	90.7%	97.1%	97.1%	97.1%
gldm	HighGrayLevelEmphasis	92.9%	94.3%	93.6%	94.3%	92.1%	94.3%	93.6%	92.1%	94.3%	87.9%	89.3%	89.3%	92.9%	94.3%	94.3%
gldm	LargeDependenceEmphasis	95.7%	97.1%	96.4%	96.4%	97.1%	92.9%	92.9%	93.6%	95.7%	93.6%	93.6%	92.9%	92.9%	95.0%	92.9%
gldm	LargeDependenceHighGrayLevelEmphasis	96.4%	95.0%	95.0%	90.7%	95.0%	94.3%	95.7%	94.3%	95.0%	92.9%	90.7%	90.7%	95.0%	95.7%	92.9%
gldm	LargeDependenceLowGrayLevelEmphasis	97.9%	97.9%	97.9%	96.4%	96.4%	93.6%	92.1%	92.9%	92.9%	92.1%	93.6%	92.9%	94.3%	93.6%	95.0%
gldm	LowGrayLevelEmphasis	97.9%	97.9%	97.9%	92.1%	95.7%	95.0%	93.6%	94.3%	95.0%	90.7%	91.4%	91.4%	95.0%	93.6%	92.1%
gldm	SmallDependenceEmphasis	95.0%	95.0%	94.3%	94.3%	93.6%	94.3%	92.9%	92.1%	92.9%	89.3%	88.6%	89.3%	92.9%	91.4%	94.3%
gldm	SmallDependenceHighGrayLevelEmphasis	93.6%	95.7%	93.6%	94.3%	93.6%	92.9%	92.1%	92.1%	91.4%	91.4%	90.0%	91.4%	93.6%	91.4%	94.3%
gldm	SmallDependenceLowGrayLevelEmphasis	94.3%	96.4%	95.7%	92.9%	92.1%	92.9%	92.9%	95.7%	92.1%	93.6%	95.7%	92.1%	94.3%	93.6%	96.4%
glrlm	GrayLevelNonUniformity	95.7%	94.3%	95.7%	96.4%	96.4%	96.4%	92.9%	94.3%	95.0%	90.7%	91.4%	91.4%	92.1%	91.4%	92.1%
glrlm	GrayLevelNonUniformityNormalized	96.4%	97.1%	96.4%	96.4%	95.7%	93.6%	94.3%	95.0%	95.0%	92.1%	92.9%	92.1%	93.6%	92.9%	92.9%
glrlm	GrayLevelVariance	92.9%	93.6%	93.6%	91.4%	92.1%	92.1%	94.3%	92.9%	95.0%	92.9%	90.7%	91.4%	95.7%	95.7%	96.4%
glrlm	HighGrayLevelRunEmphasis	92.9%	94.3%	93.6%	94.3%	92.1%	93.6%	94.3%	95.0%	93.6%	89.3%	89.3%	89.3%	95.0%	95.0%	93.6%
glrlm	LongRunEmphasis	97.9%	97.1%	97.1%	97.9%	97.1%	95.7%	92.9%	92.9%	97.1%	93.6%	93.6%	94.3%	97.1%	93.6%	94.3%
glrlm	LongRunHighGrayLevelEmphasis	94.3%	95.7%	95.0%	99.3%	97.1%	95.7%	92.1%	96.4%	97.1%	90.7%	91.4%	91.4%	98.6%	97.1%	96.4%
glrlm	LongRunLowGrayLevelEmphasis	97.9%	97.9%	97.9%	97.9%	97.9%	96.4%	92.9%	95.0%	91.4%	92.1%	92.9%	95.0%	91.4%	94.3%	94.3%
glrlm	LowGrayLevelRunEmphasis	97.9%	97.9%	97.9%	95.0%	96.4%	92.9%	92.9%	94.3%	92.9%	91.4%	91.4%	94.3%	94.3%	95.7%	94.3%
glrlm	RunEntropy	94.3%	93.6%	93.6%	97.1%	95.7%	92.1%	95.0%	95.7%	94.3%	92.9%	92.9%	93.6%	94.3%	92.1%	93.6%
glrlm	RunLengthNonUniformity	95.0%	95.0%	95.0%	93.6%	93.6%	95.0%	92.1%	92.1%	95.0%	93.6%	92.1%	91.4%	92.9%	93.6%	95.7%
glrlm	RunLengthNonUniformityNormalized	95.0%	95.7%	95.7%	95.0%	92.1%	92.9%	95.0%	94.3%	92.1%	92.9%	93.6%	92.9%	96.4%	95.0%	94.3%
glrlm	RunPercentage	95.7%	97.1%	96.4%	95.0%	95.0%	92.9%	92.9%	93.6%	95.7%	93.6%	94.3%	94.3%	92.1%	95.0%	92.1%
glrlm	RunVariance	97.9%	97.1%	97.9%	97.9%	97.1%	95.7%	90.0%	95.0%	98.6%	93.6%	94.3%	94.3%	96.4%	93.6%	94.3%
glrlm	ShortRunEmphasis	95.7%	97.1%	96.4%	95.0%	91.4%	92.9%	94.3%	94.3%	93.6%	92.9%	93.6%	93.6%	92.9%	92.9%	94.3%
glrlm	ShortRunHighGrayLevelEmphasis	92.9%	92.1%	93.6%	94.3%	92.1%	94.3%	93.6%	94.3%	95.7%	89.3%	90.7%	90.0%	96.4%	95.7%	94.3%
glrlm	ShortRunLowGrayLevelEmphasis	97.9%	97.9%	97.1%	93.6%	94.3%	91.4%	90.0%	94.3%	93.6%	90.0%	90.7%	92.9%	92.9%	92.1%	92.9%
glszm	GrayLevelNonUniformity	95.0%	95.7%	95.7%	95.7%	92.9%	92.9%	94.3%	92.9%	96.4%	89.3%	88.6%	88.6%	95.0%	95.0%	90.0%
glszm	GrayLevelNonUniformityNormalized	95.7%	97.9%	97.1%	96.4%	96.4%	95.0%	92.9%	92.9%	97.1%	92.1%	93.6%	91.4%	93.6%	93.6%	95.7%
glszm	GrayLevelVariance	93.6%	95.7%	92.9%	93.6%	93.6%	94.3%	95.7%	94.3%	93.6%	92.9%	93.6%	94.3%	92.9%	96.4%	94.3%
glszm	HighGrayLevelZoneEmphasis	93.6%	94.3%	94.3%	93.6%	92.9%	95.0%	95.0%	95.0%	93.6%	90.0%	91.4%	90.0%	96.4%	96.4%	95.0%
glszm	LargeAreaEmphasis	98.6%	97.9%	97.9%	97.1%	97.1%	94.3%	92.9%	92.9%	97.1%	94.3%	95.7%	95.0%	97.1%	94.3%	94.3%
glszm	LargeAreaHighGrayLevelEmphasis	95.7%	95.0%	98.6%	98.6%	98.6%	96.4%	93.6%	95.7%	97.1%	95.0%	95.0%	95.7%	98.6%	95.0%	96.4%
glszm	LargeAreaLowGrayLevelEmphasis	98.6%	97.9%	97.9%	96.4%	97.9%	97.1%	94.3%	90.7%	93.6%	94.3%	96.4%	95.0%	92.1%	92.9%	91.4%
glszm	LowGrayLevelZoneEmphasis	97.9%	97.9%	97.9%	95.0%	92.9%	95.7%	91.4%	97.9%	96.4%	92.9%	92.9%	95.7%	92.9%	92.1%	92.9%
glszm	SizeZoneNonUniformity	96.4%	94.3%	94.3%	93.6%	93.6%	95.7%	94.3%	93.6%	92.9%	89.3%	89.3%	91.4%	92.9%	92.1%	93.6%
glszm	SizeZoneNonUniformityNormalized	95.7%	93.6%	93.6%	96.4%	94.3%	92.1%	93.6%	92.1%	95.0%	91.4%	93.6%	92.9%	93.6%	92.9%	96.4%
glszm	SmallAreaEmphasis	95.0%	92.9%	93.6%	93.6%	95.7%	93.6%	92.1%	92.9%	92.9%	91.4%	92.1%	92.1%	94.3%	94.3%	96.4%
glszm	SmallAreaHighGrayLevelEmphasis	94.3%	94.3%	93.6%	92.1%	92.9%	95.0%	94.3%	92.9%	93.6%	87.1%	91.4%	90.7%	94.3%	92.9%	96.4%
glszm	SmallAreaLowGrayLevelEmphasis	97.9%	97.9%	97.9%	94.3%	94.3%	97.1%	94.3%	95.7%	92.1%	92.1%	92.9%	93.6%	90.7%	92.9%	96.4%
glszm	ZoneEntropy	96.4%	97.9%	97.9%	95.7%	95.0%	92.1%	95.7%	92.9%	93.6%	90.7%	92.9%	92.9%	93.6%	92.9%	95.0%
glszm	ZonePercentage	95.7%	95.0%	95.0%	95.0%	92.9%	95.0%	94.3%	93.6%	94.3%	90.0%	88.6%	89.3%	92.1%	93.6%	92.1%
glszm	ZoneVariance	98.6%	97.9%	97.9%	97.1%	97.1%	95.7%	92.1%	92.9%	96.4%	94.3%	95.7%	95.0%	97.1%	94.3%	94.3%
ngtdm	Busyness	97.1%	97.1%	97.1%	97.1%	95.7%	95.7%	93.6%	95.0%	92.1%	95.0%	93.6%	94.3%	93.6%	93.6%	93.6%
ngtdm	Coarseness	93.6%	92.1%	93.6%	97.1%	96.4%	95.0%	91.4%	92.9%	96.4%	90.0%	92.1%	92.9%	94.3%	94.3%	95.7%
ngtdm	Complexity	93.6%	92.1%	93.6%	92.1%	94.3%	95.0%	92.9%	92.9%	92.9%	88.6%	90.7%	92.1%	95.0%	94.3%	94.3%
ngtdm	Contrast	92.9%	92.1%	92.9%	93.6%	94.3%	92.9%	97.1%	92.1%	93.6%	91.4%	93.6%	92.9%	96.4%	97.1%	96.4%
ngtdm	Strength	97.9%	97.1%	94.3%	95.0%	92.9%	95.0%	91.4%	94.3%	92.1%	94.3%	94.3%	91.4%	95.0%	95.0%	94.3%

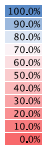

Eur Radiol (2024) Zhu L, Dong H, Sun J, et al.

Supplementary Figure 2 Heatmap of intra-system reproducibility among three dose levels

The heatmap of radiomics features in terms of intra-system reproducibility among three dose levels of 5 versus 10 mGy, 5 versus 20 mGy, and 10 versus 20 mGy, according to ICC and CCC values. PCD-CT = photon counting detector CT, dsDECT = dual-source dual-energy CT, rsDECT = rapid kV-switching dual-energy CT, dlDECT = dual-layer dual-energy CT, ssDECT = sequential scanning DECT, ICC = intraclass correlation coefficient, CCC = concordance correlation coefficient.

(A) Heatmap of intra-system reproducibility by ICC

		PCD-CT			dsDECT			rsDECT			dlDECT			ssDECT		
		5 vs 10 mGy	5 vs 20 mGy	10 vs 20 mGy	5 vs 10 mGy	5 vs 20 mGy	10 vs 20 mGy	5 vs 10 mGy	5 vs 20 mGy	10 vs 20 mGy	5 vs 10 mGy	5 vs 20 mGy	10 vs 20 mGy	5 vs 10 mGy	5 vs 20 mGy	10 vs 20 mGy
firstorder	10Percentile	1.000	1.000	1.000	1.000	1.000	1.000	1.000	1.000	1.000	1.000	1.000	1.000	1.000	0.999	0.999
firstorder	90Percentile	1.000	1.000	1.000	1.000	1.000	1.000	1.000	1.000	1.000	1.000	1.000	1.000	1.000	0.999	0.999
firstorder	Energy	1.000	1.000	1.000	1.000	1.000	1.000	1.000	0.999	1.000	1.000	1.000	1.000	1.000	1.000	1.000
firstorder	Entropy	0.977	0.973	0.984	0.988	0.976	0.994	0.989	0.981	0.993	0.963	0.946	0.971	0.935	0.931	0.946
firstorder	InterquartileRange	1.000	1.000	1.000	1.000	1.000	1.000	1.000	0.999	1.000	0.997	0.997	0.998	1.000	0.980	0.980
firstorder	Kurtosis	0.994	0.982	0.992	0.998	0.996	0.999	0.997	0.996	0.999	0.963	0.961	0.981	0.990	0.964	0.982
firstorder	Maximum	1.000	1.000	1.000	0.999	0.999	1.000	0.999	0.999	1.000	1.000	1.000	1.000	1.000	0.998	0.998
firstorder	MeanAbsoluteDeviation	1.000	1.000	1.000	1.000	1.000	1.000	1.000	0.999	1.000	0.998	0.998	0.999	1.000	0.983	0.983
firstorder	Mean	1.000	1.000	1.000	1.000	1.000	1.000	1.000	1.000	1.000	1.000	1.000	1.000	1.000	1.000	1.000
firstorder	Median	1.000	1.000	1.000	1.000	1.000	1.000	1.000	1.000	1.000	1.000	1.000	1.000	1.000	1.000	1.000
firstorder	Minimum	1.000	1.000	1.000	0.988	0.977	0.993	0.991	0.989	0.995	0.999	0.999	0.999	1.000	0.996	0.996
firstorder	Range	0.999	0.999	0.999	0.999	0.997	0.999	0.999	0.998	0.999	0.996	0.997	0.998	0.999	0.986	0.985
firstorder	RobustMeanAbsoluteDeviation	1.000	1.000	1.000	1.000	1.000	1.000	1.000	0.999	1.000	0.997	0.998	0.999	1.000	0.982	0.982
firstorder	RootMeanSquared	1.000	1.000	1.000	1.000	1.000	1.000	1.000	0.999	1.000	1.000	1.000	1.000	1.000	0.999	0.999
firstorder	Skewness	0.988	0.981	0.993	0.999	0.998	1.000	1.000	0.999	1.000	0.991	0.988	0.995	0.972	0.968	0.976
firstorder	TotalEnergy	1.000	1.000	1.000	1.000	1.000	1.000	1.000	0.999	1.000	1.000	1.000	1.000	1.000	1.000	1.000
firstorder	Uniformity	0.966	0.935	0.967	0.985	0.968	0.990	0.982	0.970	0.986	0.966	0.928	0.969	0.954	0.945	0.960
firstorder	Variance	1.000	1.000	1.000	1.000	1.000	1.000	1.000	0.999	1.000	0.995	0.997	0.997	1.000	0.968	0.968
glcm	Autocorrelation	0.908	0.929	0.945	0.998	0.997	0.999	0.998	0.998	0.999	0.965	0.970	0.983	0.911	0.906	0.921
glcm	ClusterProminence	0.903	0.904	0.939	0.997	0.996	0.998	0.998	0.997	0.997	0.956	0.910	0.952	0.916	0.907	0.916
glcm	ClusterShade	0.927	0.919	0.961	0.999	0.999	0.999	0.999	0.999	0.999	0.986	0.970	0.986	0.942	0.943	0.927
glcm	ClusterTendency	0.942	0.945	0.966	0.999	0.998	0.999	0.999	0.998	0.999	0.948	0.923	0.946	0.929	0.925	0.938
glcm	Contrast	0.969	0.966	0.986	0.991	0.982	0.993	0.994	0.991	0.996	0.935	0.939	0.962	0.943	0.909	0.920
glcm	Correlation	0.972	0.955	0.994	0.998	0.994	0.999	0.999	0.997	0.999	0.989	0.985	0.995	0.925	0.964	0.945
glcm	DifferenceAverage	0.970	0.961	0.986	0.995	0.989	0.997	0.993	0.988	0.995	0.964	0.960	0.978	0.944	0.934	0.939
glcm	DifferenceEntropy	0.969	0.953	0.983	0.992	0.984	0.995	0.989	0.981	0.994	0.970	0.956	0.979	0.938	0.942	0.947
glcm	DifferenceVariance	0.965	0.961	0.982	0.992	0.985	0.994	0.995	0.993	0.997	0.901	0.884	0.935	0.938	0.901	0.915
glcm	Id	0.969	0.947	0.983	0.993	0.988	0.996	0.990	0.983	0.993	0.972	0.943	0.975	0.933	0.949	0.949
glcm	Idm	0.968	0.948	0.983	0.994	0.989	0.997	0.992	0.986	0.994	0.975	0.949	0.978	0.933	0.950	0.949
glcm	Idmn	0.970	0.965	0.986	0.992	0.983	0.994	0.993	0.990	0.996	0.943	0.946	0.967	0.944	0.915	0.925
glcm	Idn	0.970	0.959	0.986	0.995	0.990	0.997	0.993	0.987	0.995	0.969	0.961	0.980	0.944	0.939	0.943
glcm	Imc1	0.930	0.851	0.969	0.995	0.990	0.996	0.989	0.981	0.994	0.976	0.924	0.970	0.892	0.964	0.940
glcm	Imc2	0.970	0.947	0.991	0.996	0.989	0.996	0.993	0.989	0.996	0.987	0.979	0.993	0.918	0.964	0.941
glcm	InverseVariance	0.908	0.857	0.945	0.975	0.959	0.986	0.975	0.958	0.983	0.914	0.746	0.832	0.937	0.922	0.929
glcm	JointAverage	0.928	0.945	0.958	0.999	0.998	0.999	0.999	0.999	0.999	0.974	0.976	0.987	0.921	0.916	0.928
glcm	JointEnergy	0.919	0.823	0.912	0.983	0.963	0.986	0.977	0.960	0.981	0.947	0.844	0.932	0.952	0.940	0.954
glcm	JointEntropy	0.976	0.969	0.983	0.991	0.982	0.995	0.991	0.983	0.994	0.968	0.941	0.972	0.935	0.936	0.949
glcm	MCC	0.974	0.957	0.993	0.996	0.991	0.997	0.997	0.995	0.998	0.986	0.982	0.991	0.930	0.956	0.952
glcm	MaximumProbability	0.937	0.884	0.940	0.980	0.966	0.985	0.973	0.962	0.978	0.926	0.839	0.922	0.912	0.905	0.939
glcm	SumAverage	0.928	0.945	0.958	0.999	0.998	0.999	0.999	0.999	0.999	0.974	0.976	0.987	0.921	0.916	0.928
glcm	SumEntropy	0.977	0.969	0.983	0.985	0.971	0.992	0.986	0.973	0.991	0.963	0.940	0.969	0.934	0.937	0.944
glcm	SumSquares	0.944	0.953	0.964	0.999	0.997	0.999	0.999	0.998	0.998	0.921	0.900	0.927	0.932	0.915	0.930
gldm	DependenceEntropy	0.950	0.903	0.952	0.977	0.956	0.985	0.970	0.945	0.980	0.948	0.929	0.956	0.904	0.913	0.924
gldm	DependenceNonUniformity	0.949	0.877	0.930	0.962	0.927	0.971	0.950	0.908	0.972	0.955	0.944	0.970	0.916	0.917	0.922
gldm	DependenceNonUniformityNormalized	0.949	0.877	0.930	0.962	0.927	0.971	0.950	0.908	0.972	0.955	0.944	0.970	0.916	0.917	0.922
gldm	DependenceVariance	0.940	0.890	0.948	0.980	0.973	0.987	0.974	0.959	0.982	0.904	0.807	0.906	0.873	0.885	0.898
gldm	GrayLevelNonUniformity	0.966	0.935	0.967	0.985	0.968	0.990	0.982	0.970	0.986	0.966	0.928	0.969	0.954	0.945	0.960
gldm	GrayLevelVariance	0.942	0.952	0.962	0.999	0.998	0.999	0.999	0.998	0.998	0.921	0.898	0.925	0.928	0.911	0.927
gldm	HighGrayLevelEmphasis	0.914	0.934	0.948	0.998	0.997	0.999	0.998	0.998	0.999	0.966	0.971	0.984	0.912	0.906	0.921
gldm	LargeDependenceEmphasis	0.952	0.903	0.965	0.986	0.977	0.992	0.984	0.972	0.989	0.944	0.843	0.931	0.906	0.943	0.946
gldm	LargeDependenceHighGrayLevelEmphasis	0.770	0.769	0.948	0.985	0.981	0.992	0.991	0.989	0.991	0.930	0.935	0.957	0.845	0.898	0.934
gldm	LargeDependenceLowGrayLevelEmphasis	0.967	0.890	0.919	0.998	0.994	0.997	0.995	0.991	0.998	0.872	0.619	0.803	0.909	0.907	0.986
gldm	LowGrayLevelEmphasis	0.888	0.892	0.887	0.999	0.997	0.998	0.998	0.997	0.999	0.922	0.872	0.884	0.900	0.892	0.971
gldm	SmallDependenceEmphasis	0.962	0.947	0.981	0.995	0.990	0.996	0.991	0.985	0.994	0.972	0.965	0.981	0.932	0.940	0.937
gldm	SmallDependenceHighGrayLevelEmphasis	0.955	0.955	0.974	0.995	0.991	0.995	0.992	0.989	0.994	0.964	0.966	0.979	0.925	0.908	0.927
gldm	SmallDependenceLowGrayLevelEmphasis	0.671	0.757	0.783	0.977	0.967	0.982	0.982	0.974	0.990	0.834	0.843	0.848	0.683	0.692	0.766
glrlm	GrayLevelNonUniformity	0.894	0.835	0.909	0.979	0.956	0.978	0.967	0.946	0.969	0.935	0.871	0.915	0.915	0.919	0.916
glrlm	GrayLevelNonUniformityNormalized	0.975	0.968	0.980	0.969	0.918	0.958	0.967	0.956	0.971	0.974	0.963	0.979	0.953	0.949	0.961
glrlm	GrayLevelVariance	0.933	0.943	0.955	0.996	0.991	0.997	0.994	0.989	0.995	0.914	0.856	0.919	0.914	0.900	0.912
glrlm	HighGrayLevelRunEmphasis	0.911	0.932	0.945	0.983	0.974	0.986	0.973	0.965	0.986	0.962	0.968	0.981	0.907	0.900	0.917
glrlm	LongRunEmphasis	0.741	0.313	0.532	0.978	0.960	0.983	0.960	0.927	0.978	0.897	0.686	0.840	0.920	0.911	0.926
glrlm	LongRunHighGrayLevelEmphasis	0.550	0.386	0.672	0.955	0.936	0.959	0.942	0.892	0.953	0.930	0.911	0.942	0.830	0.880	0.928
glrlm	LongRunLowGrayLevelEmphasis	0.922	0.349	0.539	0.990	0.974	0.991	0.973	0.961	0.996	0.848	0.568	0.778	0.926	0.887	0.951
glrlm	LowGrayLevelRunEmphasis	0.897	0.875	0.875	0.994	0.981	0.992	0.993	0.990	0.997	0.924	0.852	0.901	0.914	0.903	0.971
glrlm	RunEntropy	0.898	0.823	0.942	0.985	0.978	0.987	0.970	0.953	0.976	0.938	0.870	0.938	0.859	0.912	0.895
glrlm	RunLengthNonUniformity	0.965	0.944	0.981	0.996	0.993	0.997	0.993	0.989	0.995	0.972	0.946	0.975	0.924	0.944	0.943
glrlm	RunLengthNonUniformityNormalized	0.965	0.941	0.980	0.993	0.990	0.995	0.989	0.986	0.993	0.973	0.943	0.973	0.922	0.945	0.945
glrlm	RunPercentage	0.963	0.929	0.976	0.990	0.983	0.995	0.988	0.979	0.991	0.962	0.900	0.956	0.916	0.947	0.947
glrlm	RunVariance	0.697	0.268	0.491	0.978	0.961	0.983	0.962	0.934	0.978	0.853	0.599	0.782	0.918	0.858	0.887
glrlm	ShortRunEmphasis	0.966	0.937	0.978	0.992	0.989	0.994	0.987	0.984	0.992	0.970	0.925	0.964	0.919	0.948	0.949
glrlm	ShortRunHighGrayLevelEmphasis	0.929	0.941	0.954	0.987	0.980	0.988	0.977	0.970	0.987	0.964	0.968	0.981	0.908	0.896	0.909
glrlm	ShortRunLowGrayLevelEmphasis	0.818	0.833	0.828	0.959	0.885	0.954	0.948	0.922	0.980	0.924	0.911	0.920	0.884	0.874	0.940
glszm	GrayLevelNonUniformity	0.935	0.894	0.966	0.992	0.985	0.992	0.981	0.966	0.989	0.975	0.962	0.981	0.880	0.931	0.925
glszm	GrayLevelNonUniformityNormalized	0.947	0.908	0.906	0.894	0.882	0.898	0.715	0.682	0.864	0.912	0.888	0.912	0.905	0.907	0.920
glszm	GrayLevelVariance	0.833	0.827	0.850	0.985	0.981	0.989	0.975	0.971	0.987	0.909	0.897	0.947	0.781	0.822	0.808
glszm	HighGrayLevelZoneEmphasis	0.896	0.916	0.933	0.973	0.963	0.976	0.958	0.938	0.962	0.937	0.944	0.963	0.886	0.881	0.899
glszm	LargeAreaEmphasis	0.926	0.723	0.827	0.988	0.982	0.990	0.979	0.960	0.986	0.763	0.590	0.857	0.940	0.944	0.975
glszm	LargeAreaHighGrayLevelEmphasis	0.749	0.672	0.893	0.974	0.961	0.977	0.979	0.944	0.974	0.815	0.774	0.877	0.473	0.387	0.679
glszm	LargeAreaLowGrayLevelEmphasis	0.968	0.757	0.841	0.996	0.993	0.994	0.985	0.977	0.995	0.641	0.315	0.656	0.922	0.899	0.958
glszm	LowGrayLevelZoneEmphasis	0.867	0.886	0.868	0.936	0.923	0.970	0.938	0.887	0.913	0.932	0.917	0.932	0.957	0.931	0.957
glszm	SizeZoneNonUniformity	0.956	0.948	0.978	0.987	0.976	0.987	0.975	0.967	0.982	0.960	0.961	0.975	0.932	0.927	0.923
glszm	SizeZoneNonUniformityNormalized	0.921	0.888	0.945	0.790	0.735	0.803	0.737	0.734	0.810	0.919	0.916	0.924	0.912	0.909	0.905
glszm	SmallAreaEmphasis	0.904	0.849	0.921	0.764	0.727	0.782	0.716	0.712	0.771	0.910	0.903	0.907	0.895	0.896	0.896
glszm	SmallAreaHighGrayLevelEmphasis	0.912	0.921	0.934	0.919	0.904	0.917	0.909	0.898	0.904	0.930	0.939	0.954	0.870	0.849	0.875
glszm	SmallAreaLowGrayLevelEmphasis	0.685	0.677	0.617	0.823	0.834	0.880	0.861	0.782	0.865	0.882	0.836	0.873	0.892	0.834	0.868
glszm	ZoneEntropy	0.932	0.888	0.945	0.902	0.870	0.910	0.894	0.887	0.917	0.908	0.881	0.898	0.903	0.920	0.908
glszm	ZonePercentage	0.964	0.949	0.982	0.996	0.991	0.996	0.993	0.988	0.996	0.976	0.968	0.982	0.928	0.945	0.942
glszm	ZoneVariance	0.925	0.721	0.826	0.988	0.982	0.990	0.979	0.961	0.986	0.752	0.580	0.855	0.939	0.941	0.974
ngtdm	Busyness	0.924	0.883	0.923	0.993	0.981	0.987	0.987	0.988	0.994	0.944	0.905	0.929	0.935	0.895	0.907
ngtdm	Coarseness	0.892	0.718	0.876	0.967	0.948	0.974	0.957	0.916	0.952	0.967	0.928	0.967	0.887	0.941	0.900
ngtdm	Complexity	0.964	0.948	0.980	0.977	0.965	0.983	0.976	0.964	0.984	0.936	0.934	0.956	0.925	0.894	0.902
ngtdm	Contrast	0.940	0.948	0.965	0.991	0.988	0.995	0.988	0.977	0.992	0.871	0.873	0.903	0.931	0.864	0.882
ngtdm	Strength	0.905	0.694	0.855	0.990	0.988	0.994	0.987	0.984	0.994	0.955	0.921	0.972	0.862	0.932	0.914

1.000
0.900
0.800
0.700
0.600
0.500
0.400
0.300
0.200
0.100
0.000

(B) Heatmap of intra-system reproducibility by CCC

		PCD-CT			dsDECT			rsDECT			dIDECT			ssDECT		
		5 vs 10 mGy	5 vs 20 mGy	10 vs 20 mGy	5 vs 10 mGy	5 vs 20 mGy	10 vs 20 mGy	5 vs 10 mGy	5 vs 20 mGy	10 vs 20 mGy	5 vs 10 mGy	5 vs 20 mGy	10 vs 20 mGy	5 vs 10 mGy	5 vs 20 mGy	10 vs 20 mGy
firstorder	10Percentile	1.000	1.000	1.000	1.000	1.000	1.000	1.000	1.000	1.000	1.000	1.000	1.000	1.000	0.999	0.999
firstorder	90Percentile	1.000	1.000	1.000	1.000	1.000	1.000	1.000	1.000	1.000	1.000	1.000	1.000	1.000	0.999	0.999
firstorder	Energy	1.000	1.000	1.000	1.000	1.000	1.000	1.000	0.999	1.000	1.000	1.000	1.000	1.000	1.000	1.000
firstorder	Entropy	0.977	0.973	0.984	0.988	0.976	0.994	0.989	0.981	0.993	0.963	0.946	0.971	0.934	0.931	0.946
firstorder	InterquartileRange	1.000	1.000	1.000	1.000	1.000	1.000	1.000	0.999	1.000	0.997	0.997	0.998	1.000	0.980	0.980
firstorder	Kurtosis	0.994	0.981	0.992	0.998	0.996	0.999	0.997	0.996	0.999	0.962	0.961	0.981	0.990	0.964	0.981
firstorder	Maximum	1.000	1.000	1.000	0.999	0.999	1.000	0.999	0.999	1.000	1.000	1.000	1.000	1.000	0.998	0.998
firstorder	MeanAbsoluteDeviation	1.000	1.000	1.000	1.000	1.000	1.000	1.000	0.999	1.000	0.998	0.998	0.999	1.000	0.983	0.983
firstorder	Mean	1.000	1.000	1.000	1.000	1.000	1.000	1.000	1.000	1.000	1.000	1.000	1.000	1.000	1.000	1.000
firstorder	Median	1.000	1.000	1.000	1.000	1.000	1.000	1.000	1.000	1.000	1.000	1.000	1.000	1.000	1.000	1.000
firstorder	Minimum	1.000	1.000	1.000	0.988	0.976	0.993	0.990	0.989	0.995	0.999	0.999	0.999	1.000	0.996	0.996
firstorder	Range	0.999	0.999	0.999	0.999	0.997	0.999	0.999	0.998	0.999	0.996	0.997	0.998	0.999	0.986	0.985
firstorder	RobustMeanAbsoluteDeviation	1.000	1.000	1.000	1.000	1.000	1.000	1.000	0.999	1.000	0.997	0.998	0.999	1.000	0.981	0.982
firstorder	RootMeanSquared	1.000	1.000	1.000	1.000	1.000	1.000	1.000	0.999	1.000	1.000	1.000	1.000	1.000	0.999	0.999
firstorder	Skewness	0.988	0.981	0.993	0.999	0.998	1.000	1.000	0.999	1.000	0.991	0.988	0.995	0.972	0.968	0.975
firstorder	TotalEnergy	1.000	1.000	1.000	1.000	1.000	1.000	1.000	0.999	1.000	1.000	1.000	1.000	1.000	1.000	1.000
firstorder	Uniformity	0.966	0.934	0.967	0.985	0.968	0.990	0.982	0.970	0.986	0.966	0.928	0.969	0.954	0.945	0.960
firstorder	Variance	1.000	1.000	1.000	1.000	1.000	1.000	1.000	0.999	1.000	0.995	0.997	0.997	1.000	0.968	0.968
glcm	Autocorrelation	0.907	0.929	0.945	0.998	0.997	0.999	0.998	0.998	0.999	0.965	0.970	0.983	0.910	0.906	0.920
glcm	ClusterProminence	0.903	0.903	0.939	0.997	0.996	0.998	0.998	0.997	0.997	0.956	0.910	0.951	0.915	0.907	0.916
glcm	ClusterShade	0.927	0.918	0.961	0.999	0.999	0.999	0.999	0.999	0.999	0.986	0.970	0.986	0.942	0.943	0.927
glcm	ClusterTendency	0.942	0.945	0.966	0.999	0.998	0.999	0.999	0.998	0.999	0.947	0.923	0.946	0.928	0.925	0.938
glcm	Contrast	0.969	0.966	0.986	0.991	0.982	0.993	0.994	0.991	0.996	0.934	0.939	0.961	0.943	0.909	0.920
glcm	Correlation	0.972	0.955	0.994	0.998	0.994	0.999	0.999	0.997	0.999	0.989	0.984	0.995	0.924	0.964	0.945
glcm	DifferenceAverage	0.970	0.961	0.986	0.995	0.989	0.997	0.993	0.988	0.995	0.964	0.960	0.978	0.944	0.934	0.939
glcm	DifferenceEntropy	0.969	0.952	0.983	0.992	0.984	0.995	0.989	0.981	0.994	0.969	0.956	0.978	0.938	0.942	0.947
glcm	DifferenceVariance	0.964	0.961	0.981	0.992	0.984	0.994	0.995	0.993	0.997	0.901	0.884	0.934	0.938	0.901	0.915
glcm	Id	0.968	0.947	0.983	0.993	0.987	0.996	0.990	0.983	0.993	0.972	0.942	0.974	0.933	0.949	0.949
glcm	Idm	0.968	0.948	0.983	0.994	0.989	0.997	0.992	0.985	0.994	0.975	0.949	0.978	0.933	0.950	0.949
glcm	Idmn	0.970	0.965	0.986	0.992	0.982	0.994	0.993	0.990	0.996	0.942	0.946	0.967	0.944	0.915	0.925
glcm	Idn	0.970	0.958	0.986	0.995	0.990	0.997	0.992	0.987	0.995	0.969	0.961	0.980	0.943	0.939	0.942
glcm	Imc1	0.930	0.851	0.969	0.995	0.990	0.996	0.989	0.981	0.994	0.976	0.924	0.969	0.891	0.964	0.940
glcm	Imc2	0.970	0.947	0.991	0.996	0.989	0.996	0.993	0.989	0.996	0.987	0.979	0.993	0.918	0.963	0.941
glcm	InverseVariance	0.908	0.856	0.945	0.975	0.958	0.986	0.975	0.958	0.983	0.913	0.745	0.831	0.937	0.922	0.928
glcm	JointAverage	0.927	0.945	0.958	0.999	0.998	0.999	0.999	0.999	0.999	0.974	0.976	0.987	0.921	0.916	0.928
glcm	JointEnergy	0.919	0.822	0.911	0.983	0.963	0.986	0.976	0.960	0.981	0.947	0.843	0.932	0.952	0.939	0.954
glcm	JointEntropy	0.976	0.969	0.983	0.991	0.982	0.995	0.991	0.983	0.994	0.968	0.941	0.972	0.934	0.936	0.949
glcm	MCC	0.974	0.957	0.992	0.996	0.991	0.997	0.997	0.995	0.998	0.986	0.982	0.991	0.930	0.956	0.952
glcm	MaximumProbability	0.937	0.884	0.940	0.980	0.965	0.985	0.973	0.961	0.978	0.926	0.839	0.922	0.911	0.905	0.938
glcm	SumAverage	0.927	0.945	0.958	0.999	0.998	0.999	0.999	0.999	0.999	0.974	0.976	0.987	0.921	0.916	0.928
glcm	SumEntropy	0.977	0.969	0.983	0.993	0.985	0.992	0.986	0.973	0.991	0.963	0.940	0.969	0.934	0.937	0.944
glcm	SumSquares	0.944	0.953	0.964	0.999	0.997	0.999	0.999	0.998	0.998	0.921	0.899	0.926	0.932	0.915	0.930
gldm	DependenceEntropy	0.950	0.902	0.952	0.977	0.956	0.985	0.970	0.945	0.980	0.948	0.928	0.956	0.903	0.912	0.924
gldm	DependenceNonUniformity	0.949	0.877	0.929	0.962	0.927	0.971	0.950	0.908	0.972	0.954	0.944	0.970	0.916	0.917	0.922
gldm	DependenceNonUniformityNormalized	0.949	0.877	0.929	0.962	0.927	0.971	0.950	0.908	0.972	0.954	0.944	0.970	0.916	0.917	0.922
gldm	DependenceVariance	0.939	0.890	0.948	0.980	0.973	0.987	0.974	0.959	0.982	0.904	0.806	0.906	0.872	0.885	0.898
gldm	GrayLevelNonUniformity	0.966	0.934	0.967	0.985	0.968	0.990	0.982	0.970	0.986	0.966	0.928	0.969	0.954	0.945	0.960
gldm	GrayLevelVariance	0.942	0.952	0.962	0.999	0.998	0.999	0.999	0.998	0.998	0.920	0.898	0.925	0.928	0.911	0.926
gldm	HighGrayLevelEmphasis	0.913	0.934	0.947	0.998	0.997	0.999	0.998	0.998	0.999	0.966	0.971	0.983	0.912	0.906	0.921
gldm	LargeDependenceEmphasis	0.952	0.902	0.964	0.986	0.977	0.992	0.984	0.972	0.989	0.944	0.843	0.931	0.906	0.942	0.946
gldm	LargeDependenceHighGrayLevelEmphasis	0.770	0.768	0.948	0.985	0.981	0.992	0.991	0.989	0.991	0.930	0.935	0.957	0.845	0.898	0.934
gldm	LargeDependenceLowGrayLevelEmphasis	0.967	0.889	0.918	0.998	0.994	0.997	0.995	0.991	0.998	0.871	0.618	0.803	0.908	0.969	0.966
gldm	LowGrayLevelEmphasis	0.887	0.892	0.886	0.999	0.997	0.998	0.998	0.997	0.999	0.922	0.822	0.883	0.900	0.892	0.971
gldm	SmallDependenceEmphasis	0.961	0.947	0.981	0.995	0.990	0.996	0.991	0.985	0.994	0.972	0.965	0.981	0.932	0.940	0.936
gldm	SmallDependenceHighGrayLevelEmphasis	0.955	0.955	0.974	0.995	0.991	0.995	0.992	0.989	0.994	0.964	0.966	0.979	0.925	0.908	0.927
gldm	SmallDependenceLowGrayLevelEmphasis	0.671	0.756	0.782	0.977	0.966	0.982	0.981	0.974	0.990	0.834	0.842	0.847	0.682	0.692	0.766
glrlm	GrayLevelNonUniformity	0.894	0.835	0.909	0.978	0.956	0.978	0.967	0.946	0.969	0.934	0.871	0.914	0.915	0.919	0.916
glrlm	GrayLevelNonUniformityNormalized	0.974	0.968	0.979	0.969	0.918	0.958	0.967	0.956	0.971	0.974	0.963	0.979	0.953	0.949	0.961
glrlm	GrayLevelVariance	0.933	0.943	0.955	0.996	0.991	0.997	0.994	0.989	0.995	0.914	0.856	0.919	0.913	0.900	0.912
glrlm	HighGrayLevelRunEmphasis	0.910	0.932	0.945	0.983	0.974	0.986	0.972	0.964	0.986	0.962	0.968	0.981	0.907	0.900	0.916
glrlm	LongRunEmphasis	0.740	0.312	0.531	0.978	0.960	0.983	0.960	0.927	0.977	0.897	0.685	0.839	0.920	0.910	0.926
glrlm	LongRunHighGrayLevelEmphasis	0.549	0.385	0.671	0.955	0.936	0.959	0.942	0.892	0.953	0.929	0.911	0.942	0.830	0.880	0.927
glrlm	LongRunLowGrayLevelEmphasis	0.921	0.348	0.538	0.990	0.974	0.991	0.973	0.961	0.996	0.848	0.567	0.777	0.926	0.886	0.951
glrlm	LowGrayLevelRunEmphasis	0.897	0.874	0.874	0.994	0.980	0.992	0.993	0.990	0.997	0.924	0.852	0.900	0.914	0.902	0.971
glrlm	RunEntropy	0.898	0.823	0.942	0.985	0.977	0.987	0.970	0.953	0.976	0.938	0.870	0.938	0.858	0.912	0.894
glrlm	RunLengthNonUniformity	0.965	0.944	0.981	0.996	0.993	0.997	0.993	0.989	0.995	0.972	0.946	0.975	0.924	0.944	0.943
glrlm	RunLengthNonUniformityNormalized	0.965	0.941	0.980	0.993	0.990	0.995	0.989	0.986	0.993	0.973	0.943	0.973	0.922	0.945	0.945
glrlm	RunPercentage	0.963	0.929	0.976	0.990	0.983	0.995	0.988	0.979	0.991	0.962	0.899	0.956	0.916	0.947	0.947
glrlm	RunVariance	0.697	0.267	0.490	0.978	0.960	0.983	0.962	0.934	0.978	0.853	0.598	0.782	0.918	0.857	0.887
glrlm	ShortRunEmphasis	0.966	0.937	0.978	0.992	0.989	0.993	0.987	0.984	0.992	0.970	0.925	0.964	0.918	0.948	0.949
glrlm	ShortRunHighGrayLevelEmphasis	0.929	0.941	0.954	0.987	0.980	0.988	0.977	0.970	0.987	0.964	0.968	0.981	0.908	0.895	0.908
glrlm	ShortRunLowGrayLevelEmphasis	0.818	0.832	0.828	0.959	0.884	0.954	0.948	0.922	0.980	0.923	0.910	0.919	0.884	0.873	0.940
glszm	GrayLevelNonUniformity	0.935	0.894	0.966	0.992	0.985	0.992	0.981	0.965	0.989	0.975	0.962	0.981	0.879	0.931	0.925
glszm	GrayLevelNonUniformityNormalized	0.947	0.908	0.906	0.893	0.881	0.897	0.714	0.681	0.864	0.912	0.887	0.912	0.905	0.906	0.920
glszm	GrayLevelVariance	0.833	0.827	0.849	0.985	0.981	0.989	0.975	0.970	0.987	0.909	0.897	0.947	0.780	0.821	0.808
glszm	HighGrayLevelZoneEmphasis	0.896	0.915	0.933	0.973	0.963	0.976	0.958	0.938	0.962	0.937	0.944	0.963	0.886	0.881	0.898
glszm	LargeAreaEmphasis	0.926	0.722	0.826	0.988	0.982	0.990	0.979	0.960	0.986	0.762	0.589	0.856	0.940	0.944	0.975
glszm	LargeAreaHighGrayLevelEmphasis	0.748	0.671	0.893	0.974	0.961	0.977	0.979	0.944	0.974	0.814	0.773	0.877	0.472	0.386	0.678
glszm	LargeAreaLowGrayLevelEmphasis	0.968	0.756	0.840	0.996	0.993	0.994	0.985	0.977	0.995	0.640	0.315	0.655	0.922	0.898	0.958
glszm	LowGrayLevelZoneEmphasis	0.867	0.886	0.868	0.936	0.922	0.970	0.938	0.886	0.913	0.931	0.917	0.932	0.957	0.930	0.957
glszm	SizeZoneNonUniformity	0.956	0.948	0.978	0.987	0.976	0.987	0.975	0.967	0.982	0.960	0.960	0.975	0.932	0.927	0.923
glszm	SizeZoneNonUniformityNormalized	0.921	0.888	0.944	0.789	0.735	0.802	0.736	0.733	0.809	0.919	0.916	0.924	0.912	0.909	0.905
glszm	SmallAreaEmphasis	0.904	0.849	0.921	0.764	0.726	0.782	0.716	0.712	0.770	0.909	0.903	0.906	0.895	0.896	0.896
glszm	SmallAreaHighGrayLevelEmphasis	0.912	0.921	0.934	0.919	0.904	0.916	0.908	0.898	0.903	0.930	0.938	0.954	0.870	0.848	0.875
glszm	SmallAreaLowGrayLevelEmphasis	0.684	0.676	0.616	0.823	0.834	0.880	0.861	0.782	0.864	0.882	0.835	0.873	0.892	0.834	0.867
glszm	ZoneEntropy	0.932	0.888	0.945	0.902	0.870	0.910	0.894	0.886	0.916	0.908	0.881	0.898	0.903	0.919	0.907
glszm	ZonePercentage	0.963	0.949	0.982	0.996	0.991	0.996	0.993	0.988	0.996	0.976	0.968	0.982	0.927	0.945	0.941
glszm	ZoneVariance	0.924	0.721	0.825	0.988	0.982	0.990	0.979	0.961	0.986	0.751	0.580	0.855	0.939	0.941	0.974
ngtdm	Busyness	0.923	0.883	0.923	0.993	0.981	0.987	0.987	0.988	0.994	0.944	0.905	0.929	0.935	0.895	0.906
ngtdm	Coarseness	0.892	0.717	0.875	0.967	0.948	0.974	0.957	0.916	0.952	0.967	0.928	0.966	0.887	0.941	0.900
ngtdm	Complexity	0.964	0.948	0.980	0.977	0.965	0.983	0.976	0.964	0.984	0.935	0.934	0.956	0.925	0.894	0.902
ngtdm	Contrast	0.940	0.948	0.965	0.991	0.988	0.995	0.988	0.977	0.992	0.871	0.872	0.903	0.930	0.864	0.881
ngtdm	Strength	0.905	0.693	0.854	0.990	0.988	0.994	0.987	0.984	0.994	0.955	0.920	0.972	0.862	0.932	0.914

1.000
0.900
0.800
0.700
0.600
0.500
0.400
0.300
0.200
0.100
0.000

Eur Radiol (2024) Zhu L, Dong H, Sun J, et al.

Supplementary Figure 3 Heatmap of inter-system reproducibility within the same dose level

The heatmap of radiomics features in terms of inter-system reproducibility among five scanners within the same dose level of 5 mGy, 10 mGy, and 20 mGy, according to ICC and CCC values. PCD-CT = photon counting detector CT, dsDECT = dual-source dual-energy CT, rsDECT = rapid kV-switching dual-energy CT, dlDECT = dual-layer dual-energy CT, ssDECT = sequential scanning DECT, ICC = intraclass correlation coefficient, CCC = concordance correlation coefficient.

(A) Heatmap of inter-system reproducibility by ICC

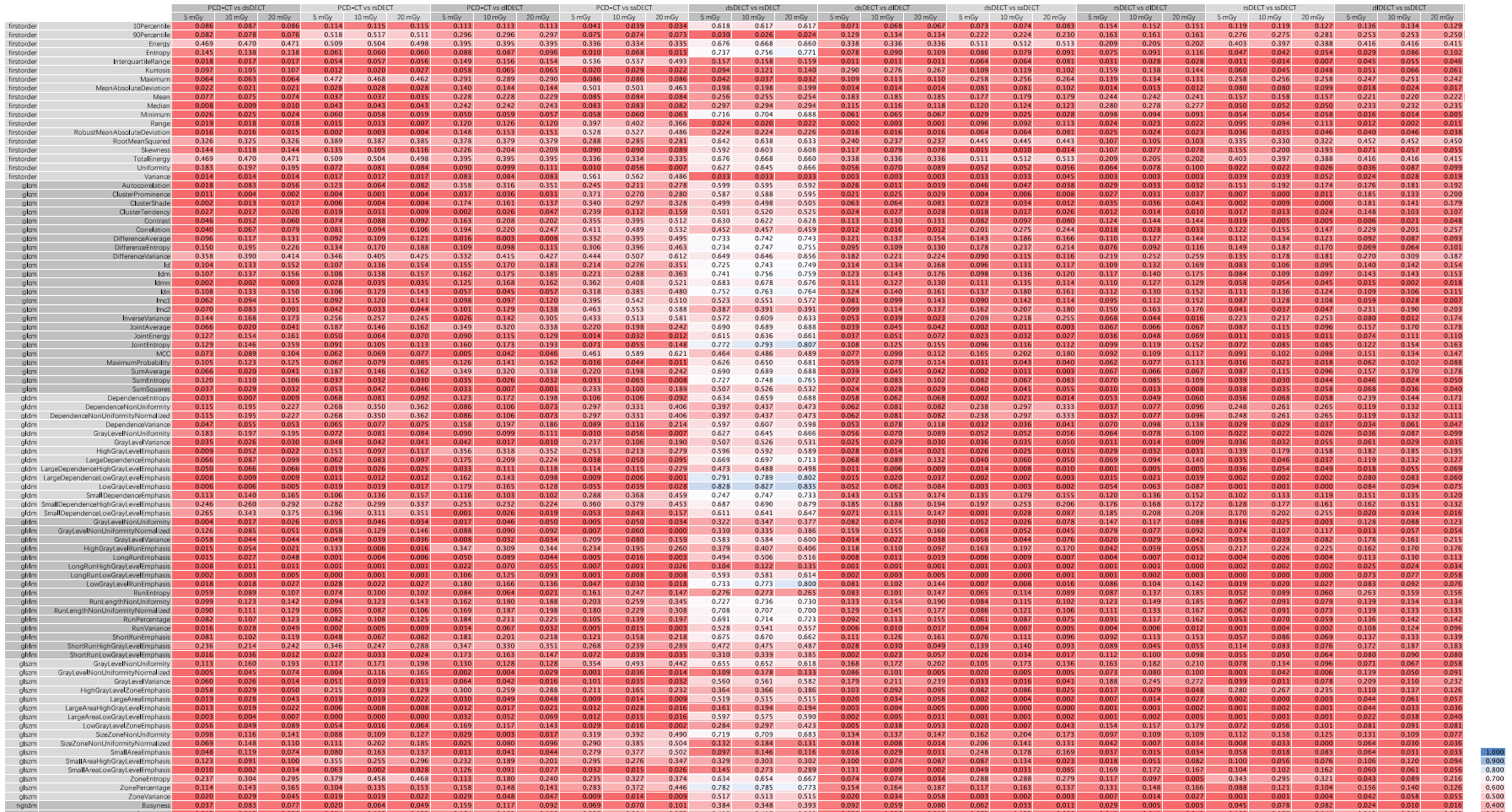

Eur Radiol (2024) Zhu L, Dong H, Sun J, et al.

(B) Heatmap of inter-system reproducibility with CCC

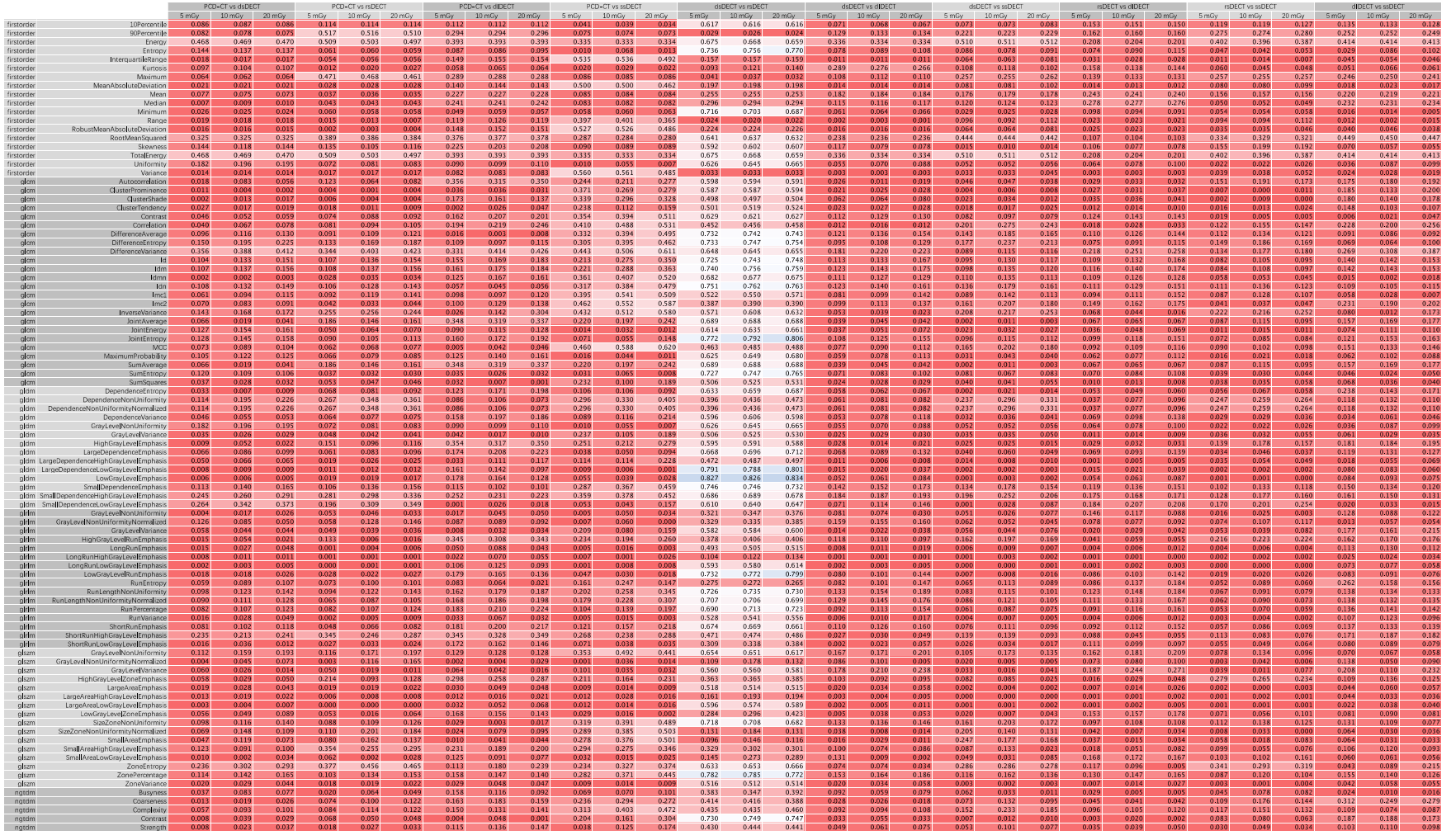
PCD-CT vs dsDECT
PCD-CT vs rsDECT
PCD-CT vs diDECT
PCD-CT vs ssDECT
dsDECT vs rsDECT
dsDECT vs diDECT
dsDECT vs ssDECT
rsDECT vs diDECT
rsDECT vs ssDECT
diDECT vs ssDECT
5 mGy
10 mGy
20 mGy
firstorder 10Percentile 0.086 0.087 0.086 0.114 0.114 0.114 0.112 0.112 0.112 0.041 0.039 0.034 0.617 0.616 0.616 0.071 0.068 0.067 0.073 0.073 0.083 0.153 0.151 0.150 0.119 0.119 0.127 0.135 0.133 0.128
firstorder 90Percentile 0.082 0.078 0.075 0.517 0.516 0.510 0.294 0.294 0.296 0.075 0.074 0.073 0.028 0.028 0.024 0.129 0.133 0.134 0.221 0.223 0.229 0.162 0.160 0.160 0.275 0.274 0.280 0.252 0.252 0.249
firstorder Energy 0.468 0.469 0.470 0.509 0.503 0.497 0.393 0.393 0.393 0.335 0.333 0.334 0.675 0.668 0.659 0.336 0.334 0.334 0.510 0.511 0.512 0.208 0.204 0.201 0.402 0.396 0.387 0.414 0.414 0.413
firstorder Entropy 0.144 0.137 0.137 0.061 0.060 0.059 0.087 0.086 0.095 0.010 0.068 0.013 0.736 0.756 0.770 0.078 0.089 0.108 0.086 0.078 0.091 0.074 0.090 0.115 0.047 0.042 0.053 0.029 0.086 0.102
firstorder InterquartileRange 0.018 0.017 0.017 0.054 0.056 0.056 0.149 0.155 0.154 0.535 0.536 0.492 0.157 0.157 0.159 0.011 0.011 0.011 0.064 0.063 0.081 0.031 0.028 0.028 0.011 0.014 0.007 0.045 0.054 0.046
firstorder Kurtosis 0.097 0.104 0.107 0.012 0.020 0.027 0.058 0.065 0.064 0.020 0.029 0.022 0.093 0.121 0.140 0.289 0.276 0.266 0.108 0.118 0.102 0.158 0.138 0.144 0.060 0.045 0.048 0.051 0.066 0.061
firstorder Maximum 0.064 0.062 0.064 0.471 0.468 0.461 0.289 0.288 0.288 0.086 0.085 0.086 0.041 0.037 0.032 0.108 0.112 0.110 0.257 0.255 0.262 0.139 0.133 0.131 0.257 0.255 0.257 0.246 0.250 0.241
firstorder MeanAbsoluteDeviation 0.021 0.021 0.021 0.028 0.028 0.028 0.140 0.144 0.143 0.500 0.500 0.462 0.197 0.198 0.198 0.014 0.014 0.014 0.081 0.081 0.102 0.014 0.013 0.012 0.080 0.080 0.099 0.018 0.023 0.017
firstorder Mean 0.077 0.075 0.073 0.037 0.036 0.035 0.227 0.227 0.228 0.085 0.084 0.084 0.255 0.255 0.253 0.182 0.184 0.184 0.176 0.179 0.178 0.243 0.241 0.240 0.156 0.157 0.156 0.220 0.219 0.221
firstorder Median 0.007 0.009 0.010 0.043 0.043 0.043 0.241 0.241 0.242 0.083 0.082 0.082 0.296 0.294 0.294 0.115 0.116 0.117 0.128 0.124 0.123 0.278 0.277 0.276 0.050 0.052 0.049 0.232 0.231 0.234
firstorder Minimum 0.026 0.025 0.024 0.060 0.058 0.058 0.049 0.059 0.057 0.058 0.060 0.063 0.716 0.703 0.687 0.061 0.064 0.066 0.029 0.025 0.028 0.098 0.094 0.091 0.054 0.054 0.058 0.016 0.014 0.005
firstorder Range 0.019 0.018 0.018 0.015 0.013 0.007 0.119 0.126 0.119 0.397 0.401 0.365 0.024 0.020 0.022 0.002 0.003 0.001 0.096 0.092 0.112 0.023 0.023 0.021 0.094 0.094 0.112 0.012 0.002 0.015
firstorder RobustMeanAbsoluteDeviation 0.016 0.016 0.015 0.002 0.003 0.004 0.148 0.152 0.151 0.527 0.526 0.486 0.224 0.224 0.226 0.016 0.016 0.016 0.064 0.064 0.081 0.025 0.023 0.023 0.035 0.035 0.046 0.040 0.046 0.038
firstorder RootMeanSquared 0.325 0.325 0.325 0.389 0.386 0.384 0.376 0.377 0.378 0.287 0.284 0.280 0.641 0.637 0.632 0.238 0.236 0.236 0.444 0.444 0.442 0.107 0.104 0.103 0.334 0.329 0.321 0.449 0.450 0.447
firstorder Skewness 0.144 0.118 0.144 0.135 0.105 0.116 0.225 0.203 0.208 0.090 0.089 0.089 0.592 0.602 0.607 0.117 0.079 0.078 0.015 0.010 0.014 0.106 0.077 0.078 0.155 0.199 0.192 0.070 0.037 0.055
firstorder TotalEnergy 0.468 0.469 0.470 0.509 0.503 0.497 0.393 0.393 0.393 0.335 0.333 0.334 0.675 0.668 0.659 0.336 0.334 0.334 0.510 0.511 0.512 0.208 0.204 0.201 0.402 0.396 0.387 0.414 0.414 0.413
firstorder Uniformity 0.182 0.196 0.195 0.072 0.081 0.083 0.090 0.099 0.110 0.010 0.055 0.007 0.626 0.645 0.665 0.055 0.070 0.088 0.052 0.052 0.056 0.064 0.078 0.100 0.022 0.022 0.026 0.036 0.087 0.099
firstorder Variance 0.014 0.014 0.014 0.017 0.017 0.017 0.082 0.083 0.083 0.560 0.561 0.485 0.033 0.033 0.033 0.003 0.003 0.003 0.033 0.033 0.045 0.003 0.003 0.003 0.039 0.038 0.052 0.024 0.028 0.019
glcm Autocorrelation 0.018 0.083 0.036 0.123 0.064 0.082 0.356 0.315 0.350 0.244 0.211 0.277 0.598 0.594 0.591 0.026 0.013 0.019 0.046 0.047 0.038 0.029 0.033 0.032 0.151 0.191 0.173 0.175 0.180 0.192
glcm ClusterProminence 0.011 0.004 0.002 0.004 0.001 0.004 0.036 0.036 0.031 0.371 0.269 0.279 0.587 0.587 0.594 0.021 0.025 0.028 0.004 0.006 0.008 0.027 0.031 0.037 0.007 0.000 0.011 0.185 0.133 0.200
glcm ClusterShade 0.002 0.013 0.017 0.006 0.004 0.004 0.173 0.161 0.137 0.339 0.296 0.328 0.498 0.497 0.504 0.062 0.064 0.080 0.023 0.034 0.012 0.035 0.036 0.041 0.002 0.009 0.000 0.180 0.140 0.178
glcm ClusterTendency 0.027 0.017 0.019 0.018 0.011 0.009 0.002 0.026 0.047 0.238 0.112 0.159 0.501 0.519 0.524 0.023 0.027 0.028 0.018 0.017 0.025 0.012 0.014 0.010 0.016 0.013 0.024 0.148 0.103 0.107
glcm Contrast 0.046 0.052 0.059 0.074 0.088 0.092 0.162 0.207 0.201 0.354 0.394 0.511 0.629 0.621 0.627 0.112 0.129 0.130 0.082 0.097 0.078 0.124 0.143 0.143 0.019 0.005 0.005 0.006 0.021 0.047
glcm Correlation 0.040 0.067 0.078 0.081 0.094 0.105 0.194 0.219 0.246 0.410 0.488 0.531 0.452 0.456 0.458 0.012 0.016 0.012 0.201 0.275 0.243 0.018 0.028 0.033 0.122 0.155 0.147 0.228 0.200 0.256
glcm DifferenceAverage 0.096 0.116 0.130 0.091 0.109 0.121 0.016 0.003 0.008 0.332 0.394 0.495 0.732 0.742 0.743 0.121 0.136 0.154 0.143 0.185 0.165 0.110 0.126 0.144 0.112 0.134 0.121 0.091 0.086 0.092
glcm DifferenceEntropy 0.150 0.195 0.225 0.133 0.169 0.187 0.109 0.097 0.115 0.305 0.395 0.462 0.733 0.747 0.754 0.095 0.108 0.129 0.177 0.237 0.213 0.075 0.091 0.115 0.149 0.186 0.169 0.069 0.064 0.100
glcm DifferenceVariance 0.350 0.388 0.412 0.344 0.403 0.423 0.331 0.414 0.426 0.443 0.506 0.611 0.648 0.655 0.655 0.181 0.220 0.223 0.089 0.135 0.116 0.218 0.251 0.258 0.134 0.177 0.180 0.269 0.308 0.387
glcm Id 0.104 0.133 0.151 0.107 0.136 0.154 0.155 0.169 0.183 0.213 0.275 0.350 0.725 0.743 0.748 0.113 0.133 0.167 0.095 0.130 0.117 0.109 0.132 0.168 0.082 0.105 0.095 0.140 0.142 0.153
glcm Idm 0.107 0.137 0.156 0.108 0.137 0.156 0.161 0.175 0.184 0.221 0.288 0.363 0.740 0.756 0.759 0.123 0.143 0.175 0.098 0.135 0.120 0.116 0.140 0.174 0.084 0.108 0.097 0.142 0.143 0.153
glcm Idmn 0.002 0.002 0.003 0.028 0.035 0.034 0.125 0.167 0.161 0.361 0.407 0.520 0.682 0.677 0.675 0.111 0.127 0.129 0.110 0.135 0.113 0.109 0.126 0.128 0.058 0.053 0.045 0.015 0.002 0.018
glcm Idn 0.108 0.132 0.149 0.106 0.128 0.143 0.057 0.045 0.056 0.317 0.384 0.479 0.751 0.762 0.763 0.123 0.140 0.161 0.336 0.179 0.161 0.111 0.129 0.151 0.111 0.136 0.123 0.109 0.105 0.115
glcm Imc1 0.061 0.094 0.115 0.092 0.119 0.141 0.098 0.097 0.120 0.395 0.541 0.509 0.522 0.550 0.571 0.081 0.099 0.142 0.089 0.142 0.113 0.094 0.111 0.152 0.087 0.128 0.107 0.058 0.028 0.007
glcm Imc2 0.070 0.083 0.091 0.042 0.033 0.044 0.100 0.129 0.138 0.462 0.552 0.587 0.387 0.390 0.390 0.099 0.113 0.137 0.161 0.207 0.180 0.149 0.162 0.175 0.041 0.037 0.047 0.231 0.190 0.202
glcm InverseVariance 0.143 0.168 0.172 0.255 0.256 0.244 0.026 0.142 0.304 0.432 0.512 0.580 0.571 0.608 0.632 0.053 0.039 0.023 0.208 0.217 0.253 0.068 0.044 0.016 0.222 0.216 0.252 0.080 0.012 0.173
glcm JointAverage 0.066 0.019 0.041 0.186 0.146 0.161 0.348 0.319 0.337 0.220 0.197 0.242 0.689 0.688 0.688 0.039 0.045 0.042 0.002 0.011 0.003 0.067 0.065 0.067 0.087 0.115 0.095 0.157 0.169 0.177
glcm JointEnergy 0.127 0.154 0.161 0.050 0.064 0.070 0.090 0.115 0.128 0.014 0.032 0.012 0.614 0.635 0.661 0.037 0.051 0.072 0.023 0.032 0.027 0.036 0.048 0.069 0.011 0.015 0.011 0.074 0.111 0.110
glcm JointEntropy 0.128 0.145 0.158 0.090 0.105 0.113 0.160 0.172 0.192 0.071 0.055 0.148 0.772 0.792 0.806 0.108 0.125 0.155 0.096 0.115 0.112 0.099 0.118 0.151 0.072 0.085 0.084 0.121 0.153 0.163
glcm MCC 0.073 0.089 0.104 0.062 0.068 0.077 0.005 0.042 0.046 0.460 0.588 0.620 0.463 0.485 0.488 0.077 0.090 0.112 0.165 0.202 0.180 0.092 0.109 0.116 0.090 0.102 0.098 0.151 0.133 0.146
glcm MaximumProbability 0.100 0.122 0.125 0.068 0.079 0.085 0.122 0.140 0.161 0.078 0.044 0.011 0.625 0.649 0.659 0.078 0.078 0.113 0.031 0.047 0.040 0.062 0.077 0.122 0.016 0.021 0.018 0.062 0.102 0.088
glcm SumAverage 0.066 0.019 0.041 0.186 0.146 0.161 0.348 0.319 0.337 0.220 0.197 0.242 0.689 0.688 0.688 0.039 0.045 0.042 0.002 0.011 0.003 0.067 0.065 0.067 0.087 0.115 0.095 0.157 0.169 0.177
glcm SumEntropy 0.120 0.109 0.106 0.037 0.032 0.030 0.035 0.026 0.032 0.011 0.065 0.008 0.727 0.747 0.765 0.071 0.083 0.102 0.081 0.067 0.083 0.070 0.084 0.108 0.039 0.030 0.044 0.046 0.024 0.050
glcm SumSquares 0.037 0.028 0.032 0.053 0.047 0.046 0.032 0.007 0.001 0.232 0.100 0.189 0.506 0.525 0.531 0.024 0.028 0.029 0.040 0.041 0.055 0.010 0.013 0.008 0.038 0.035 0.058 0.068 0.036 0.040
glcm DependenceEntropy 0.034 0.007 0.009 0.068 0.081 0.092 0.122 0.171 0.198 0.106 0.106 0.092 0.633 0.659 0.687 0.018 0.026 0.043 0.062 0.067 0.067 0.002 0.021 0.041 0.063 0.060 0.056 0.067 0.058 0.238 0.143 0.171
glcm DependenceNonUniformity 0.114 0.195 0.226 0.267 0.348 0.361 0.086 0.106 0.073 0.296 0.330 0.405 0.396 0.436 0.473 0.061 0.081 0.082 0.237 0.296 0.331 0.037 0.077 0.096 0.247 0.259 0.264 0.118 0.132 0.110
glcm DependenceNonUniformityNormalized 0.114 0.195 0.226 0.267 0.348 0.361 0.086 0.106 0.073 0.296 0.330 0.405 0.396 0.436 0.473 0.061 0.081 0.082 0.237 0.296 0.331 0.037 0.077 0.096 0.247 0.259 0.264 0.118 0.132 0.110
glcm DependenceVariance 0.046 0.055 0.053 0.064 0.077 0.075 0.158 0.197 0.186 0.089 0.116 0.214 0.596 0.606 0.598 0.053 0.078 0.118 0.032 0.036 0.041 0.069 0.098 0.138 0.029 0.029 0.036 0.034 0.061 0.046
glcm GrayLevelNonUniformity 0.182 0.196 0.195 0.072 0.081 0.083 0.090 0.099 0.110 0.010 0.055 0.007 0.626 0.645 0.665 0.055 0.070 0.088 0.052 0.052 0.056 0.064 0.078 0.100 0.022 0.022 0.026 0.036 0.087 0.099
glcm GrayLevelVariance 0.035 0.026 0.029 0.048 0.042 0.041 0.042 0.017 0.010 0.237 0.105 0.189 0.506 0.525 0.530 0.025 0.029 0.030 0.035 0.035 0.050 0.011 0.014 0.009 0.036 0.032 0.055 0.061 0.029 0.035
glcm HighGrayLevelEmphasis 0.009 0.052 0.022 0.151 0.096 0.116 0.354 0.317 0.350 0.251 0.212 0.279 0.595 0.591 0.588 0.028 0.014 0.021 0.025 0.025 0.015 0.029 0.032 0.031 0.139 0.178 0.157 0.181 0.184 0.195
glcm LargeDependenceEmphasis 0.066 0.086 0.099 0.061 0.083 0.096 0.174 0.208 0.223 0.018 0.050 0.094 0.668 0.696 0.712 0.068 0.089 0.132 0.040 0.060 0.049 0.069 0.093 0.139 0.034 0.046 0.037 0.119 0.131 0.127
glcm LargeDependenceHighGrayLevelEmphasis 0.050 0.066 0.065 0.019 0.028 0.025 0.031 0.113 0.117 0.114 0.114 0.228 0.472 0.487 0.497 0.014 0.009 0.018 0.008 0.008 0.010 0.001 0.005 0.005 0.035 0.054 0.049 0.018 0.053 0.069
glcm LargeDependenceLowGrayLevelEmphasis 0.008 0.009 0.009 0.011 0.012 0.012 0.161 0.142 0.097 0.009 0.006 0.001 0.791 0.788 0.801 0.015 0.020 0.037 0.002 0.002 0.003 0.015 0.021 0.039 0.002 0.002 0.002 0.080 0.083 0.060
glcm LowGrayLevelEmphasis 0.006 0.006 0.005 0.019 0.019 0.017 0.178 0.164 0.128 0.055 0.039 0.028 0.827 0.826 0.834 0.052 0.061 0.084 0.003 0.003 0.002 0.054 0.063 0.087 0.001 0.001 0.000 0.084 0.093 0.075
glcm SmallDependenceEmphasis 0.113 0.140 0.165 0.106 0.136 0.156 0.115 0.102 0.101 0.287 0.367 0.459 0.746 0.746 0.732 0.142 0.152 0.173 0.134 0.178 0.154 0.119 0.136 0.151 0.102 0.133 0.118 0.150 0.134 0.120
glcm SmallDependenceHighGrayLevelEmphasis 0.245 0.260 0.291 0.281 0.298 0.336 0.252 0.231 0.223 0.359 0.378 0.452 0.686 0.689 0.678 0.184 0.187 0.193 0.196 0.252 0.206 0.175 0.168 0.171 0.128 0.177 0.160 0.161 0.150 0.131
glcm SmallDependenceLowGrayLevelEmphasis 0.264 0.342 0.373 0.198 0.309 0.349 0.001 0.026 0.018 0.053 0.043 0.157 0.610 0.640 0.647 0.071 0.114 0.146 0.001 0.028 0.087 0.184 0.207 0.208 0.126 0.201 0.254 0.026 0.033 0.015
glrlm GrayLevelNonUniformity 0.004 0.017 0.026 0.053 0.046 0.033 0.017 0.045 0.050 0.005 0.050 0.034 0.321 0.347 0.376 0.081 0.074 0.030 0.051 0.026 0.077 0.146 0.117 0.088 0.016 0.025 0.003 0.128 0.088 0.122
glrlm GrayLevelNonUniformityNormalized 0.126 0.085 0.050 0.058 0.128 0.146 0.087 0.089 0.092 0.007 0.060 0.000 0.329 0.335 0.385 0.159 0.155 0.160 0.062 0.052 0.045 0.078 0.077 0.092 0.074 0.107 0.117 0.013 0.057 0.054
glrlm GrayLevelVariance 0.058 0.044 0.044 0.049 0.039 0.036 0.008 0.024 0.034 0.209 0.080 0.159 0.582 0.584 0.600 0.014 0.022 0.038 0.058 0.044 0.076 0.020 0.029 0.042 0.033 0.039 0.082 0.177 0.161 0.215
glrlm HighGrayLevelRunEmphasis 0.015 0.056 0.021 0.133 0.096 0.116 0.345 0.308 0.343 0.234 0.194 0.260 0.378 0.406 0.406 0.118 0.110 0.097 0.162 0.197 0.169 0.041 0.059 0.055 0.216 0.223 0.224 0.162 0.170 0.176
glrlm LongRunEmphasis 0.015 0.027 0.048 0.001 0.004 0.006 0.050 0.088 0.043 0.005 0.016 0.003 0.493 0.505 0.515 0.008 0.011 0.019 0.006 0.009 0.007 0.004 0.006 0.012 0.004 0.006 0.004 0.113 0.130 0.112
glrlm LongRunHighGrayLevelEmphasis 0.008 0.011 0.011 0.001 0.001 0.001 0.022 0.070 0.055 0.007 0.001 0.026 0.104 0.122 0.134 0.001 0.001 0.001 0.001 0.003 0.002 0.001 0.001 0.000 0.002 0.002 0.002 0.025 0.024 0.034
glrlm LongRunLowGrayLevelEmphasis 0.002 0.003 0.005 0.000 0.001 0.001 0.106 0.125 0.093 0.001 0.008 0.008 0.593 0.580 0.614 0.002 0.001 0.005 0.000 0.000 0.001 0.001 0.002 0.003 0.000 0.000 0.000 0.003 0.077 0.058
glrlm LowGrayLevelRunEmphasis 0.018 0.018 0.026 0.028 0.022 0.027 0.179 0.165 0.136 0.047 0.030 0.018 0.772 0.772 0.799 0.030 0.030 0.044 0.007 0.008 0.016 0.006 0.010 0.142 0.019 0.020 0.026 0.083 0.093 0.076
glrlm RunEntropy 0.059 0.089 0.107 0.073 0.100 0.101 0.083 0.064 0.021 0.161 0.247 0.147 0.275 0.272 0.265 0.082 0.101 0.147 0.065 0.113 0.089 0.086 0.137 0.184 0.052 0.089 0.060 0.262 0.158 0.156
glrlm RunLengthNonUniformity 0.098 0.123 0.142 0.094 0.122 0.143 0.162 0.179 0.187 0.202 0.258 0.345 0.726 0.735 0.730 0.133 0.154 0.189 0.083 0.115 0.101 0.123 0.148 0.184 0.067 0.091 0.079 0.138 0.134 0.133
glrlm RunLengthNonUniformityNormalized 0.090 0.111 0.128 0.065 0.087 0.105 0.168 0.186 0.198 0.179 0.228 0.307 0.707 0.706 0.699 0.129 0.145 0.176 0.086 0.121 0.105 0.111 0.133 0.167 0.062 0.090 0.073 0.138 0.132 0.135
glrlm RunPercentage 0.088 0.107 0.123 0.082 0.107 0.124 0.183 0.210 0.224 0.104 0.139 0.197 0.690 0.713 0.723 0.092 0.113 0.154 0.061 0.087 0.075 0.093 0.116 0.161 0.053 0.078 0.059 0.136 0.141 0.142
glrlm RunVariance 0.016 0.028 0.049 0.002 0.005 0.009 0.033 0.067 0.032 0.005 0.015 0.003 0.528 0.541 0.556 0.006 0.010 0.017 0.004 0.007 0.005 0.004 0.006 0.012 0.003 0.004 0.002 0.107 0.123 0.096
glrlm ShortRunEmphasis 0.081 0.102 0.118 0.048 0.066 0.082 0.181 0.200 0.217 0.121 0.157 0.218 0.674 0.669 0.661 0.110 0.126 0.160 0.076 0.111 0.096 0.092 0.112 0.152 0.057 0.086 0.069 0.137 0.133 0.139
glrlm ShortRunHighGrayLevelEmphasis 0.235 0.213 0.241 0.345 0.246 0.287 0.345 0.328 0.349 0.268 0.238 0.288 0.471 0.474 0.486 0.022 0.030 0.049 0.139 0.139 0.093 0.088 0.045 0.055 0.113 0.083 0.076 0.171 0.187 0.182
glrlm ShortRunLowGrayLevelEmphasis 0.036 0.036 0.012 0.027 0.033 0.024 0.172 0.162 0.146 0.071 0.038 0.015 0.309 0.338 0.384 0.002 0.002 0.023 0.057 0.026 0.034 0.017 0.111 0.499 0.097 0.055 0.049 0.064 0.080 0.089
glszm GrayLevelNonUniformity 0.112 0.159 0.193 0.116 0.171 0.197 0.129 0.128 0.128 0.353 0.492 0.441 0.654 0.651 0.617 0.167 0.171 0.201 0.105 0.173 0.135 0.162 0.181 0.209 0.078 0.134 0.096 0.070 0.067 0.058
glszm GrayLevelNonUniformityNormalized 0.004 0.045 0.073 0.003 0.116 0.165 0.002 0.004 0.029 0.001 0.036 0.014 0.109 0.178 0.132 0.086 0.101 0.005 0.020 0.005 0.005 0.073 0.080 0.100 0.003 0.042 0.006 0.138 0.050 0.090
glszm GrayLevelVariance 0.060 0.026 0.014 0.050 0.019 0.011 0.064 0.042 0.016 0.101 0.035 0.032 0.560 0.560 0.581 0.178 0.210 0.238 0.033 0.016 0.041 0.187 0.244 0.271 0.039 0.011 0.077 0.208 0.110 0.232
glszm HighGrayLevelZoneEmphasis 0.058 0.029 0.030 0.214 0.093 0.128 0.298 0.258 0.287 0.211 0.164 0.231 0.363 0.365 0.385 0.103 0.092 0.082 0.095 0.082 0.085 0.025 0.016 0.029 0.048 0.279 0.265 0.234 0.109 0.136 0.125
glszm LargeAreaEmphasis 0.019 0.028 0.043 0.019 0.019 0.022 0.030 0.049 0.048 0.009 0.014 0.009 0.518 0.514 0.515 0.020 0.034 0.058 0.002 0.004 0.002 0.007 0.014 0.026 0.002 0.000 0.003 0.044 0.060 0.057
glszm LargeAreaHighGrayLevelEmphasis 0.013 0.019 0.022 0.006 0.008 0.008 0.012 0.016 0.021 0.012 0.028 0.016 0.161 0.193 0.194 0.003 0.004 0.005 0.000 0.000 0.000 0.001 0.001 0.002 0.001 0.002 0.001 0.044 0.033 0.036
glszm LargeAreaLowGrayLevelEmphasis 0.003 0.004 0.007 0.000 0.000 0.000 0.032 0.052 0.068 0.012 0.014 0.016 0.596 0.574 0.589 0.002 0.005 0.011 0.001 0.001 0.002 0.001 0.002 0.005 0.001 0.001 0.001 0.022 0.038 0.040
glszm LowGrayLevelZoneEmphasis 0.056 0.049 0.089 0.053 0.018 0.064 0.168 0.156 0.143 0.029 0.016 0.002 0.284 0.298 0.423 0.005 0.038 0.053 0.020 0.007 0.043 0.153 0.157 0.178 0.071 0.056 0.101 0.081 0.090 0.081
glszm SizeZoneNonUniformity 0.098 0.116 0.140 0.088 0.109 0.126 0.029 0.003 0.017 0.319 0.391 0.489 0.718 0.708 0.682 0.133 0.136 0.146 0.161 0.203 0.172 0.097 0.108 0.108 0.112 0.138 0.125 0.131 0.109 0.077
glszm SizeZoneNonUniformityNormalized 0.069 0.148 0.109 0.110 0.201 0.184 0.024 0.079 0.095 0.289 0.385 0.503 0.131 0.184 0.131 0.038 0.008 0.014 0.205 0.140 0.131 0.042 0.007 0.034 0.008 0.033 0.000 0.064 0.030 0.036
glszm SmallAreaEmphasis 0.047 0.119 0.073 0.080 0.162 0.137 0.010 0.041 0.044 0.278 0.376 0.501 0.096 0.146 0.116 0.016 0.029 0.011 0.247 0.177 0.168 0.037 0.015 0.034 0.058 0.018 0.083 0.064 0.031 0.033
glszm SmallAreaHighGrayLevelEmphasis 0.123 0.091 0.100 0.354 0.255 0.295 0.231 0.189 0.200 0.294 0.275 0.346 0.329 0.302 0.301 0.100 0.074 0.086 0.087 0.133 0.023 0.018 0.051 0.082 0.099 0.055 0.076 0.106 0.120 0.093
glszm SmallAreaLowGrayLevelEmphasis 0.010 0.002 0.034 0.062 0.002 0.028 0.125 0.091 0.077 0.012 0.015 0.025 0.145 0.273 0.289 0.131 0.009 0.002 0.049 0.031 0.085 0.168 0.172 0.167 0.103 0.102 0.161 0.060 0.061 0.056
glszm ZoneEntropy 0.236 0.302 0.293 0.377 0.456 0.465 0.113 0.180 0.239 0.234 0.327 0.374 0.633 0.653 0.666 0.074 0.074 0.034 0.286 0.286 0.278 0.117 0.096 0.005 0.341 0.293 0.319 0.043 0.089 0.215
glszm ZonePercentage 0.114 0.142 0.165 0.103 0.134 0.153 0.158 0.147 0.140 0.282 0.371 0.445 0.782 0.785 0.772 0.153 0.164 0.186 0.116 0.162 0.136 0.130 0.147 0.165 0.087 0.120 0.104 0.155 0.140 0.126
glszm ZoneVariance 0.020 0.029 0.044 0.018 0.015 0.022 0.029 0.048 0.047 0.009 0.014 0.008 0.516 0.512 0.514 0.020 0.034 0.058 0.003 0.002 0.003 0.007 0.014 0.027 0.003 0.003 0.004 0.042 0.058 0.055
ngtdm Busyness 0.037 0.083 0.077 0.020 0.064 0.049 0.158 0.116 0.092 0.069 0.070 0.101 0.383 0.347 0.392 0.092 0.059 0.079 0.062 0.033 0.011 0.029 0.005 0.005 0.045 0.078 0.082 0.024 0.010 0.016
ngtdm Coarseness 0.013 0.019 0.026 0.074 0.100 0.122 0.163 0.183 0.159 0.236 0.294 0.272 0.414 0.416 0.388 0.028 0.026 0.018 0.073 0.132 0.095 0.045 0.041 0.042 0.109 0.176 0.144 0.312 0.249 0.279
ngtdm Complexity 0.057 0.093 0.101 0.084 0.114 0.122 0.150 0.131 0.141 0.313 0.403 0.472 0.435 0.435 0.460 0.092 0.094 0.108 0.152 0.232 0.185 0.096 0.105 0.120 0.117 0.151 0.132 0.109 0.074 0.087
ngtdm Contrast 0.008 0.039 0.029 0.068 0.050 0.048 0.004 0.048 0.001 0.204 0.161 0.304 0.730 0.749 0.747 0.033 0.055 0.033 0.007 0.012 0.010 0.003 0.020 0.002 0.083 0.080 0.063 0.187 0.188 0.173
ngtdm Strength 0.008 0.023 0.037 0.018 0.027 0.033 0.115 0.136 0.147 0.018 0.125 0.174 0.430 0.444 0.441 0.049 0.061 0.075 0.053 0.101 0.077 0.035 0.039 0.050 0.030 0.049 0.034 0.103 0.110 0.098
1.000
0.900
0.800
0.700
0.600
0.500
0.400
0.300
0.200
0.100
0.000

Eur Radiol (2024) Zhu L, Dong H, Sun J, et al.

Supplementary Figure 4 Heatmap of inter-system variability among five scanners according to materials
The heatmap of radiomics features in terms of inter-system variability among five scanners within the same dose level of 5 mGy, 10 mGy, and 20 mGy, according to CV and QCD values. PCD-CT = photon counting detector CT, dsDECT = dual-source dual-energy CT, rsDECT = rapid kV-switching dual-energy CT, dlDECT = dual-layer dual-energy CT, ssDECT = sequential scanning DECT, CV = coefficient of variation, QCD = quartile coefficient of dispersion.

(A) Heatmap of inter-system variability at 5 mGy by CV

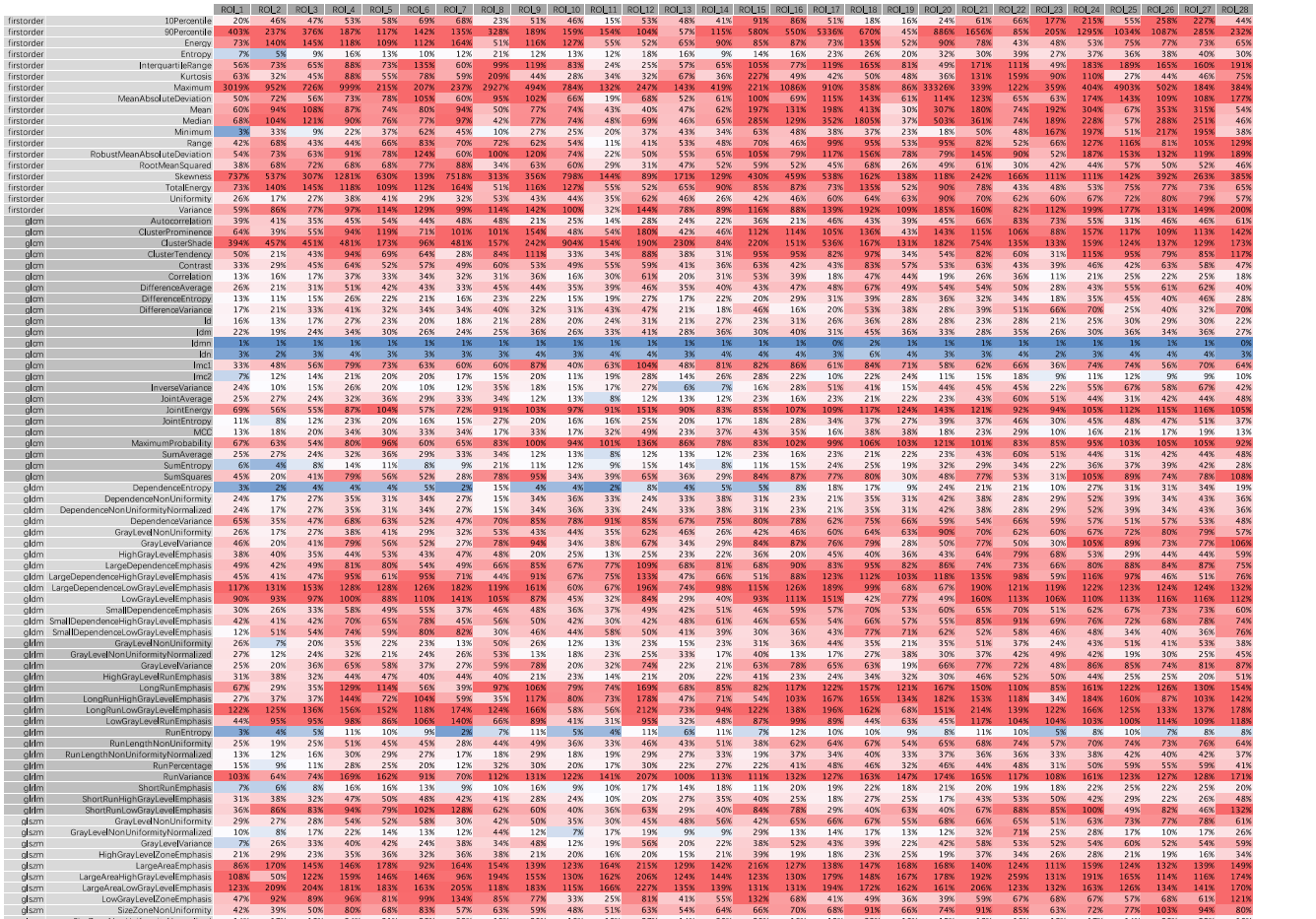

(B) Heatmap of inter-system variability at 5 mGy by QCD

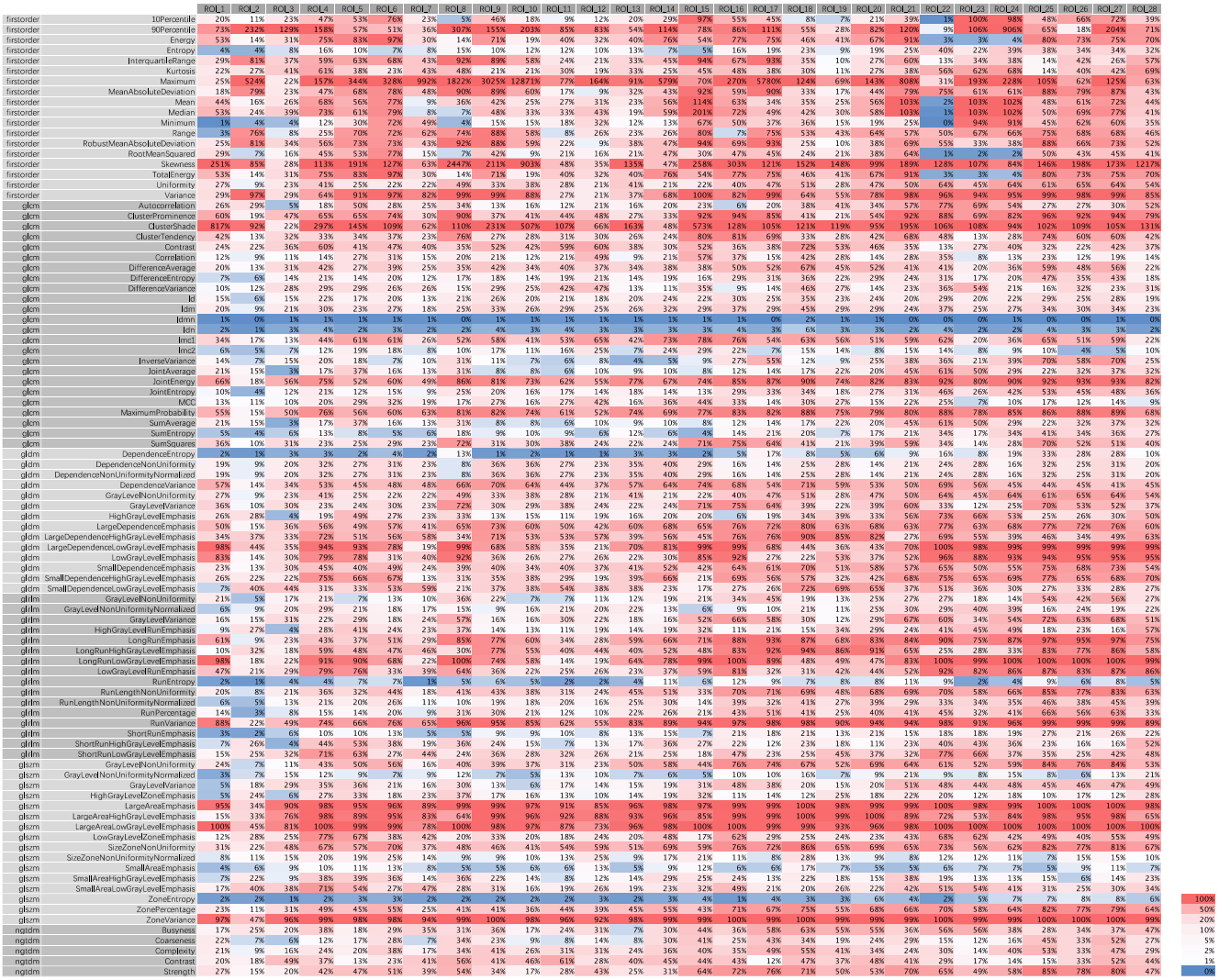

(C) Heatmap of inter-system variability at 10 mGy by CV

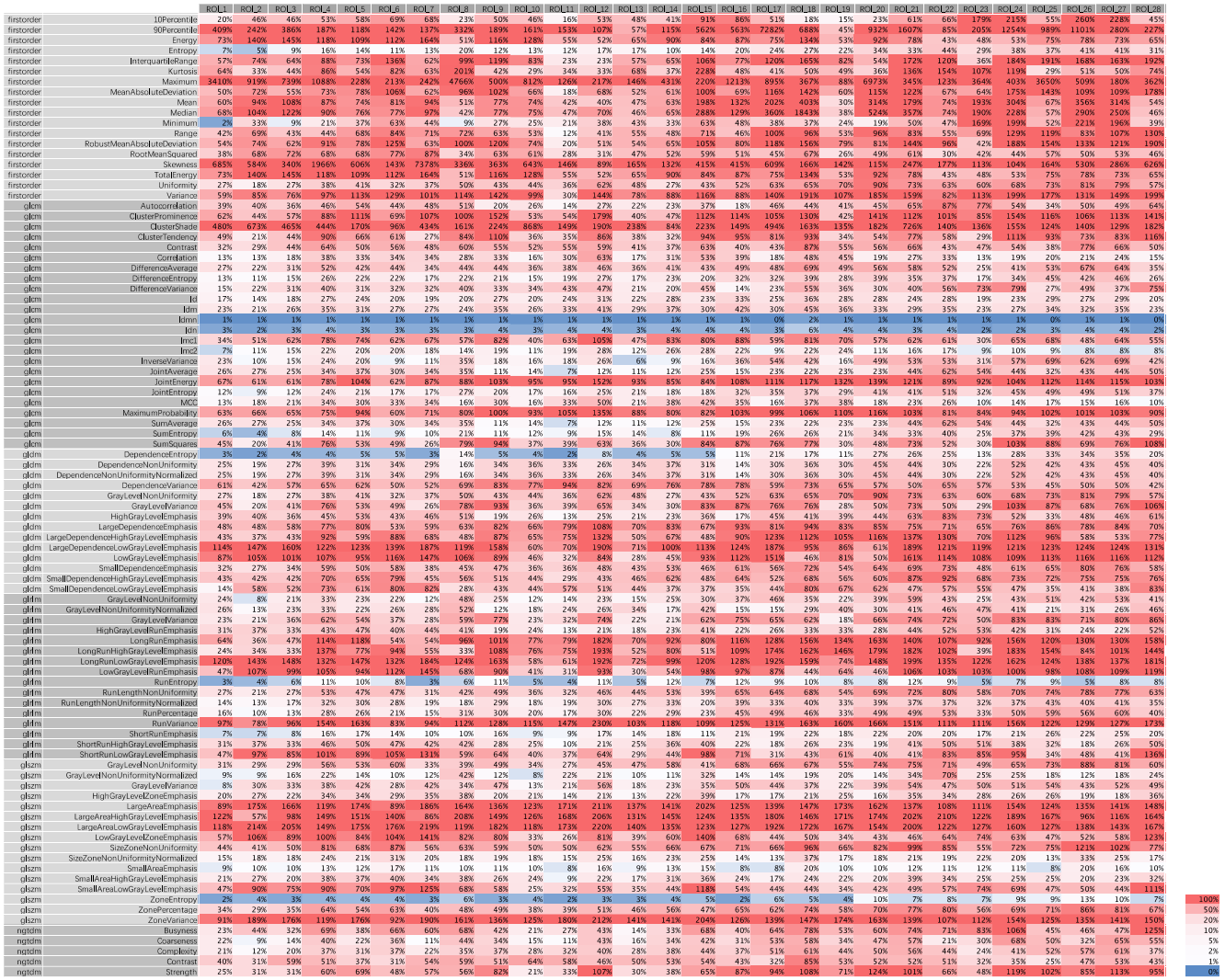

		ROI_1	ROI_2	ROI_3	ROI_4	ROI_5	ROI_6	ROI_7	ROI_8	ROI_9	ROI_10	ROI_11	ROI_12	ROI_13	ROI_14	ROI_15	ROI_16	ROI_17	ROI_18	ROI_19	ROI_20	ROI_21	ROI_22	ROI_23	ROI_24	ROI_25	ROI_26	ROI_27	ROI_28
firstorder	10Percentile	20%	46%	46%	53%	58%	69%	68%	23%	50%	46%	16%	53%	48%	41%	91%	86%	51%	18%	15%	23%	61%	66%	179%	215%	55%	260%	228%	45%
firstorder	90Percentile	409%	242%	386%	187%	118%	142%	137%	332%	189%	161%	153%	107%	57%	115%	562%	563%	7282%	688%	45%	932%	1607%	85%	205%	1254%	989%	1101%	280%	227%
firstorder	Energy	73%	140%	145%	118%	109%	112%	164%	51%	116%	128%	55%	52%	65%	90%	84%	87%	75%	134%	53%	92%	78%	43%	48%	53%	75%	78%	73%	65%
firstorder	Entropy	7%	5%	9%	16%	14%	11%	13%	20%	12%	13%	12%	17%	17%	10%	14%	20%	24%	27%	22%	34%	33%	44%	29%	38%	37%	41%	41%	31%
firstorder	InterquartileRange	57%	74%	64%	88%	73%	136%	62%	99%	119%	83%	23%	23%	57%	65%	106%	77%	120%	165%	82%	54%	172%	120%	36%	184%	191%	168%	163%	192%
firstorder	Kurtosis	64%	33%	44%	86%	54%	82%	63%	201%	42%	29%	34%	33%	68%	37%	228%	48%	41%	50%	49%	36%	136%	154%	107%	119%	29%	51%	50%	74%
firstorder	Maximum	3410%	919%	739%	1088%	228%	213%	242%	4766%	500%	812%	126%	217%	146%	431%	220%	1213%	895%	367%	88%	6973%	345%	123%	364%	403%	3650%	509%	180%	362%
firstorder	MeanAbsoluteDeviation	50%	72%	55%	73%	78%	106%	62%	96%	102%	66%	18%	68%	52%	61%	100%	69%	116%	142%	60%	115%	122%	67%	64%	175%	143%	109%	109%	178%
firstorder	Mean	60%	94%	108%	87%	74%	81%	94%	51%	77%	74%	42%	40%	47%	63%	198%	132%	202%	403%	30%	314%	179%	74%	193%	304%	67%	356%	314%	54%
firstorder	Median	68%	104%	122%	90%	76%	77%	97%	42%	77%	75%	47%	70%	46%	65%	288%	129%	360%	1843%	38%	524%	357%	74%	190%	228%	57%	290%	250%	46%
firstorder	Minimum	2%	33%	9%	21%	37%	63%	44%	9%	27%	25%	21%	38%	43%	33%	63%	48%	38%	37%	24%	19%	50%	47%	169%	199%	52%	221%	196%	39%
firstorder	Range	42%	69%	43%	44%	68%	84%	71%	72%	63%	53%	12%	41%	55%	48%	71%	46%	100%	96%	53%	96%	83%	55%	69%	129%	119%	83%	107%	130%
firstorder	RobustMeanAbsoluteDeviation	54%	74%	62%	91%	78%	125%	63%	100%	120%	74%	20%	51%	54%	65%	105%	80%	118%	156%	79%	81%	144%	96%	42%	188%	154%	133%	121%	190%
firstorder	RootMeanSquared	38%	68%	72%	68%	68%	77%	87%	34%	63%	61%	28%	31%	47%	52%	59%	51%	45%	67%	26%	49%	61%	30%	42%	44%	57%	50%	53%	46%
firstorder	Skewness	685%	584%	340%	1966%	606%	143%	7378%	336%	363%	643%	146%	89%	165%	132%	415%	415%	609%	166%	142%	115%	247%	177%	113%	104%	164%	530%	286%	626%
firstorder	TotalEnergy	73%	140%	145%	118%	109%	112%	164%	51%	116%	128%	55%	52%	65%	90%	84%	87%	75%	134%	53%	92%	78%	43%	48%	53%	75%	78%	73%	65%
firstorder	Uniformity	27%	18%	27%	38%	41%	32%	37%	50%	43%	44%	36%	62%	48%	27%	43%	52%	63%	65%	70%	90%	73%	63%	60%	68%	73%	81%	79%	57%
firstorder	Variance	59%	85%	76%	97%	113%	129%	101%	114%	142%	99%	30%	144%	78%	88%	116%	88%	140%	191%	107%	185%	159%	82%	113%	199%	177%	131%	149%	199%
glcm	Autocorrelation	39%	40%	36%	46%	54%	44%	48%	51%	20%	26%	14%	27%	22%	23%	37%	18%	46%	44%	41%	45%	65%	87%	77%	54%	34%	50%	49%	64%
glcm	ClusterProminence	62%	44%	57%	88%	111%	69%	107%	100%	152%	53%	54%	179%	40%	47%	112%	114%	105%	130%	42%	141%	112%	101%	85%	154%	116%	106%	113%	141%
glcm	ClusterShade	480%	673%	465%	444%	170%	96%	434%	161%	224%	868%	149%	190%	238%	84%	223%	149%	494%	163%	135%	182%	726%	140%	136%	155%	124%	140%	129%	182%
glcm	ClusterTendency	49%	21%	44%	90%	66%	61%	27%	84%	110%	36%	35%	86%	38%	32%	94%	95%	81%	93%	34%	54%	77%	58%	29%	111%	93%	73%	83%	116%
glcm	Contrast	32%	29%	44%	64%	50%	56%	48%	60%	55%	52%	55%	59%	41%	37%	63%	40%	43%	87%	55%	56%	66%	43%	47%	54%	38%	77%	66%	50%
glcm	Correlation	13%	13%	18%	38%	33%	34%	34%	28%	33%	16%	30%	63%	17%	31%	53%	39%	18%	48%	45%	19%	27%	33%	13%	19%	20%	21%	24%	15%
glcm	DifferenceAverage	27%	22%	31%	52%	42%	44%	34%	44%	44%	36%	38%	46%	36%	41%	43%	49%	48%	69%	49%	56%	58%	52%	25%	41%	53%	67%	64%	35%
glcm	DifferenceEntropy	13%	11%	15%	26%	22%	22%	17%	22%	21%	15%	19%	27%	17%	23%	20%	32%	32%	39%	28%	39%	35%	37%	17%	34%	45%	42%	46%	26%
glcm	DifferenceVariance	15%	22%	31%	40%	31%	32%	32%	40%	33%	34%	43%	47%	21%	20%	45%	14%	23%	55%	36%	30%	40%	56%	73%	79%	27%	49%	37%	75%
glcm	Id	17%	14%	18%	27%	24%	20%	19%	20%	27%	20%	24%	31%	22%	28%	23%	33%	25%	36%	28%	28%	24%	28%	19%	23%	29%	27%	29%	20%
glcm	Idm	23%	21%	26%	35%	31%	27%	27%	24%	35%	26%	33%	41%	29%	37%	30%	42%	30%	45%	36%	33%	29%	35%	23%	27%	34%	32%	35%	23%
glcm	Idmn	1%	1%	1%	1%	1%	1%	1%	1%	1%	1%	1%	1%	1%	1%	1%	1%	0%	2%	1%	1%	1%	1%	1%	1%	0%	1%	1%	0%
glcm	Idn	3%	2%	3%	4%	3%	3%	3%	3%	4%	3%	4%	4%	3%	4%	4%	4%	3%	6%	4%	4%	3%	4%	2%	2%	3%	4%	4%	2%
glcm	Imc1	34%	51%	62%	78%	74%	62%	67%	57%	82%	40%	63%	105%	47%	83%	80%	88%	59%	81%	70%	57%	62%	61%	30%	65%	68%	48%	64%	55%
glcm	Imc2	7%	11%	15%	22%	20%	20%	18%	14%	19%	11%	19%	28%	12%	26%	28%	22%	9%	22%	24%	11%	16%	17%	9%	10%	9%	8%	8%	8%
glcm	InverseVariance	23%	10%	15%	24%	20%	9%	11%	35%	18%	16%	18%	26%	6%	9%	16%	36%	54%	42%	16%	49%	53%	53%	31%	57%	69%	62%	69%	42%
glcm	JointAverage	26%	27%	25%	34%	37%	30%	34%	35%	11%	14%	7%	12%	11%	12%	25%	15%	23%	22%	23%	23%	44%	62%	54%	44%	32%	43%	44%	50%
glcm	JointEnergy	67%	61%	61%	78%	104%	62%	87%	88%	103%	95%	95%	152%	93%	85%	84%	108%	111%	117%	132%	139%	121%	89%	92%	104%	112%	114%	115%	103%
glcm	JointEntropy	12%	9%	12%	24%	21%	17%	17%	27%	20%	17%	16%	25%	21%	18%	18%	32%	35%	37%	29%	41%	41%	51%	32%	45%	49%	49%	51%	37%
glcm	MCC	13%	18%	21%	34%	30%	33%	34%	16%	30%	16%	33%	50%	21%	38%	42%	35%	16%	37%	38%	18%	23%	26%	10%	14%	17%	15%	16%	10%
glcm	MaximumProbability	63%	66%	65%	75%	94%	60%	71%	80%	100%	93%	105%	135%	88%	80%	82%	103%	99%	106%	110%	116%	103%	81%	84%	94%	102%	101%	103%	90%
glcm	SumAverage	26%	27%	25%	34%	37%	30%	34%	35%	11%	14%	7%	12%	11%	12%	25%	15%	23%	22%	23%	23%	44%	62%	54%	44%	32%	43%	44%	50%
glcm	SumEntropy	6%	4%	8%	14%	11%	9%	10%	21%	11%	12%	9%	15%	14%	8%	11%	19%	26%	26%	21%	34%	33%	40%	25%	37%	39%	42%	43%	29%
glcm	SumSquares	45%	20%	41%	76%	53%	49%	26%	79%	94%	37%	39%	63%	36%	30%	84%	87%	76%	77%	30%	48%	73%	52%	30%	103%	88%	69%	76%	108%
gldm	DependenceEntropy	3%	2%	4%	4%	5%	5%	3%	14%	5%	4%	2%	8%	4%	5%	5%	11%	21%	17%	11%	27%	26%	25%	13%	28%	33%	34%	35%	20%
gldm	DependenceNonUniformity	25%	19%	27%	39%	31%	34%	29%	16%	34%	36%	33%	26%	34%	37%	31%	14%	30%	36%	30%	45%	44%	30%	22%	52%	42%	43%	45%	40%
gldm	DependenceNonUniformityNormalized	25%	19%	27%	39%	31%	34%	29%	16%	34%	36%	33%	26%	34%	37%	31%	14%	30%	36%	30%	45%	44%	30%	22%	52%	42%	43%	45%	40%
gldm	DependenceVariance	61%	42%	57%	65%	62%	50%	52%	69%	83%	77%	94%	82%	69%	76%	78%	78%	59%	73%	65%	57%	50%	65%	57%	53%	45%	50%	50%	42%
gldm	GrayLevelNonUniformity	27%	18%	27%	38%	41%	32%	37%	50%	43%	44%	36%	62%	48%	27%	43%	52%	63%	65%	70%	90%	73%	63%	60%	68%	73%	81%	79%	57%
gldm	GrayLevelVariance	45%	20%	41%	76%	53%	49%	26%	78%	93%	36%	39%	65%	34%	30%	83%	87%	76%	76%	28%	50%	73%	50%	29%	103%	87%	68%	76%	106%
gldm	HighGrayLevelEmphasis	39%	40%	36%	45%	53%	43%	46%	51%	19%	26%	13%	25%	21%	23%	36%	17%	45%	41%	39%	44%	63%	83%	73%	52%	33%	48%	46%	61%
gldm	LargeDependenceEmphasis	48%	48%	58%	77%	80%	53%	59%	63%	82%	66%	79%	108%	70%	83%	67%	93%	81%	94%	83%	85%	75%	71%	65%	76%	86%	78%	84%	70%
gldm	LargeDependenceHighGrayLevelEmphasis	43%	37%	43%	92%	59%	88%	68%	48%	87%	65%	75%	132%	50%	67%	48%	90%	123%	112%	105%	116%	137%	130%	70%	112%	96%	58%	53%	77%
gldm	LargeDependenceLowGrayLevelEmphasis	114%	147%	160%	122%	123%	139%	187%	119%	158%	60%	70%	190%	71%	100%	113%	124%	187%	95%	86%	61%	189%	121%	119%	121%	123%	124%	124%	131%
gldm	LowGrayLevelEmphasis	87%	105%	101%	107%	95%	116%	147%	106%	89%	46%	32%	84%	28%	45%	93%	112%	151%	46%	81%	50%	161%	114%	108%	109%	113%	116%	116%	112%
gldm	SmallDependenceEmphasis	32%	27%	34%	59%	50%	58%	38%	45%	47%	36%	36%	48%	43%	53%	46%	61%	56%	72%	54%	64%	69%	73%	48%	61%	65%	80%	76%	58%
gldm	SmallDependenceHighGrayLevelEmphasis	43%	42%	42%	70%	65%	79%	45%	56%	51%	44%	29%	43%	46%	62%	48%	64%	52%	68%	56%	60%	87%	92%	68%	73%	72%	75%	75%	76%
gldm	SmallDependenceLowGrayLevelEmphasis	14%	58%	52%	73%	61%	80%	82%	28%	43%	44%	57%	51%	44%	37%	37%	35%	44%	80%	67%	62%	47%	57%	55%	47%	35%	41%	38%	83%
glrlm	GrayLevelNonUniformity	24%	8%	21%	33%	23%	22%	12%	48%	25%	12%	14%	23%	15%	25%	30%	37%	46%	35%	22%	39%	59%	43%	25%	43%	51%	42%	53%	41%
glrlm	GrayLevelNonUniformityNormalized	26%	13%	23%	33%	22%	26%	28%	52%	12%	18%	24%	26%	34%	17%	42%	15%	15%	29%	40%	30%	41%	46%	47%	41%	21%	31%	26%	46%
glrlm	GrayLevelVariance	23%	21%	36%	62%	54%	37%	28%	59%	77%	23%	32%	74%	22%	21%	62%	75%	65%	62%	18%	66%	74%	72%	50%	83%	83%	71%	80%	86%
glrlm	HighGrayLevelRunEmphasis	31%	37%	33%	43%	47%	40%	44%	41%	19%	24%	13%	21%	18%	23%	41%	22%	26%	33%	33%	28%	44%	52%	53%	42%	31%	24%	22%	52%
glrlm	LongRunEmphasis	64%	36%	47%	114%	118%	54%	54%	96%	101%	77%	79%	182%	70%	92%	80%	116%	128%	156%	134%	163%	140%	107%	92%	156%	120%	130%	130%	158%
glrlm	LongRunHighGrayLevelEmphasis	24%	34%	33%	137%	77%	94%	55%	33%	108%	76%	75%	193%	52%	80%	51%	109%	174%	162%	146%	179%	182%	102%	39%	183%	154%	84%	101%	144%
glrlm	LongRunLowGrayLevelEmphasis	120%	143%	148%	132%	147%	132%	184%	124%	163%	58%	61%	192%	72%	99%	120%	128%	192%	159%	74%	148%	199%	135%	122%	162%	124%	138%	137%	181%
glrlm	LowGrayLevelRunEmphasis	47%	107%	99%	105%	94%	112%	145%	68%	90%	41%	31%	93%	30%	54%	98%	92%	87%	44%	64%	46%	106%	103%	103%	100%	98%	108%	109%	119%
glrlm	RunEntropy	3%	4%	6%	11%	10%	8%	3%	6%	11%	5%	4%	11%	5%	12%	7%	12%	9%	10%	8%	8%	12%	9%	5%	7%	9%	5%	8%	8%
glrlm	RunLengthNonUniformity	27%	21%	27%	53%	47%	47%	31%	42%	49%	36%	32%	46%	44%	53%	39%	65%	64%	68%	54%	69%	72%	80%	58%	70%	74%	78%	77%	63%
glrlm	RunLengthNonUniformityNormalized	14%	13%	17%	32%	30%	28%	19%	18%	29%	18%	19%	30%	27%	33%	20%	39%	33%	40%	33%	39%	37%	37%	32%	37%	43%	40%	41%	35%
glrlm	RunPercentage	16%	10%	13%	28%	26%	21%	15%	31%	30%	20%	17%	30%	22%	29%	23%	45%	49%	46%	33%	49%	49%	53%	33%	50%	59%	56%	60%	40%
glrlm	RunVariance	97%	78%	96%	154%	163%	83%	94%	112%	126%	115%	147%	230%	103%	118%	109%	125%	131%	163%	160%	166%	151%	111%	111%	156%	122%	129%	127%	173%
glrlm	ShortRunEmphasis	7%	7%	8%	16%	17%	14%	10%	10%	16%	9%	9%	17%	14%	18%	11%	21%	19%	22%	18%	22%	20%	20%	17%	21%	26%	22%	25%	20%
glrlm	ShortRunHighGrayLevelEmphasis	31%	37%	33%	46%	50%	47%	42%	42%	28%	25%	10%	21%	25%	36%	40%	22%	18%	26%	23%	19%	41%	50%	51%	38%	32%	18%	26%	50%
glrlm	ShortRunLowGrayLevelEmphasis	47%	97%	85%	101%	89%	105%	131%	59%	64%	40%	37%	64%	29%	44%	98%	71%	31%	43%	61%	40%	41%	83%	85%	95%	34%	48%	41%	136%
glszm	GrayLevelNonUniformity	31%	29%	29%	56%	53%	60%	33%	39%	49%	34%	27%	45%	47%	58%	41%	68%	66%	67%	55%	74%	75%	71%	49%	65%	73%	88%	81%	60%
glszm	GrayLevelNonUniformityNormalized	9%	9%	16%	22%	14%	10%	12%	42%	12%	8%	22%	21%	10%	11%	32%	14%	14%	19%	20%	14%	34%	70%	25%	25%	18%	12%	18%	24%
glszm	GrayLevelVariance	8%	30%	33%	38%	42%	28%	42%	34%	47%	13%	21%	56%	18%	23%	35%	50%	44%	37%	22%	39%	54%	47%	50%	51%	54%	43%	52%	49%
glszm	HighGrayLevelZoneEmphasis	20%	27%	22%	34%	34%	29%	35%	38%	20%	21%	14%	21%	13%	22%	39%	17%	17%	21%	25%	16%	35%	34%	28%	26%	26%	19%	18%	36%
glszm	LargeAreaEmphasis	89%	175%	166%	119%	174%	89%	186%	164%	136%	123%	171%	211%	137%	141%	202%	125%	139%	147%	173%	162%	137%	108%	111%	154%	124%	135%	141%	148%
glszm	LargeAreaHighGrayLevelEmphasis	122%	57%	98%	149%	151%	140%	86%	208%	149%	126%	168%	206%	131%	145%	124%	135%	180%	146%	171%	174%	202%	210%	122%	189%	167%	96%	116%	164%
glszm	LargeAreaLowGrayLevelEmphasis	118%	214%	205%	149%	175%	176%	219%	119%	182%	118%	173%	220%	140%	135%	123%	127%	192%	172%	167%	154%	200%	122%	127%	160%	127%	138%	143%	167%
glszm	LowGrayLevelZoneEmphasis	57%	106%	89%	100%	84%	104%	141%	82%	80%	33%	26%	81%	39%	60%	140%	68%	44%	50%	34%	43%	46%	64%	74%	63%	47%	52%	58%	123%
glszm	SizeZoneNonUniformity	44%	41%	50%	81%	68%	87%	56%	63%	59%	50%	50%	62%	55%	66%	67%	71%	66%	96%	66%	82%	99%	85%	55%	72%	75%	121%	102%	77%
glszm	SizeZoneNonUniformityNormalized	15%	18%	18%	24%	21%	31%	20%	18%	19%	18%	15%	25%	16%	23%	25%	14%	13%	37%	17%	18%	21%	19%	22%	20%	13%	33%	25%	17%
glszm	SmallAreaEmphasis	9%	10%	10%	13%	12%	17%	11%	10%	11%	10%	8%	16%	9%	13%	15%	8%	8%	20%	10%	10%	12%	11%	12%	11%	8%	20%	16%	10%
glszm	SmallAreaHighGrayLevelEmphasis	21%	27%	20%	38%	37%	40%	34%	38%	26%	24%	9%	22%	17%	31%	36%	24%	17%	24%	22%	20%	39%	34%	25%	25%	25%	20%	23%	32%
glszm	SmallAreaLowGrayLevelEmphasis	47%	90%	75%	90%	70%	97%	125%	68%	58%	25%	32%	55%	35%	44%	118%	54%	44%	44%	34%	42%	49%	57%	74%	69%	47%	50%	44%	111%
glszm	ZoneEntropy	2%	4%	3%	4%	4%	4%	3%	6%	3%	4%	2%	3%	3%	4%	5%	2%	6%	5%	4%	10%	7%	8%	7%	9%	9%	13%	10%	7%
glszm	ZonePercentage	34%	29%	35%	64%	54%	63%	40%	48%	49%	38%	39%	51%	46%	56%	47%	65%	62%	74%	58%	70%	77%	80%	56%	69%	71%	86%	81%	67%
glszm	ZoneVariance	91%	189%	176%	119%	176%	92%	190%	161%	136%	125%	180%	212%	141%	141%	204%	126%	139%	147%	174%	163%	139%	107%	112%	154%	125%	135%	141%	150%
ngtdm	Busyness	23%	44%	32%	69%	38%	66%	60%	68%	42%	21%	27%	43%	14%	33%	68%	40%	64%	78%	53%	60%	74%	71%	83%	106%	45%	46%	47%	125%
ngtdm	Coarseness	22%	9%	14%	40%	22%	36%	11%	44%	34%	15%	11%	43%	16%	34%	42%	31%	53%	58%	34%	47%	57%	21%	30%	68%	50%	32%	65%	55%
ngtdm	Complexity	21%	12%	20%	37%	31%	37%	22%	35%	37%	28%	32%	40%	28%	38%	44%	37%	51%	61%	44%	50%	56%	44%	24%	41%	52%	57%	61%	37%
ngtdm	Contrast	40%	31%	59%	51%	37%	31%	54%	59%	51%	64%	58%	46%	50%	53%	54%	43%	32%	85%	53%	52%	52%	51%	32%	35%	25%	47%	53%	43%
ngtdm	Strength	25%	31%	31%	60%	69%	48%	57%	56%	82%	21%	33%	107%	30%	38%	65%	87%	94%	108%	71%	124%	101%	66%	48%	119%	102%	85%	113%	95%

Eur Radiol (2024) Zhu L, Dong H, Sun J, et al.

(D) Heatmap of inter-system variability at 10 mGy by QCD

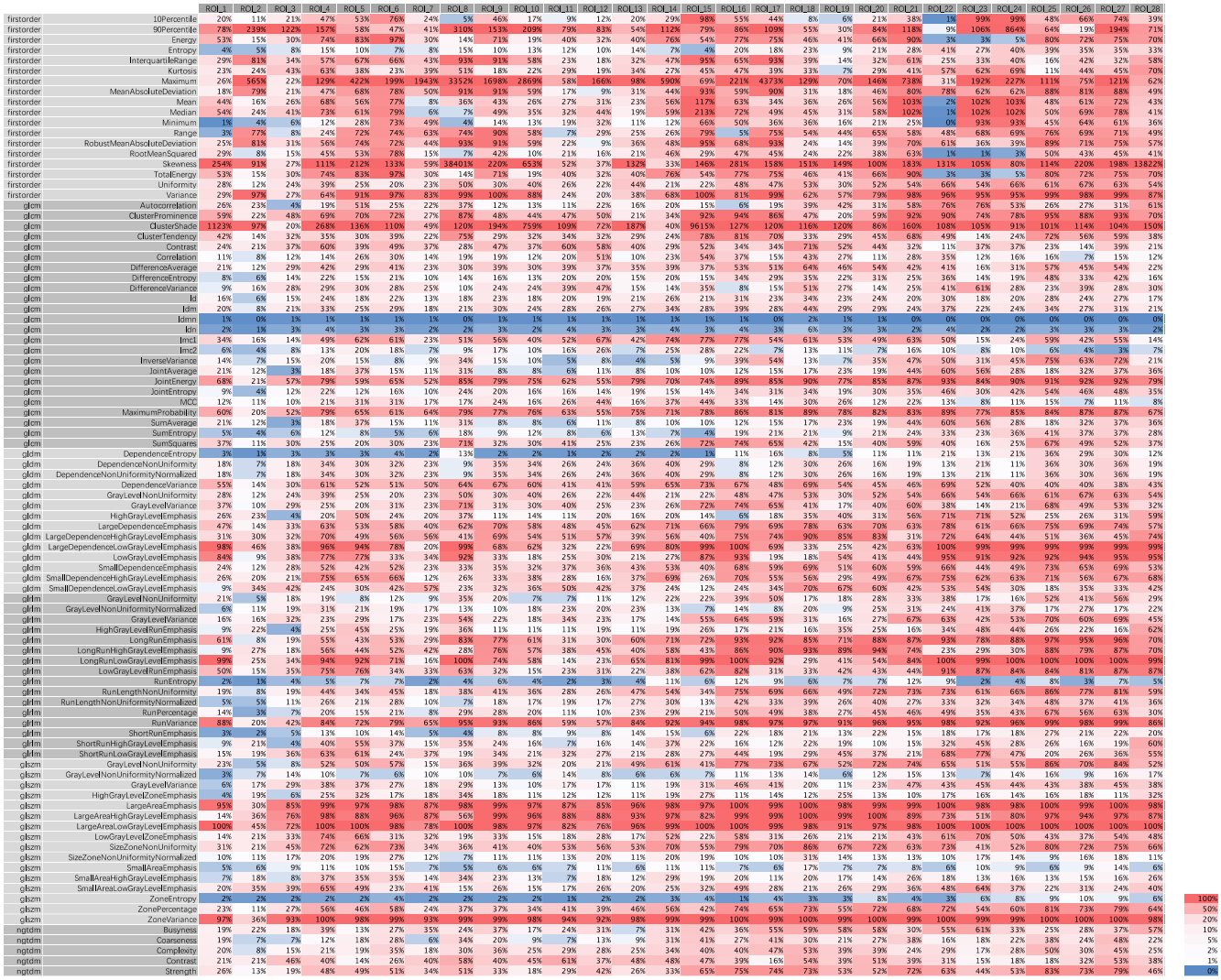

Eur Radiol (2024) Zhu L, Dong H, Sun J, et al.

(E) Heatmap of inter-system variability at 20 mGy by CV

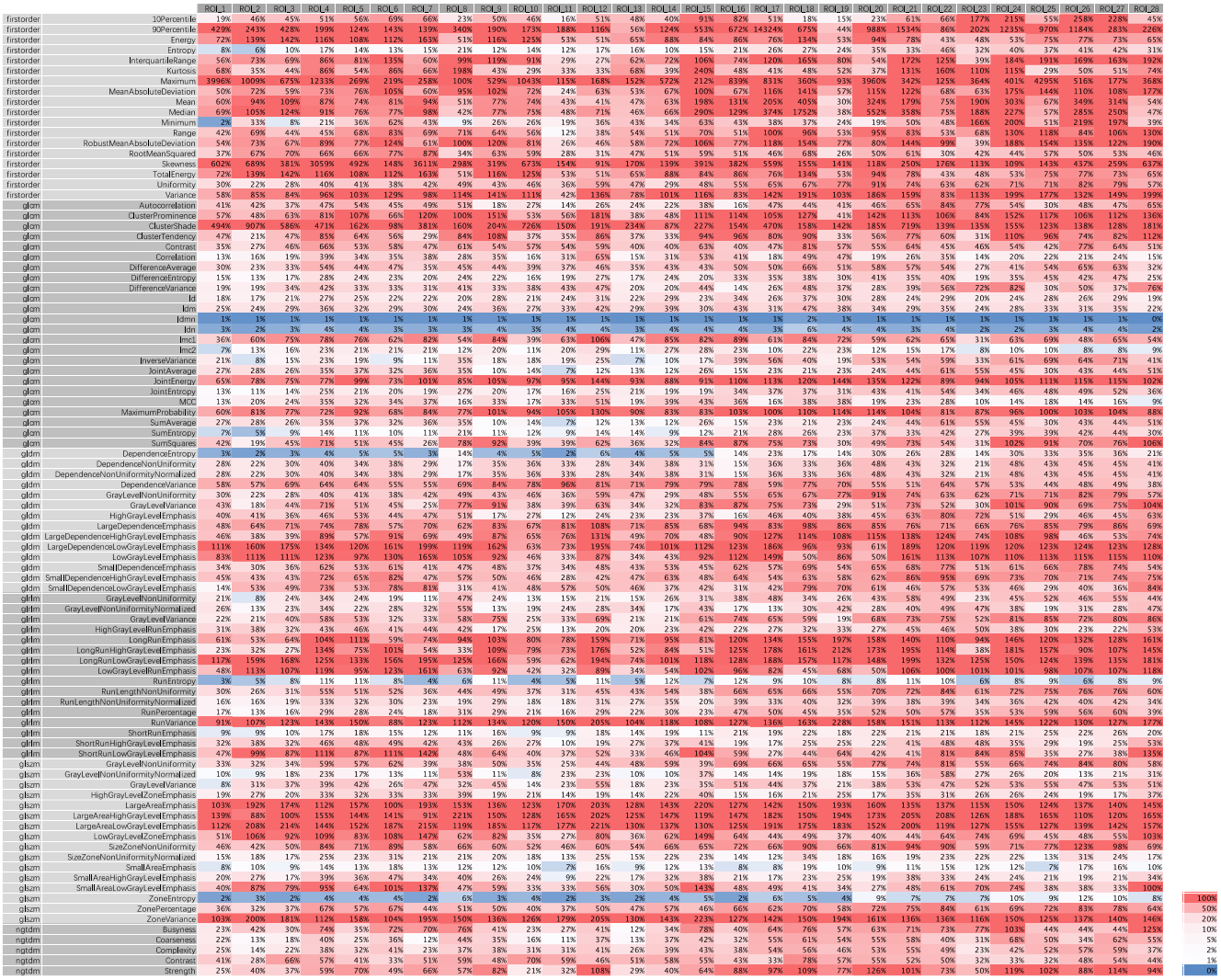

		ROI_1	ROI_2	ROI_3	ROI_4	ROI_5	ROI_6	ROI_7	ROI_8	ROI_9	ROI_10	ROI_11	ROI_12	ROI_13	ROI_14	ROI_15	ROI_16	ROI_17	ROI_18	ROI_19	ROI_20	ROI_21	ROI_22	ROI_23	ROI_24	ROI_25	ROI_26	ROI_27	ROI_28
firstorder	10Percentile	19%	46%	45%	51%	56%	69%	66%	23%	50%	46%	16%	51%	48%	40%	91%	82%	51%	18%	15%	23%	61%	66%	177%	215%	55%	258%	228%	45%
firstorder	90Percentile	429%	243%	428%	199%	124%	143%	139%	340%	190%	173%	188%	116%	56%	124%	553%	672%	14324%	675%	44%	988%	1534%	86%	202%	1235%	970%	1184%	283%	226%
firstorder	Energy	72%	139%	142%	116%	108%	112%	163%	51%	116%	125%	53%	51%	65%	88%	84%	86%	76%	134%	53%	94%	78%	43%	48%	53%	75%	77%	73%	65%
firstorder	Entropy	8%	6%	10%	17%	14%	13%	15%	21%	12%	14%	12%	17%	16%	10%	15%	21%	26%	27%	24%	35%	33%	46%	32%	40%	37%	41%	42%	31%
firstorder	InterquartileRange	56%	73%	69%	86%	81%	135%	60%	99%	119%	91%	29%	27%	62%	72%	106%	74%	120%	165%	80%	54%	172%	125%	39%	184%	191%	169%	163%	192%
firstorder	Kurtosis	68%	35%	44%	86%	54%	86%	66%	198%	43%	29%	33%	33%	68%	39%	240%	48%	41%	48%	52%	37%	131%	160%	110%	115%	29%	50%	51%	74%
firstorder	Maximum	3996%	1009%	675%	1233%	269%	219%	258%	100%	529%	1043%	115%	168%	152%	572%	212%	839%	831%	360%	93%	3960%	342%	125%	364%	401%	4295%	516%	177%	368%
firstorder	MeanAbsoluteDeviation	50%	72%	59%	73%	76%	105%	60%	95%	102%	72%	24%	63%	53%	67%	100%	67%	116%	141%	57%	115%	122%	68%	63%	175%	144%	110%	108%	127%
firstorder	Mean	60%	94%	109%	87%	74%	81%	94%	51%	77%	74%	43%	41%	47%	63%	198%	131%	205%	405%	30%	324%	179%	75%	190%	303%	67%	349%	314%	54%
firstorder	Median	69%	105%	124%	91%	76%	77%	98%	42%	77%	75%	48%	71%	46%	66%	290%	129%	374%	1752%	38%	552%	358%	75%	188%	227%	57%	285%	250%	47%
firstorder	Minimum	2%	33%	8%	21%	36%	62%	43%	9%	26%	26%	19%	36%	43%	34%	63%	43%	38%	37%	24%	19%	50%	48%	166%	200%	51%	219%	197%	39%
firstorder	Range	42%	69%	44%	45%	68%	83%	69%	71%	64%	56%	12%	38%	54%	51%	70%	51%	100%	96%	53%	95%	83%	53%	68%	130%	118%	84%	106%	130%
firstorder	RobustMeanAbsoluteDeviation	54%	73%	67%	89%	77%	124%	61%	100%	120%	81%	26%	46%	58%	72%	106%	77%	118%	154%	77%	80%	144%	90%	39%	188%	154%	135%	122%	190%
firstorder	RootMeanSquared	37%	67%	70%	66%	66%	77%	87%	34%	63%	59%	28%	31%	47%	51%	59%	51%	46%	68%	26%	50%	61%	30%	42%	44%	57%	50%	53%	46%
firstorder	Skewness	602%	689%	381%	3059%	492%	148%	3611%	298%	319%	673%	154%	91%	170%	139%	391%	382%	559%	155%	141%	118%	250%	176%	113%	109%	143%	437%	259%	637%
firstorder	TotalEnergy	72%	139%	142%	116%	108%	112%	163%	51%	116%	125%	53%	51%	65%	88%	84%	86%	76%	134%	53%	94%	78%	43%	48%	53%	75%	77%	73%	65%
firstorder	Uniformity	30%	22%	28%	40%	41%	38%	42%	49%	43%	46%	36%	59%	47%	29%	48%	55%	65%	67%	77%	91%	74%	63%	62%	71%	71%	82%	79%	57%
firstorder	Variance	58%	85%	84%	96%	103%	129%	98%	114%	141%	111%	42%	136%	78%	101%	116%	83%	142%	191%	103%	186%	159%	83%	113%	199%	127%	132%	149%	199%
glcm	Autocorrelation	41%	42%	37%	47%	54%	45%	49%	51%	18%	27%	14%	26%	24%	22%	38%	16%	47%	44%	41%	46%	65%	84%	77%	54%	30%	48%	47%	65%
glcm	ClusterProminence	57%	48%	63%	81%	107%	66%	120%	100%	151%	53%	56%	181%	38%	48%	111%	114%	105%	127%	41%	142%	113%	106%	84%	152%	117%	106%	112%	136%
glcm	ClusterShade	494%	907%	586%	471%	162%	98%	381%	160%	204%	726%	150%	191%	234%	87%	227%	154%	470%	158%	142%	185%	719%	139%	135%	155%	123%	138%	128%	181%
glcm	ClusterTendency	47%	21%	47%	85%	64%	56%	29%	84%	108%	37%	35%	86%	37%	33%	94%	96%	80%	90%	33%	56%	77%	60%	31%	110%	96%	74%	82%	112%
glcm	Contrast	35%	27%	46%	66%	53%	58%	47%	61%	54%	57%	54%	59%	40%	40%	63%	40%	47%	81%	57%	55%	64%	45%	46%	54%	42%	77%	64%	51%
glcm	Correlation	13%	16%	19%	39%	34%	35%	38%	28%	35%	16%	31%	65%	15%	31%	53%	41%	18%	49%	47%	19%	26%	35%	14%	20%	22%	21%	24%	15%
glcm	DifferenceAverage	30%	23%	33%	54%	44%	47%	35%	45%	44%	39%	37%	46%	35%	43%	43%	50%	50%	66%	51%	58%	57%	54%	27%	41%	54%	65%	63%	32%
glcm	DifferenceEntropy	15%	13%	17%	28%	24%	23%	20%	24%	22%	16%	19%	27%	17%	24%	20%	33%	35%	38%	30%	41%	35%	40%	19%	35%	45%	42%	47%	25%
glcm	DifferenceVariance	19%	19%	34%	42%	33%	33%	31%	41%	33%	38%	43%	47%	20%	20%	44%	14%	26%	48%	37%	28%	39%	56%	72%	82%	30%	50%	37%	76%
glcm	Id	18%	17%	21%	27%	25%	22%	22%	20%	28%	21%	24%	31%	22%	29%	23%	34%	26%	37%	30%	28%	24%	29%	20%	24%	28%	26%	29%	19%
glcm	Idm	25%	24%	29%	36%	32%	29%	30%	24%	36%	27%	33%	42%	29%	39%	30%	43%	31%	47%	38%	34%	29%	35%	24%	28%	33%	31%	35%	22%
glcm	Idmn	1%	1%	1%	1%	1%	1%	1%	1%	1%	1%	1%	1%	1%	1%	1%	1%	1%	2%	1%	1%	1%	1%	1%	1%	1%	1%	1%	0%
glcm	Idn	3%	2%	3%	4%	4%	3%	3%	3%	3%	4%	3%	4%	4%	3%	4%	4%	3%	6%	4%	4%	3%	4%	2%	2%	3%	4%	4%	2%
glcm	Imc1	36%	60%	75%	78%	76%	62%	82%	54%	84%	39%	63%	106%	47%	85%	82%	89%	61%	84%	72%	59%	62%	65%	31%	63%	69%	48%	65%	54%
glcm	Imc2	7%	13%	16%	23%	21%	21%	21%	12%	20%	11%	20%	29%	11%	27%	28%	23%	10%	22%	23%	12%	15%	17%	8%	10%	10%	8%	8%	9%
glcm	InverseVariance	21%	8%	15%	23%	19%	9%	11%	35%	18%	18%	19%	25%	7%	10%	17%	39%	56%	40%	19%	53%	54%	59%	33%	61%	69%	64%	71%	41%
glcm	JointAverage	27%	28%	26%	35%	37%	32%	36%	35%	10%	14%	7%	12%	13%	12%	26%	15%	23%	21%	23%	24%	44%	61%	55%	45%	30%	43%	44%	51%
glcm	JointEnergy	65%	78%	75%	77%	99%	73%	101%	85%	105%	97%	95%	144%	93%	88%	91%	110%	113%	120%	144%	135%	122%	89%	94%	105%	111%	115%	115%	102%
glcm	JointEntropy	13%	11%	14%	25%	21%	20%	19%	27%	20%	17%	16%	25%	21%	19%	19%	34%	37%	37%	31%	43%	41%	54%	34%	46%	48%	49%	52%	36%
glcm	MCC	13%	20%	24%	35%	32%	34%	37%	16%	33%	17%	33%	51%	19%	39%	43%	36%	16%	38%	38%	19%	23%	28%	10%	14%	18%	14%	16%	9%
glcm	MaximumProbability	60%	81%	77%	72%	92%	68%	84%	77%	101%	94%	105%	110%	90%	83%	83%	103%	100%	110%	114%	114%	104%	81%	87%	96%	100%	103%	104%	88%
glcm	SumAverage	27%	28%	26%	35%	37%	32%	36%	35%	10%	14%	7%	12%	13%	12%	26%	15%	23%	21%	23%	24%	44%	61%	55%	45%	30%	43%	44%	51%
glcm	SumEntropy	7%	5%	9%	14%	11%	10%	11%	21%	11%	12%	9%	14%	14%	9%	12%	21%	28%	26%	23%	37%	33%	42%	27%	39%	39%	42%	44%	30%
glcm	SumSquares	42%	19%	45%	71%	51%	45%	26%	78%	92%	39%	39%	62%	36%	32%	84%	87%	75%	73%	30%	49%	73%	54%	31%	102%	91%	70%	76%	106%
gldm	DependenceEntropy	3%	2%	3%	4%	5%	5%	3%	14%	4%	5%	2%	6%	4%	5%	5%	14%	23%	17%	14%	30%	26%	28%	14%	30%	33%	35%	36%	21%
gldm	DependenceNonUniformity	28%	22%	30%	40%	34%	38%	29%	17%	35%	36%	33%	28%	34%	38%	31%	15%	36%	33%	36%	48%	43%	32%	21%	48%	43%	45%	45%	41%
gldm	DependenceNonUniformityNormalized	28%	22%	30%	40%	34%	38%	29%	17%	35%	36%	33%	28%	34%	38%	31%	15%	36%	33%	36%	48%	43%	32%	21%	48%	43%	45%	45%	41%
gldm	DependenceVariance	58%	57%	69%	64%	64%	55%	55%	69%	84%	78%	96%	81%	71%	79%	79%	78%	59%	77%	70%	55%	51%	64%	57%	53%	44%	48%	49%	38%
gldm	GrayLevelNonUniformity	30%	22%	28%	40%	41%	38%	42%	49%	43%	46%	36%	59%	47%	29%	48%	55%	65%	67%	77%	91%	74%	63%	62%	71%	71%	82%	79%	57%
gldm	GrayLevelVariance	43%	18%	44%	71%	51%	45%	25%	77%	91%	38%	39%	63%	34%	32%	83%	87%	75%	73%	29%	51%	73%	52%	30%	101%	90%	69%	75%	104%
gldm	HighGrayLevelEmphasis	40%	41%	36%	46%	53%	44%	47%	51%	17%	27%	12%	24%	23%	23%	37%	16%	46%	40%	38%	45%	63%	80%	72%	51%	29%	46%	45%	63%
gldm	LargeDependenceEmphasis	48%	64%	71%	74%	78%	57%	70%	62%	83%	67%	81%	108%	71%	85%	68%	94%	83%	98%	86%	85%	76%	71%	66%	76%	85%	78%	86%	69%
gldm	LargeDependenceHighGrayLevelEmphasis	46%	38%	39%	89%	57%	91%	69%	49%	87%	65%	76%	131%	49%	70%	48%	90%	127%	114%	108%	115%	138%	124%	74%	108%	98%	46%	53%	74%
gldm	LargeDependenceLowGrayLevelEmphasis	111%	160%	175%	134%	120%	161%	199%	119%	162%	63%	73%	195%	74%	101%	112%	123%	186%	96%	93%	61%	189%	120%	119%	120%	123%	124%	123%	128%
gldm	LowGrayLevelEmphasis	83%	111%	111%	123%	97%	130%	165%	105%	92%	46%	33%	87%	34%	43%	92%	112%	149%	50%	86%	50%	161%	113%	107%	110%	113%	115%	115%	110%
gldm	SmallDependenceEmphasis	34%	30%	36%	62%	53%	61%	41%	47%	48%	37%	34%	48%	43%	53%	45%	62%	57%	69%	54%	65%	68%	77%	51%	61%	66%	78%	74%	54%
gldm	SmallDependenceHighGrayLevelEmphasis	45%	43%	43%	72%	65%	82%	47%	57%	50%	46%	28%	42%	42%	63%	48%	64%	54%	63%	58%	62%	86%	95%	69%	73%	70%	71%	74%	75%
gldm	SmallDependenceLowGrayLevelEmphasis	14%	53%	49%	73%	53%	78%	81%	31%	41%	48%	57%	50%	46%	37%	42%	31%	42%	79%	70%	61%	46%	57%	53%	46%	29%	40%	36%	84%
glrlm	GrayLevelNonUniformity	21%	8%	24%	34%	24%	19%	11%	47%	24%	13%	15%	21%	15%	26%	31%	38%	48%	34%	26%	43%	58%	49%	23%	45%	52%	46%	55%	44%
glrlm	GrayLevelNonUniformityNormalized	26%	13%	23%	34%	22%	28%	32%	55%	13%	19%	24%	28%	34%	17%	43%	17%	13%	30%	42%	28%	40%	49%	47%	38%	19%	31%	28%	47%
glrlm	GrayLevelVariance	22%	21%	40%	58%	53%	32%	33%	58%	75%	25%	33%	69%	21%	21%	61%	74%	65%	59%	19%	68%	73%	75%	52%	81%	85%	72%	80%	86%
glrlm	HighGrayLevelRunEmphasis	31%	38%	32%	43%	46%	41%	44%	42%	17%	25%	13%	20%	20%	23%	42%	22%	27%	32%	33%	27%	45%	46%	50%	38%	30%	23%	22%	53%
glrlm	LongRunEmphasis	61%	53%	64%	104%	111%	59%	74%	94%	103%	80%	78%	159%	71%	95%	81%	120%	134%	155%	197%	158%	140%	110%	94%	146%	120%	132%	128%	161%
glrlm	LongRunHighGrayLevelEmphasis	23%	32%	27%	134%	75%	101%	54%	33%	109%	79%	73%	176%	52%	84%	51%	125%	178%	161%	212%	173%	195%	114%	38%	181%	157%	90%	107%	145%
glrlm	LongRunLowGrayLevelEmphasis	117%	159%	168%	125%	133%	156%	195%	125%	166%	59%	62%	194%	74%	101%	118%	128%	188%	157%	117%	148%	199%	132%	125%	150%	124%	139%	135%	181%
glrlm	LowGrayLevelRunEmphasis	48%	113%	107%	119%	95%	123%	161%	63%	92%	42%	32%	89%	34%	54%	102%	96%	82%	45%	68%	50%	106%	100%	101%	101%	98%	107%	107%	118%
glrlm	RunEntropy	3%	5%	8%	11%	11%	8%	4%	6%	11%	4%	5%	11%	5%	12%	7%	12%	9%	10%	8%	8%	11%	10%	6%	8%	9%	6%	8%	9%
glrlm	RunLengthNonUniformity	30%	26%	31%	55%	51%	52%	36%	44%	49%	37%	31%	45%	43%	54%	38%	66%	65%	66%	55%	70%	72%	84%	61%	72%	75%	76%	76%	60%
glrlm	RunLengthNonUniformityNormalized	16%	16%	19%	33%	32%	30%	23%	19%	29%	18%	18%	31%	27%	35%	20%	39%	33%	40%	32%	39%	38%	39%	34%	36%	42%	40%	42%	34%
glrlm	RunPercentage	17%	13%	16%	29%	28%	24%	18%	31%	29%	21%	16%	29%	22%	30%	23%	47%	50%	45%	35%	52%	50%	57%	35%	53%	59%	56%	60%	39%
glrlm	RunVariance	91%	107%	123%	143%	150%	88%	123%	112%	134%	120%	150%	205%	104%	118%	108%	127%	136%	163%	228%	158%	151%	113%	112%	145%	122%	136%	127%	172%
glrlm	ShortRunEmphasis	9%	9%	10%	17%	18%	15%	12%	11%	16%	9%	9%	18%	14%	19%	11%	21%	19%	22%	18%	22%	21%	21%	18%	21%	25%	22%	26%	20%
glrlm	ShortRunHighGrayLevelEmphasis	32%	38%	32%	46%	48%	49%	42%	43%	26%	27%	10%	19%	27%	37%	41%	19%	17%	25%	25%	22%	41%	48%	48%	35%	29%	19%	25%	53%
glrlm	ShortRunLowGrayLevelEmphasis	47%	99%	87%	111%	87%	111%	142%	48%	64%	40%	37%	52%	33%	46%	104%	59%	27%	44%	64%	42%	41%	81%	84%	85%	35%	27%	38%	135%
glszm	GrayLevelNonUniformity	33%	32%	34%	59%	57%	62%	39%	38%	50%	35%	25%	44%	48%	59%	39%	69%	66%	65%	55%	77%	74%	81%	55%	66%	74%	84%	80%	58%
glszm	GrayLevelNonUniformityNormalized	10%	9%	18%	23%	17%	13%	11%	53%	11%	8%	23%	23%	10%	10%	37%	14%	14%	19%	18%	15%	36%	58%	27%	26%	20%	13%	21%	31%
glszm	GrayLevelVariance	8%	11%	17%	39%	42%	26%	47%	32%	45%	14%	23%	55%	18%	23%	35%	51%	44%	37%	21%	38%	53%	47%	52%	53%	55%	47%	53%	51%
glszm	HighGrayLevelZoneEmphasis	19%	27%	20%	33%	32%	33%	33%	39%	19%	21%	14%	19%	14%	22%	40%	15%	16%	21%	25%	17%	35%	31%	26%	26%	24%	19%	17%	37%
glszm	LargeAreaEmphasis	103%	192%	174%	112%	157%	100%	193%	153%	136%	123%	170%	203%	128%	143%	220%	127%	142%	150%	193%	160%	135%	137%	115%	150%	124%	137%	140%	145%
glszm	LargeAreaHighGrayLevelEmphasis	139%	88%	100%	155%	144%	141%	91%	221%	150%	128%	165%	202%	125%	147%	119%	147%	182%	150%	194%	173%	205%	208%	126%	188%	165%	110%	120%	165%
glszm	LargeAreaLowGrayLevelEmphasis	112%	208%	214%	144%	152%	187%	215%	119%	185%	117%	177%	221%	130%	137%	130%	125%	191%	175%	183%	152%	200%	119%	127%	155%	127%	139%	142%	157%
glszm	LowGrayLevelZoneEmphasis	51%	106%	92%	109%	83%	108%	147%	62%	82%	35%	27%	80%	36%	62%	149%	64%	44%	49%	37%	40%	44%	64%	74%	69%	45%	48%	55%	103%
glszm	SizeZoneNonUniformity	46%	42%	50%	84%	71%	89%	58%	66%	60%	52%	46%	60%	54%	66%	65%	72%	66%	90%	66%	81%	94%	90%	59%	71%	77%	123%	98%	69%
glszm	SizeZoneNonUniformityNormalized	15%	18%	17%	25%	23%	31%	21%	21%	20%	18%	13%	25%	15%	22%	23%	14%	12%	34%	18%	16%	19%	23%	22%	22%	13%	31%	24%	17%
glszm	SmallAreaEmphasis	8%	10%	9%	14%	13%	18%	13%	12%	12%	10%	7%	16%	9%	12%	13%	8%	8%	19%	10%	9%	11%	15%	12%	12%	7%	17%	16%	10%
glszm	SmallAreaHighGrayLevelEmphasis	20%	27%	17%	39%	36%	47%	34%	40%	26%	24%	9%	22%	17%	32%	38%	21%	17%	23%	25%	19%	38%	33%	24%	24%	21%	19%	21%	34%
glszm	SmallAreaLowGrayLevelEmphasis	40%	87%	79%	95%	64%	101%	137%	47%	59%	33%	33%	56%	30%	50%	143%	48%	49%	41%	34%	27%	48%	61%	70%	74%	38%	38%	33%	100%
glszm	ZoneEntropy	2%	3%	2%	4%	4%	4%	2%	6%	3%	4%	2%	3%	2%	4%	5%	2%	6%	5%	4%	9%	7%	7%	7%	7%	10%	9%	12%	10%	8%
glszm	ZonePercentage	36%	32%	37%	67%	57%	67%	44%	51%	50%	40%	37%	50%	47%	57%	46%	66%	62%	70%	58%	72%	75%	84%	61%	69%	72%	83%	78%	64%
glszm	ZoneVariance	103%	200%	181%	112%	158%	104%	195%	150%	136%	126%	179%	205%	130%	143%	223%	127%	142%	150%	194%	161%	136%	136%	116%	150%	125%	137%	140%	146%
ngtdm	Busyness	23%	42%	30%	74%	35%	72%	70%	76%	41%	23%	27%	41%	12%	34%	78%	40%	64%	76%	57%	63%	71%	73%	77%	103%	44%	44%	44%	125%
ngtdm	Coarseness	22%	13%	18%	40%	25%	36%	12%	44%	35%	16%	11%	37%	13%	37%	42%	32%	55%	61%	54%	55%	58%	40%	31%	68%	50%	34%	62%	55%
ngtdm	Complexity	25%	14%	22%	38%	32%	41%	23%	37%	38%	31%	31%	41%	26%	39%	43%	38%	54%	56%	46%	53%	55%	49%	23%	42%	52%	57%	59%	37%
ngtdm	Contrast	41%	28%	66%	57%	41%	33%	51%	59%	48%	70%	59%	46%	51%	58%	55%	43%	33%	78%	57%	55%	52%	50%	32%	33%	32%	48%	54%	44%
ngtdm	Strength	25%	40%	37%	59%	70%	49%	66%	57%	82%	21%	32%	108%	29%	40%	64%	88%	97%	109%	77%	126%	101%	73%	50%	119%	102%	88%	114%	94%

(F) Heatmap of inter-system variability at 20 mGy by QCD

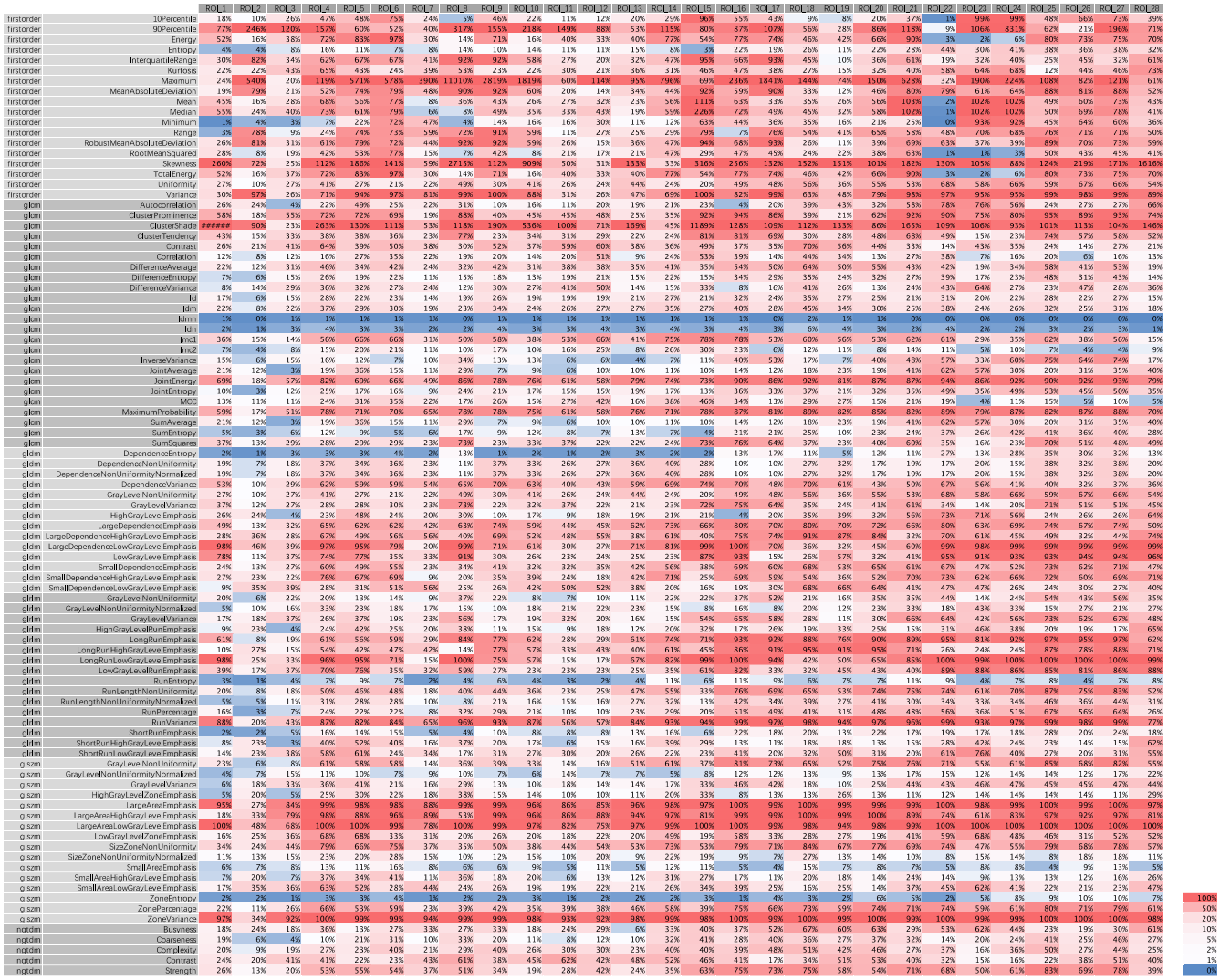

Eur Radiol (2024) Zhu L, Dong H, Sun J, et al.
